# Imidazolium Chloride: An Efficient Catalyst for Transamidation of Primary Amines

**DOI:** 10.3390/molecules23092234

**Published:** 2018-09-02

**Authors:** Qingqiang Tian, Zongjie Gan, Xuetong Wang, Dan Li, Wen Luo, Huajun Wang, Zeshu Dai, Jianyong Yuan

**Affiliations:** College of Pharmacy, Chongqing Medical University, Chongqing 400016, China; Tqingqiang@163.com (Q.T.); gzj@cqmu.edu.cn (Z.G.); wxy998298@163.com (X.W.); ld28115@163.com (D.L.); lwen1129@163.com (W.L.); jonh_wong@163.com (H.W.); daizs11@163.com (Z.D.)

**Keywords:** imidazolium chloride, catalyst, primary amines, transamidation

## Abstract

A highly efficient and convenient protocol of imidazolium chloride (30 mol %) catalyzed amidation of amines with moderate to excellent yields was reported. The protocol shows broad substrate scope for aromatic, aliphatic, and heterocyclic primary amines.

## 1. Introduction

**Figure 1:**
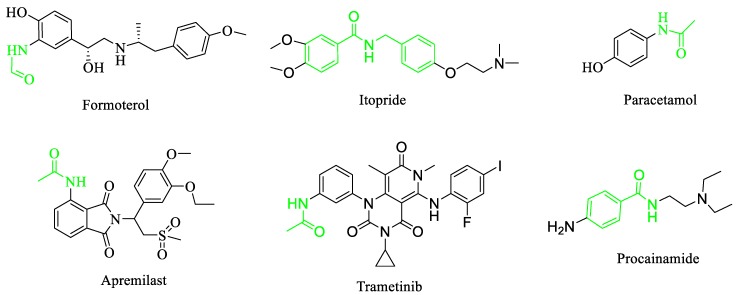
Amide bonds in some drug molecules.

Formamide and acetamide are common organic fragments in organic compounds. An amide bond is considered to be one of the most important linkages from a medicinal point of view [1,2,3,4]. In addition, amide is also an important structural core of many FDA approved drugs, such as formoterol [5], itopride [6], trametinib [7], apremilast [8], paracetamol [9], and procainamide [10] (Figure 1). Therefore, finding new methods of amidation would definitely be of major benefit for the drug discovery process. Generally, the classical method of preparing acetamide involves the reaction of an amine with a carboxylic acid derivative [11,12,13]. Alternatively, transamidation has been proved to be an attractive tool and represents one of the most convenient and straightforward methods for the preparation of acetamide [14].

**Scheme 1:**
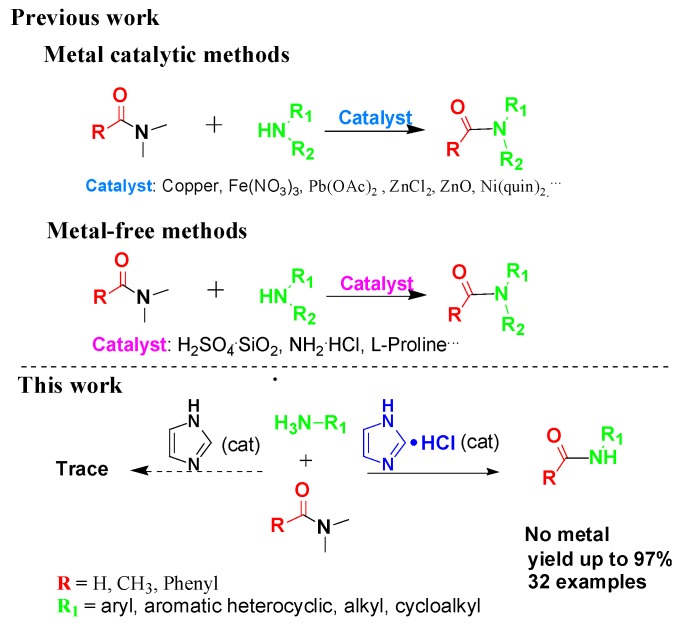
Methods for transamidation.

Stahl [15,16,17,18,19], Williams [20,21], Myers [22], Beller [23], and other groups [24,25,26,27,28] have developed many transamidation synthetic methods employing metallic catalysts such as copper [29], Fe (NO_3_)_3_ [30], Pd (OAc)_2_ [31], ZnCl_2_ [32], ZnO [33], and Ni(quin)_2_ [34] as promoters. On the other hand, metal-free methods utilizing catalytic hydroxylamine hydrochloride [35], L-proline [36], and H_2_SO_4_^.^SiO_2_ [37] have also been reported. Recently, Hudson et al. [38] have developed an alternative and elegant protocol for the *N*-formylation of amino acid ester hydrochloride with *N*,*N*-Dimethylformamide (DMF) employing imidazole as the catalyst. However, further research showed that this synthesis method was not compatible with aromatic amine or aliphatic amine substrates. In view of this, herein, we wish to report a general imidazolium chloride catalyzed transamidation of aromatic or aliphatic amines with satisfactory results (Scheme 1).

## 2. Results and Discussion

In preliminary reactions, *p*-toluidine was treated with DMA in the presence of various protic acids or bases such as HAc, PTSA, or Et_3_N at 150 °C for 6 h, however, with resulting low yields of acetamide (Table 1, entries 4–11). Interestingly, when a reaction was carried out with imidazolium chloride (Table 1, entry 12) at room temperature, traces of product formation was observed. Encouraged by this result, the reaction was carried with 3 equiv. imidazolium chloride under moderate conditions. Upon varying the temperature between 30 °C and 150 °C, the transamidation product increased. No target product was detected at 30 °C (Table 1, entry 17), and 59% yield of product was obtained at 60 °C (Table 1, entry 18), while a satisfactory yield (96%) was observed at 150 °C (Table 1, entry 13), indicating that the reaction was highly sensitive to temperature. Subsequently, the reaction was also found to be efficient with 0.3 equiv. imidazolium chloride, affording a yield of the product with 96% within 2 h (Table 1, entries 15). Further optimization showed that reducing the amount of imidazolium chloride decreased the yield of compound (Table 1, entry 16) and no significant effect on reactivity was observed when the loading was raised from 0.3 to 0.5 equiv. (Table 1, entries 13 and 14). Additionally, the reaction failed to proceed in the absence of imidazolium chloride, suggesting the imidazolium chloride complex may be responsible for the activation of DMA (Table 1, entry 22). 

Having established the optimal reaction conditions (Table 1, entry 15), we next set out to examine the scope and limitations of this reaction, as shown in Table 2. *N*,*N*-Dimethylacetamide (DMA) was chosen as the acetyl donor and a variety of primary amines were studied. To our delight, the aromatic primary amines bearing electron-donating and -withdrawing groups were tolerated well under the reaction conditions, affording the desired products in moderate to good yields (Table 2, entries 1–14). It was noteworthy that the electronic properties of the substituent groups on the phenyl ring played an important role in the reaction. Aromatic primary amine containing electron-donating groups provided the desired *N*-acetamide product in better yields than those of electron-withdrawing groups (Table 2, entries 2, 3, and 11 vs. entries 12, 13, and 14). The halogen substituted aniline (m/p/o) showed more reactivity in comparison with that of *p*-nitroaniline and gave corresponding products in relatively high yields (Table 2, entries 4–6 and 7–9 vs. entry 12). Benzlyamines with electron-rich and -deficient substituents were reacted smoothly and produced corresponding transamidation products in good to excellent yields as well (Table 2, entries 15 and 16). In addition, the heterocyclic amines and the aliphatic amines were also compatible and afforded the corresponding products in moderate to good yields (Table 2, entries 17–23).

In order to explore the scope of transamidation, a variety of aromatic, aliphatic, or benzylic amines were conducted to react with DMF (Table 3) and *N*,*N*-Dimethylbenzamide (Table 4). In general, the transamidation of formide with aromatic amines and benzylic amines (electron-deficient, -neutral, -rich) gave corresponding transamidation products in moderate to good yields (**2a**–**2e**). Similarly, transamidation of benzamide with *N*,*N*-Dimethylbenzamide also gave **3a**–**3d** in excellent yields. It should be noted that the acylation products can be obtained with good quality and purity by a simple filtration procedure in most cases.

To demonstrate the practical utility, the amidation of 4-aminophenol with DMA was carried out on a 50 g scale under the same conditions to give 4-acetamidophenol in 91% satisfactory yield after silica gel chromatography.

**Scheme 2:**
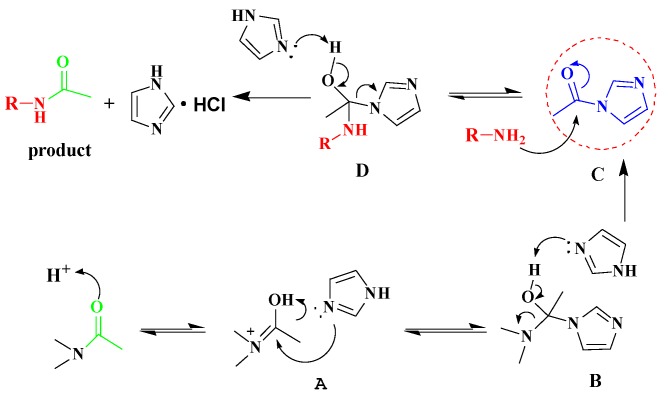
Plausible mechanism for *N*-acetylation using imidazolium chloride catalyst.

Based on our experimental results and previous studies [38], a plausible mechanism is proposed in Scheme 2. Firstly, DMA was activated by H^+^ to afford intermediate A, which was attacked by imidazole, resulting in the formation of intermediate B; HNMe_2_ was removed, leading to the formation of the key intermediate C. Next, nucleophilic addition of the primary amine generates a tetrahedral intermediate D which undergoes an elimination of imidazole to afford the target compound.

## 3. Materials and Methods

### 3.1. General Information

All reactions were carried out under normal conditions and no any stringent conditions were used. All reagents were obtained from Aladdin Reagent Co., Ltd. (Shanghai, China), Lagewell Technology Co., Ltd. (Shenzhen, China), Meyer Reagent Co., Ltd. (Shanghai, China), Macklin Reagent Co., Ltd. (Shanghai, China), Chongqing Chuandong Chemical Co., Ltd. (Chongqing, China). etc. without further purification unless otherwise noted. Reactions were monitored by TLC analysis using Merck Silica Gel 60 F-254 thin layer plates. The plates were visualized first with short wavelength UV light followed by iodine stain. ^1^H and ^13^C NMR spectra were recorded in CDCl_3_ and DMSO-*d*_6_ on a Bruker Ascend-III 600 MHz spectrometer using TMS as an internal standard. The residual solvent signals were used as references and the chemical shifts converted to the TMS scale (CDCl_3_: δ H = 7.25–7.30 ppm, δ C = 77.23 ppm; DMSO-*d*_6_: δ H = 2.51 ppm, δ C = 39.51 ppm).

### 3.2. General Procedures for the Synthesis of N-Acetamides ***1a**–**1w***

To a mixture of aromatic or aliphatic or heterocyclic amine (3.0 mmol, 1.0 equiv.), Imidazolium chloride (1.0 mmol, 0.3 equiv.), *N*,*N*-Dimethyl acetamide (2.0 mL) was added. The mixture was refluxed at 150 °C and the progress of the reaction was monitored by TLC visualized with UV short wavelength followed by iodine stain. After completion, the mixture was diluted with cold water (10 mL) then extracted with EtOAc (10 mL). The EtOAc layer was washed with 1 M hydrochloric acid (3.0 × 15 mL). Adsorption of pigment with activated carbon, filtration of filtrate. The filtrate was dried over anhydrous Na_2_SO_4_ and concentrated under vacuum to obtain crude *N*-acetamide amine, the *N*-acetamide amine product was isolated by column chromatography eluting with petroleum ether:ethyl acetate (10:1) mixtures.

### 3.3. General Procedures for the Synthesis of N-Formamides ***2a**–**2e***

To a mixture of aromatic or aliphatic or heterocyclic amine (3.0 mmol, 1.0 equiv.), Imidazolium chloride (1.0 mmol, 0.3 equiv.), *N*,*N*-Dimethyl formamide (2.0 mL) was added. The mixture was refluxed at 150 °C and the progress of the reaction was monitored by TLC visualized with UV short wavelength followed by iodine stain. After completion, the mixture was diluted with cold water (10 mL) then extracted with EtOAc (10 mL). The EtOAc layer was washed with 1 M hydrochloric acid (3.0 × 15 mL). Adsorption of pigment with activated carbon, filtration of filtrate. The filtrate was dried over anhydrous Na_2_SO_4_ and concentrated under vacuum to obtain crude N-formyl amine, the N-formyl amine product was isolated by column chromatography eluting with petroleum ether: ethyl acetate (10:1) mixtures.

### 3.4. General Procedures for the Synthesis of N-Benzoylation ***3a**–**3d***

To a mixture of aromatic or aliphatic or heterocyclic amine (3.0 mmol, 1.0 equiv), Imidazolium chloride (1.0 mmol, 0.3 equiv.), *N*,*N*-Dimethylbenzamide (2.0 equiv.) was added. The mixture was refluxed at 150 °C and the progress of the reaction was monitored by TLC visualized with UV short wavelength followed by iodine stain. After completion, the mixture was diluted with cold water (10 mL). The crystallized solid were filtered and washed with water and heptane, dried under vacuum to give the product.

*N-Phenylacetamide* (**1a**): The product was obtained as off-white solid in 92% yield (0.37 g); MP: 112–115 °C; ^1^H NMR (600 MHz, CDCl_3_) δ 7.82 (s, 1H), 7.54–7.47 (m, 2H), 7.29 (dd, *J* = 8.5, 7.4 Hz, 2H), 7.12–7.07 (m, 1H), 2.15 (s, 3H); ^13^C NMR (151 MHz, CDCl_3_) δ 168.74, 137.90, 128.94, 124.29, 119.97, 24.43.

*N-p-Tolylacetamide* (**1b**): The product was obtained as pale yellow solid in 97% yield (0.43 g); MP: 150–153 °C; ^1^H NMR (600 MHz, DMSO-*d*_6_) δ 9.83 (s, 1H), 7.46 (dd, *J* = 8.7, 2.4 Hz, 2H), 7.08 (d, *J* = 8.3 Hz, 2H), 2.24 (s, 3H), 2.02 (s, 3H); ^13^C NMR (151 MHz, DMSO-*d*_6_) δ 168.93, 136.84, 132.67, 129.52, 119.56, 24.16, 20.81.

*N-m-Tolyl-acetamide* (**1c**): The product was obtained as dark brown solid in 90% yield (0.40 g); MP: 66–68 °C; ^1^H NMR (600 MHz, DMSO-*d*_6_) δ 9.91 (s, 1H), 7.43 (d, *J* = 2.0 Hz, 1H), 7.40–7.35 (m, 1H), 7.18 (t, *J* = 7.8 Hz, 1H), 6.89–6.84 (m, 1H), 2.27 (s, 3H), 2.05 (s, 3H); ^13^C NMR (151 MHz, DMSO-*d*_6_) δ 168.82, 139.47, 138.30, 128.94, 124.24, 119.96, 116.63, 24.32, 21.60.

*N-(2-Chloro-phenyl)-acetamide* (**1d**): The product was obtained as off-white solid in 75% yield (0.38 g); MP: 87–88 °C; ^1^H NMR (600 MHz, CDCl_3_) δ 8.35 (d, *J* = 8.4 Hz, 1H), 7.64 (s, 1H), 7.36 (d, *J* = 8.0 Hz, 1H), 7.27 (t, *J* = 7.8 Hz, 1H), 7.03 (t, *J* = 7.7 Hz, 1H), 2.24 (s, 3H); ^13^C NMR (151 MHz, CDCl_3_) δ 168.26, 134.61, 128.96, 127.73, 124.62, 122.55, 121.66, 24.88.

*N-(3-Chlorophenyl)acetamide* (**1e**): The product was obtained as pale yellowish-brown solid in 82% yield (0.42 g); MP: 74–77 °C; ^1^H NMR (600 MHz, DMSO-*d*_6_) δ 10.13 (s, 1H), 7.94–7.68 (m, 1H), 7.57–7.35 (m, 1H), 7.36–7.27 (m, 1H), 7.14–7.02 (m, 1H), 2.06 (d, *J* = 0.8 Hz, 3H); ^13^C NMR (151 MHz, DMSO-*d*_6_) δ 169.17, 141.01, 133.50, 130.80, 123.17, 118.80, 117.67, 24.39.

*N-(4-Chlorophenyl)acetamide* (**1f**): The product was obtained as pale brown solid in 84% yield (0.43 g); MP: 175–177 °C; ^1^H NMR (600 MHz, DMSO-*d*_6_) δ 10.07 (s, 1H), 7.71–7.48 (m, 2H), 7.48–7.23 (m, 2H), 2.04 (d, *J* = 1.3 Hz, 3H); ^13^C NMR (151 MHz, DMSO-*d*_6_) δ 169.00, 138.48, 138.46, 129.01, 127.06, 120.93, 24.31.

*N-(3-Bromo-phenyl)-acetamide* (**1g**): The product was obtained as pale brown solid in 83% yield (0.53 g); MP: 85–87 °C; ^1^H NMR (600 MHz, DMSO-*d*_6_) δ 10.11 (s, 1H), 7.95 (d, *J* = 1.9 Hz, 1H), 7.47–7.44 (m, 1H), 7.26 (t, *J* = 8.0 Hz, 1H), 7.24–7.19 (m, 1H), 2.05 (s, 3H); ^13^C NMR (151 MHz, DMSO-*d*_6_) δ 169.24, 141.11, 131.17, 126.12, 121.98, 121.65, 118.09, 24.38.

*N-(2-Bromo-phenyl)-acetamide* (**1h**):The product was obtained as pale brown solid in 80% yield (0.51 g); MP: 97–99 °C; ^1^H NMR (600 MHz, CDCl_3_) δ 8.33 (d, *J* = 8.3 Hz, 1H), 7.61 (s, 1H), 7.53 (dd, *J* = 8.0, 1.4 Hz, 1H), 7.31 (ddd, *J* = 8.5, 7.4, 1.5 Hz, 1H), 7.01–6.92 (m, 1H), 2.24 (s, 3H); ^13^C NMR (151 MHz, CDCl_3_) δ 168.17, 135.59, 132.19, 128.39, 125.16, 121.87, 113.10, 24.83.

*N-(4-Bromophenyl)-acetamide* (**1i**): The product was obtained as pale yellowish-brown solid in 89% yield (0.57 g); MP: 166–169 °C; ^1^H NMR (600 MHz, DMSO-*d*_6_) δ 10.07 (s, 1H), 7.56 (d, *J* = 8.8 Hz, 2H), 7.47 (d, *J* = 8.8 Hz, 2H), 2.05 (s, 3H); ^13^C NMR (151 MHz, DMSO-*d*_6_) δ 169.12, 138.83, 131.91, 121.36, 115.10, 24.31.

*N-(4-Bromo-2-methyl-phenyl)-acetamide* (**1j**): The product was obtained as off-white solid in 90% yield (0.61 g); MP:160–162 °C; ^1^H NMR (600 MHz, CDCl_3_) δ 7.68 (d, *J* = 8.3 Hz, 1H), 7.32 (d, *J* = 8.8 Hz, 2H), 6.97 (s, 1H), 2.21 (d, *J* = 17.0 Hz, 6H); ^13^C NMR (151 MHz, CDCl_3_) δ 168.26, 134.64, 133.88, 131.14, 129.72, 124.70, 118.08, 24.27, 17.58.

*N-(4-Hydroxyphenyl)-acetamide* (**1k**)The product was obtained as white solid in 89% yield (0.40 g); MP: 170–172 °C; ^1^H NMR (600 MHz, DMSO-*d*_6_) δ 9.65 (s, 1H), 9.14 (s, 1H), 7.34 (d, *J* = 8.8 Hz, 2H), 6.68 (d, *J* = 8.8 Hz, 2H), 1.98 (s, 3H); ^13^C NMR (151 MHz, DMSO-*d*_6_) δ 168.09, 153.43, 131.32, 121.27, 115.38, 24.09.

*N-(4-Nitro-phenyl)-acetamide* (**1l**): The product was obtained as yellow solid in 63% yield (0.34 g); MP: 209–211 °C; ^1^H NMR (600 MHz, DMSO-*d*_6_) δ 10.56 (s, 1H), 8.21 (dd, *J* = 9.1, 1.7 Hz, 2H), 7.95–7.65 (m, 2H), 2.12 (s, 3H); ^13^C NMR (151 MHz, DMSO-*d*_6_) δ 169.95, 145.66, 142.50, 125.42, 119.01, 24.56.

*N-(4-Formyl-phenyl)-acetamide* (**1m**): The product was obtained as white solid in 57% yield (0.28 g); MP: 155–157 °C; ^1^H NMR (600 MHz, CDCl_3_) δ 9.92 (s, 1H), 8.07 (s, 1H), 7.95–7.78 (m, 2H), 7.73 (d, *J* = 8.3 Hz, 2H), 2.24 (s, 3H); ^13^C NMR (151 MHz, CDCl_3_) δ 191.27, 168.97, 143.65, 132.12, 131.19, 119.20, 24.72.

*N-(4-Acetyl-phenyl)-acetamide* (**1n**): The product was obtained as white solid in 68% yield (0.36 g); MP: 166–168 °C; ^1^H NMR (600 MHz, DMSO-*d*_6_) δ 10.28 (s, 1H), 8.03–7.83 (m, 2H), 7.80–7.60 (m, 2H), 2.52 (s, 3H), 2.09 (s, 3H); ^13^C NMR (151 MHz, DMSO-*d*_6_) δ 197.15, 169.48, 143.90, 131.97, 129.93, 118.57, 26.84, 24.53.

*N-Benzylacetamide* (**1o**): The product was obtained as off-white solid in 93% yield (0.42 g); MP: 60–63 °C; ^1^H NMR (600 MHz, DMSO-*d*_6_) δ 8.48–8.24 (m, 1H), 7.40–7.28 (m, 2H), 7.28–7.17 (m, 3H), 4.26 (dd, *J* = 9.6, 4.1 Hz, 2H), 1.92–1.83 (m, 3H); ^13^C NMR (151 MHz, DMSO-*d*_6_) δ 169.95, 139.88, 128.73, 127.69, 127.21, 42.50, 22.92.

*(R)-N-methyl-N-phenylacetamide* (**1p**): The product was obtained as yellow solid in 94% yield (0.46 g); MP: 98–101 °C; ^1^H NMR (600 MHz, DMSO-*d*_6_) δ 8.29 (d, *J* = 8.1 Hz, 1H), 7.33–7.23 (m, 4H), 7.25–7.13 (m, 1H), 4.90 (dd, *J* = 7.9, 6.8 Hz, 1H), 1.84 (s, 3H), 1.32 (d, *J* = 7.1 Hz, 3H); ^13^C NMR (151 MHz, DMSO-*d*_6_) δ 168.97, 145.18, 128.69, 127.04, 126.38, 48.26, 23.06, 22.9. ${\left[\alpha \right]}_{D}^{25}$ = 124 (c = 0.5, CHCl_3_).

*N-(4-Methyl-benzothiazol-2-yl)-acetamide* (**1q**): The product was obtained as white solid in 60% yield (0.37 g); MP: 250–252 °C; ^1^H NMR (600 MHz, DMSO-*d*_6_) δ 12.44 (s, 1H), 7.77 (d, *J* = 7.8 Hz, 1H), 7.39–6.96 (m, 2H), 2.57 (s, 3H), 2.19 (s, 3H); ^13^C NMR (151 MHz, DMSO-*d*_6_) δ 169.84, 157.36, 147.97, 131.44, 130.25, 127.08, 123.95, 119.48, 23.08, 18.36.

*N-(6-Methyl-benzothiazol-2-yl)-acetamide* (**1r**): The product was obtained as white solid in 72% yield (0.44 g); MP: 216–218 °C; ^1^H NMR (600 MHz, DMSO-*d*_6_) δ 12.28 (s, 1H), 7.87–7.66 (m, 1H), 7.62 (d, *J* = 8.2 Hz, 1H), 7.24 (dd, *J* = 8.3, 1.7 Hz, 1H), 2.41 (s, 3H), 2.20 (s, 3H); ^13^C NMR (151 MHz, DMSO-*d*_6_) δ 169.78, 157.39, 146.84, 133.46, 131.95, 127.87, 121.67, 120.57, 23.13, 21.38.

*N-(6-Chloro-benzothiazol-2-yl)-acetamide* (**1s**): The product was obtained as white solid in 75% yield (0.51 g); MP: 225–227 °C; ^1^H NMR (600 MHz, DMSO-*d*_6_) δ 12.45 (s, 1H), 8.11 (d, *J* = 2.5 Hz, 1H), 7.73 (dd, *J* = 8.7, 1.5 Hz, 1H), 7.46 (s, 1H), 2.23 (s, 3H); ^13^C NMR (151 MHz, DMSO-*d*_6_) δ 170.14, 159.05, 147.75, 133.49, 128.03, 126.89, 122.13, 121.71, 23.09.

*N-[2-(4-Methoxy-phenyl)-ethyl]-acetamide* (**1t**): The product was obtained as white solid in 95% yield (0.55 g); MP: 86–87 °C; ^1^H NMR (600 MHz, DMSO-*d*_6_) δ 7.89 (s, 1H), 7.12 (d, *J* = 8.6 Hz, 2H), 6.96–6.71 (m, 2H), 3.72 (s, 3H), 3.29–3.07 (m, 2H), 2.63 (t, *J* = 7.5 Hz, 2H), 1.78 (s, 3H); ^13^C NMR (151 MHz, DMSO-*d*_6_) δ 169.97, 158.07, 131.75, 130.00, 114.17, 55.39, 40.95, 34.66, 22.97.

*N-Cyclohexylacetamide* (**1u**): The product was obtained as off-white solid in 94% yield (0.40 g); MP: 100–103 °C; ^1^H NMR (600 MHz, CDCl_3_) 5.57 (s, 1H), 4.00–3.53 (m, 1H), 1.96 (s, 5H), 1.80–1.64 (m, 2H), 1.68–1.53 (m, 1H), 1.48–1.28 (m, 2H),1.23–1.00 (m, 3H); ^13^C NMR (151 MHz, CDCl_3_) δ 169.12, 48.15, 33.15, 25.52, 24.88, 23.49.

*N-(4-Methyl-cyclohexyl)-acetamide* (**1v**): The product was obtained as white solid in 95% yield (0.44 g); MP: 139–140 °C; ^1^H NMR (600 MHz, DMSO-*d*_6_) δ 7.67 (d, *J* = 7.9 Hz, 1H), 3.42 (dtd, *J* = 11.7, 7.7, 3.9 Hz, 1H), 1.81–1.67 (m, 5H), 1.68–1.59 (m, 2H), 1.32–1.19 (m, 1H), 1.19–1.02 (m, 2H), 1.03–0.89 (m, 2H), 0.85 (d, *J* = 6.6 Hz, 3H); ^13^C NMR (151 MHz, DMSO-*d*_6_) δ 168.86, 47.99, 34.05, 32.75, 31.94, 23.06, 22.60.

*N-(6-Bromo-pyridin-2-yl)-acetamide* (**1w**): The product was obtained as off-white solid in 89% yield (0.57 g); MP: 158–159 °C; ^1^H NMR (600 MHz, CDCl_3_) δ 8.16 (d, *J* = 8.2 Hz, 1H), 8.09 (s, 1H), 7.56 (t, *J* = 7.9 Hz, 1H), 7.21 (dd, *J* = 7.7, 0.8 Hz, 1H), 2.21 (s, 3H); ^13^C NMR (151 MHz, CDCl_3_) δ 168.65, 151.28, 140.69, 139.12, 123.52, 112.27, 24.67.

*N-Phenyl-formamide* (**2a**): The product was obtained as off-white solid in 89% yield (0.34 g); MP: 48–50 °C; ^1^H NMR (600 MHz, CDCl_3_) Mixture of rotamers is observed. Ratio: 5/5. Major rotamer: δ 8.47 (s, 1H), 8.35 (d, *J* = 2.0 Hz, 1H), 7.58 (dd, *J* = 8.6, 1.0 Hz, 1H), 7.39–7.30 (m, 2H), 7.17–7.09 (m, 2H); Minor rotamer: δ 9.19 (d, *J* = 9.0 Hz, 1H), 8.72 (d, *J* = 11.4 Hz, 1H), 7.58 (dd, *J* = 8.6, 1.0 Hz, 1H), 7.39–7.30 (m, 2H,), 7.22–7.17 (m, 1H), 7.17–7.09 (m, 1H); ^13^C NMR (151 MHz, CDCl_3_) δ 162.90, 159.39, 136.88, 125.25, 120.00, 118.69, 77.08.

*N-(4-Chloro-phenyl)-formamide* (**2b**): The product was obtained as yellow brown solid in 85% yield (0.40 g); MP: 104–106 °C; ^1^H NMR (600 MHz, DMSO-*d*_6_), Mixture of rotamers is observed. Ratio: 5.9/4.1; Major rotamer: δ 8.31 (d, *J* = 1.2 Hz, 1H), 8.23 (s, 1H), 7.65 (m, 2H), 7.39–7.23 (m, 2H); Minor rotamer: δ 8.82 (d, *J* = 11.6 Hz, 1H), 7.38 (s, 1H), 7.39–7.23 (m, 2H) 7.21 (m, 2H); ^13^C NMR (151 MHz, DMSO-*d*_6_) δ 162.89, 160.18, 160.11, 160.09, 137.45, 137.43, 129.67, 129.21, 129.20, 127.69, 121.19, 121.11, 119.47, 119.38.

*N-(3-Chloro-phenyl)-formamide* (**2c**): The product was obtained as cream white solid in 89% yield (0.42 g); MP: 49–50°C; ^1^H NMR (600 MHz, DMSO-*d*_6_) δ 10.36 (t, *J* = 4.1 Hz, 1H), 8.62 (t, *J* = 2.0 Hz, 1H), 8.47–8.20 (m, 2H), 2.21–1.98 (m, 3H); ^13^C NMR (151 MHz, DMSO-*d*_6_) δ 169.85, 144.63, 144.60, 139.25, 137.24, 128.18, 128.09, 120.09, 24.25.

*N-(3-Nitro-phenyl)-formamide* (**2d**): The product was obtained as yellow solid in 71% yield (0.35 g); MP: 138–139 °C; ^1^H NMR (600 MHz, DMSO-*d*_6_), Mixture of rotamers is observed. Ratio: 7.1/2.9 Major rotamer: δ 8.63 (s, 1H), 8.38 (t, *J* = 2.0 Hz, 1H), 7.96–7.89 (m, 2H), 7.89–7.88 (m, 2H); (Minor rotamer: δ 8.96 (d, *J* = 10.4 Hz,1H), 7.97 (d, *J* = 1.2 Hz, 1H), 7.89–7.88 (m, 1H), 7.87 (dd, *J* = 8.0 and 1.6 Hz, 1H), 7.66–7.63 (m, 1H); ^13^C NMR (151 MHz, DMSO-*d*_6_) δ 160.85, 148.39, 139.40, 130.88, 125.58, 118.80, 113.77.

*N-Benzyl-formamide* (**2e**): The product was obtained as white solid in 92% yield (0.37 g); MP: 61–62 °C; ^1^H NMR (600 MHz, DMSO-*d*_6_) δ 8.53 (s, 1H), 8.23–8.08 (m, 1H), 7.40–7.19 (m, 5H), 4.34 (s, 2H); ^13^C NMR (151 MHz, DMSO-*d*_6_) δ 161.64, 139.28, 128.82, 127.73, 127.39, 41.12.

*N-p-Tolyl-benzamide* (**3a**): The product was obtained as gray solid in 89% yield (0.57 g); MP: 156–158 °C; ^1^H NMR (600 MHz, CDCl_3_) δ 7.85 (dt, *J* = 7.1, 1.4 Hz, 3H), 7.55–7.42 (m, 5H), 7.16 (d, *J* = 8.2 Hz, 2H), 2.34 (s, 3H); ^13^C NMR (151 MHz, CDCl_3_) δ 165.64, 135.27, 135.05, 134.24, 131.74, 129.58, 128.75 , 127.00, 120.35, 120.23, 20.93.

*(R)-N-(1-Phenyl-ethyl)-benzamide* (**3b**): The product was obtained as white solid in 94% yield (0.64 g); MP: 137–138 °C; 1H NMR (600 MHz, CDCl_3_) δ 7.80–7.74 (m, 2H), 7.52–7.47 (m, 1H), 7.45–7.34 (m, 5H), 7.31–7.27 (m, 1H), 5.34 (q, *J* = 6.9 Hz, 1H), 1.61 (d, *J* = 6.9 Hz, 3H); ^13^C NMR (151 MHz, CDCl_3_) δ 166.54, 143.06, 134.54, 131.51, 128.77, 128.58, 127.50, 126.90, 126.26, 49.13, 21.71. ${\left[\alpha \right]}_{D}^{25}$ = +19.9 (c = 1.0, CHCl_3_).

*N-[2-(4-Methoxy-phenyl)-ethyl]-benzamide* (**3c**): The product was obtained as white solid in 97% yield (0.74 g); MP: 120–121 °C; ^1^H NMR (600 MHz, CDCl_3_) δ 7.75–7.62 (m, 2H), 7.52–7.44 (m, 1H), 7.40 (dd, *J* = 8.4, 7.0 Hz, 2H), 7.15 (d, *J* = 8.5 Hz, 2H), 6.87 (d, *J* = 8.6 Hz, 2H), 3.80 (s, 3H), 3.68 (td, *J* = 6.9, 5.8 Hz, 2H), 2.87 (t, *J* = 6.9 Hz, 2H); ^13^C NMR (151 MHz, CDCl_3_) δ 167.43, 158.31, 134.62, 131.39, 130.85, 129.75, 128.55, 126.79, 114.12, 55.28, 41.19, 34.75.

*N-Cyclohexyl-benzamide* (**3d**)**:** The product was obtained as white solid in 93% yield (0.57 g); MP: 146–147 °C; ^1^H NMR (600 MHz, CDCl_3_) δ 7.87–7.64 (m, 2H), 7.54–7.34 (m, 3H), 5.99 (s, 1H), 3.98 (dt, *J* = 6.8, 2.8 Hz, 1H), 2.03 (dt, *J* = 12.5, 3.9 Hz, 2H), 1.91–1.55 (m, 4H), 1.53–1.33 (m, 2H), 1.33–1.09 (m, 3H); ^13^C NMR (151 MHz, CDCl_3_) δ 166.60,135.06, 131.25, 128.52, 126.80, 48.56, 33.21, 25.58, 24.91.

Experimental procedures and analytical data of all compounds (^1^H NMR and ^13^C NMR), copy of the ^1^H NMR, ^13^C NMR and data are available in the Supplementary Materials.

## 4. Conclusions

In conclusion, a simple method for the transamidation process of primary amines taking imidazolium chloride as the only promoter is reported. This method has wide substrate scope and provides moderate to good yields. Moreover, considering the advantages of imidazolium chloride, including being inexpensive, readily available, and environmentally-friendly, this simple procedure is a valuable addition to the arsenal of amide syntheses.

## Figures and Tables

**Table 1:** Optimization studies for transamidation ^a^.

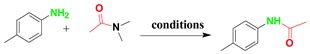					
Entry	Base (equiv.)	Solvent (mL)	Temp (°C)	Time (h)	Yield ^b^ (%)
1	CuCl_2_	DMA	150	12	Trace
2	Zr(SO_4_)_2_	DMA	150	12	Trace
3	CH3COOH(2)	DMA	150	6	8
4	HCl(2)	DMA	150	6	46
5	H_2_SO_4_(2)	DMA	150	6	52
6	*p*-toluenesulfonic acid	DMA	150	6	50
7	Pyridine(3)	DMA	150	6	Trace
8	KOtBu(2)	DMA	150	6	12
9	NaOH(2)	DMA	150	6	18
10	2,6-lutidine(3)	DMA	150	6	Trace
11	TEA(3)	DMA	150	6	Trace
12	Imidazolium chloride(3)	DMA	rt	6	Trace
13	Imidazolium chloride(3)	DMA	150	2	96
14	Imidazolium chloride(0.5)	DMA	150	2	95
15	Imidazolium chloride(0.3)	DMA	150	2	96
16	Imidazolium chloride(0.1)	DMA	150	6	65
17	Imidazolium chloride(3)	DMA	30	2	Trace
18	Imidazolium chloride(3)	DMA	60	2	59
19	Imidazolium chloride(3)	DMA	90	2	68
20	Imidazolium chloride(3)	DMA	135	2	85
21	Imidazole(3)	DMA	150	12	Trace
22	-	DMA	150	12	NO^C^

^a^ Reaction conditions: amine (0.3 g, 2.8 mmol, 1 equiv.), solvent (5.0 mL), temperature 60–150 °C, all reagent and substrate addition was done at room temperature (25 °C), ^b^ isolated yield; ^c^ no reaction.

**Table 2:** Synthesis of benzamides via transamidation ^a^.

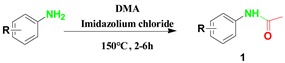				
Entry	Substrate	Time	Product	Yield ^b^ (%)
1	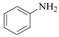	2 h	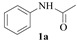	92
2	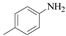	2 h	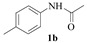	97
3	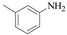	3 h	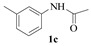	90
4	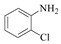	2 h	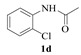	75
5	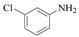	2 h	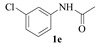	82
6	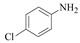	4 h	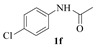	84
7	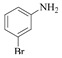	6 h	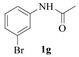	83
8	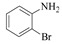	6 h	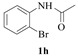	80
9	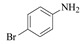	5 h	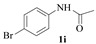	89
10	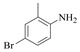	6 h	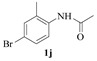	90
11	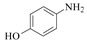	4 h	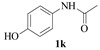	89
12	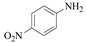	4 h	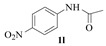	63
13	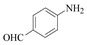	5 h	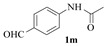	57
14	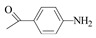	4 h	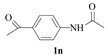	68
15	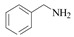	3 h	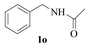	93
16	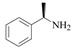	3 h	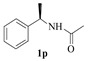	94
17	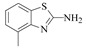	5 h	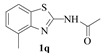	60
18	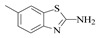	5 h	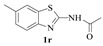	72
19	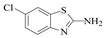	5 h	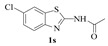	75
20	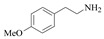	3 h	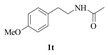	95
21	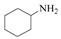	3 h	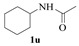	94
22	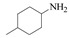	3 h	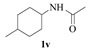	95
23	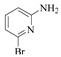	3 h	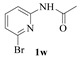	89

^a^ Conditions unless otherwise stated: amine (1.0 equiv.), DMA (1.5 mL), Imidazolium chloride (0.3 equiv.) in a sealed tube at 150 °C for 2–8 h. ^b^ isolated yield.

**Table 3:** *N*-benzamides of amines **2a**–**2e**^a^.

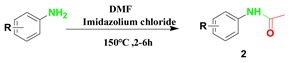				
Entry	Substrate	Time	Product	Yield ^b^ (%)
1	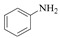	4 h	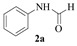	89
2	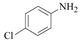	4 h	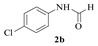	85
3	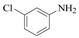	4 h	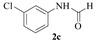	89
4	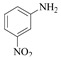	4 h	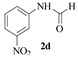	71
5	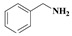	3 h	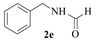	92

^a^ Conditions unless otherwise stated: amine (1.0 equiv.), DMF (1.5 mL), Imidazolium chloride (0.3 equiv.) in a sealed tube at 150 °C for 2–8 h. ^b^ isolated yield.

**Table 4:** *N*-benzoylation of amines **3a**–**3d**^a^.

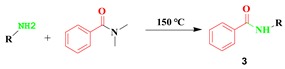				
Entry	Substrate	Time	Product	Yield ^b^ (%)
1	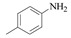	4 h	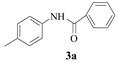	89
2	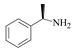	3 h	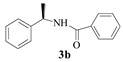	94
3	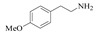	3 h	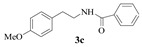	97
4	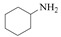	3 h	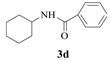	93

^a^ Conditions unless otherwise stated: amine (1.0 equiv.), *N*,*N*-Dimethylbenzamide (2.0 equiv.), Imidazolium chloride (0.3 equiv.) in a sealed tube at 150 °C for 2–4 h. ^b^ isolated yield.

## Supplementary Materials

The following are available online, general procedure for Transamidation of primary amines reaction and ^1^H NMR and ^13^C NMR spectra of all products.
