# Supplementary material for: Imidazolium Chloride: An Efficient Catalyst for Transamidation of Primary Amines
Source: Molecules. 2018 Sep 2;23(9):2234. doi: 10.3390/molecules23092234 (PMC6225136; doi:10.3390/molecules23092234)
Supplement: Supplementary file 1 [file molecules-23-02234-s001.pdf]

Supporting Information

Imidazolium Chloride: An Efficient Catalyst for Transamidation of Primary Amines

Qingqiang Tian, Zongjie Gan, Xuetong Wang, Dan Li, Wen Luo, Huajun Wang, Zeshu Dai and Jianyong Yuan*

Pharmacy of College, Chongqing Medical University, Chongqing 400016, China

* Corresponding author;

E-mail address: enediyne@163.com; Tel. /Fax: +86-23-6848-5161

Table of contents

1. General information 2

2. General Procedures 2

3. Characterization of products N-Acetamides, N-Formamides and N-Benzoylation..4

4. Synthesis and characterization of 1-Acetylimidazole 16

5. References 16

6. [1] H NMR and [13] C NMR spectra of N-Acetamides, N-Formamides and N- Benzoylation..19

1. General information

All reactions were carried out under normal conditions and no any stringent conditions were used. All reagents were obtained from Aladdin Reagent Shanghai Co., Ltd., Lagewell Technology Co., Ltd., Meyer Reagent Shanghai Co., Ltd., Macklin Reagent Shanghai Co., Ltd., Chongqing Chuandong Chemical Co., Ltd etc. without further purification unless otherwise noted. Reactions were monitored by TLC analysis using Merck Silica Gel 60 F-254 thin layer plates. The plates were visualized first with short wavelength UV light followed by iodine stain.

^{1}H and ^{13}C NMR spectra were recorded in $CDCl_3$ and DMSO-d6 on a Bruker Ascend-III 600 MHz and 600 MHz spectrometer using TMS as an internal standard. The residual solvent signals were used as references and the chemical shifts converted to the TMS scale ($CDCl_3$: δ H = 7.25-7.26 ppm, δ C = 77.23 ppm; DMSO-d 6 : δ H = 2.51 ppm, δ C = 39.51 ppm).

2. General Procedures

2.1 Synthesis of N-Acetamides:

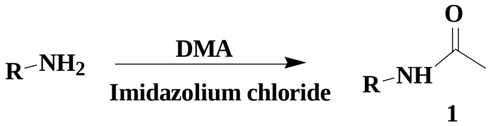

1

To a mixture of aromatic or aliphatic or heterocyclic amine (3.0 mmol, 1.0 equiv), Imidazolium chloride (1.0 mmol, 0.3 equiv), N, N-dimethyl acetamide (2.0 mL) was added. The mixture was refluxed at 150 ℃ and the progress of the reaction was monitored by TLC visualized with UV short wavelength followed by iodine stain. After completion, the mixture was diluted with cold water (10 mL) then extracted with EtOAc (10 mL). The EtOAc layer was washed with 1M hydrochloric acid (3.0 X 15 mL).

Adsorption of pigment with activated carbon, filtration of filtrate.The filtrate was dried over anhydrous Na_2SO_4 and concentrated under vacuum to obtain crude N-acetamide amine, the N-acetamide amine product was isolated by column chromatography eluting with Petroleum ether: ethyl acetate (10:1) mixtures.

2.2 Synthesis of N-Formamides:

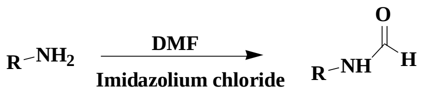

2

To a mixture of aromatic or aliphatic or heterocyclic amine (3.0 mmol, 1.0 equiv), Imidazolium chloride (1.0 mmol, 0.3 equiv), N, N-dimethyl formamide (2.0 mL) was added. The mixture was refluxed at 150 ℃ and the progress of the reaction was monitored by TLC visualized with UV short wavelength followed by iodine stain. After completion, the mixture was diluted with cold water (10 mL) then extracted with EtOAc (10 mL). The EtOAc layer was washed with 1M hydrochloric acid (3.0 x 15 mL). Adsorption of pigment with activated carbon, filtration of filtrate.The filtrate was dried over anhydrous Na_2SO_4 and concentrated under vacuum to obtain crude N-formyl amine, the N-formyl amine product was isolated by column chromatography eluting with Petroleum ether: ethyl acetate (10:1) mixtures.

2.3 **Synthesis of N- Benzoylation:**

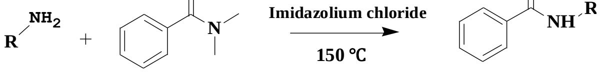

3

To a mixture of aromatic or aliphatic or heterocyclic amine (3.0 mmol, 1.0 equiv), Imidazolium chloride (1.0 mmol, 0.3 equiv), N, N-

dimethylbenzamide (2.0 equiv) was added. The mixture was refluxed at 150 ℃ and the progress of the reaction was monitored by TLC visualized with UV short wavelength followed by iodine stain. After completion, the mixture was diluted with cold water (10 mL). The crystallized solid were filtered and washed with water and heptane, dried under vacuum to give the product.

3. Characterization of products N-Acetamides, N-Formamides and N- Benzoylation

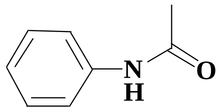

1a

N-phenylacetamide (1a)[1]: The product was obtained as off-white solid in 92% yield (0.37 g); MP:112-115 ℃; ^{1}H NMR (600 MHz, $CDCl_3$) δ 7.82 (s, 1H), 7.54 – 7.47 (m, 2H), 7.29 (dd, J = 8.5, 7.4 Hz, 2H), 7.12 – 7.07 (m, 1H), 2.15 (s, 3H); ^{13}C NMR (151 MHz, $CDCl_3$) δ 168.74 , 137.90 , 128.94 , 124.29 , 119.97 , 24.43 .

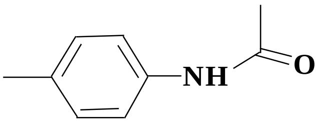

1b

N-p-tolylacetamide (1b)[2]: The product was obtained as pale yellow solid in 97% yield (0.43 g); MP:150-153 ℃; ^{1}H NMR (600 MHz, DMSO-d_6) δ 9.83 (s, 1H), 7.46 (dd, J = 8.7, 2.4 Hz, 2H), 7.08 (d, J = 8.3 Hz, 2H), 2.24 (s, 3H), 2.02 (s, 3H); ^{13}C NMR (151 MHz, DMSO-d_6) δ 168.93 , 136.84 , 132.67 , 129.52 , 119.56 , 24.16 , 20.81 .

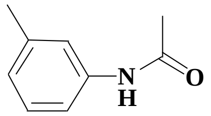

1c

N-m-Tolyl-acetamide (1c) [3]**:** The product was obtained as dark brown solid in 90% yield (0.40 g); MP: 66 - 68 ℃; ^{1}H NMR (600 MHz, DMSO-d_6) δ 9.91 (s, 1H), 7.43 (d, *J* = 2.0 Hz, 1H), 7.40 – 7.35 (m, 1H), 7.18 (t, *J* = 7.8 Hz, 1H), 6.89 – 6.84 (m, 1H), 2.27 (s, 3H), 2.05 (s, 3H); ^{13}C NMR (151 MHz, DMSO-d_6) δ 168.82 , 139.47 , 138.30 , 128.94 , 124.24 , 119.96 , 116.63 , 24.32 , 21.60 .

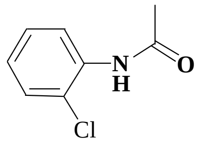

1d

N-(2-Chloro-phenyl)-acetamide (1d) [7]: The product was obtained as off-white solid in 75% yield (0.38 g); MP: 87 - 88 ℃; ^{1}H NMR (600 MHz, $CDCl_3$) δ 8.35 (d, J = 8.4 Hz, 1H), 7.64 (s, 1H), 7.36 (d, J = 8.0 Hz, 1H), 7.27 (t, J = 7.8 Hz, 1H), 7.03 (t, J = 7.7 Hz, 1H), 2.24 (s, 3H); ^{13}C NMR (151 MHz, $CDCl_3$) δ 168.26 , 134.61 , 128.96 , 127.73 , 124.62 , 122.55 , 121.66 , 24.88.

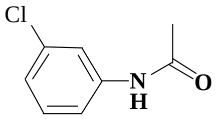

1e

N-(3-chlorophenyl)acetamide (1e) [1]**:** The product was obtained as pale yellowish-brown solid in 82% yield (0.42 g); MP: 74-77 ℃; ^{1}H NMR (600 MHz, DMSO-d_6) δ 10.13 (s, 1H), 7.94 – 7.68 (m, 1H), 7.57 – 7.35

(m, 1H), 7.36 – 7.27 (m, 1H), 7.14 – 7.02 (m, 1H), 2.06 (d, J = 0.8 Hz, 3H); ^{13}C NMR (151 MHz, DMSO-d_6) δ 169.17 , 141.01 , 133.50 , 130.80 , 123.17 , 118.80 , 117.67 , 24.39 .

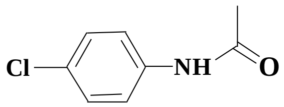

1f

N-(4-chlorophenyl)acetamide (1f) [1] : The product was obtained as pale brown solid in 84% yield (0.43 g); MP:175-177 ℃; ^{1}H NMR (600 MHz, DMSO-d6) δ 10.07 (s, 1H), 7.71 – 7.48 (m, 2H), 7.48 – 7.23 (m, 2H), 2.04 (d, J = 1.3 Hz, 3H); ^{13}C NMR (151 MHz, DMSO-d_6) δ 169.00 , 138.48 , 138.46 , 129.01 , 127.06 , 120.93 , 24.31 .

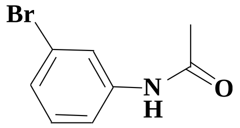

1g

N-(3-Bromo-phenyl)-acetamide (1g) [8] **:** The product was obtained as pale brown solid in 83% yield (0.53 g); MP: 85 - 87 ℃; ^{1}H NMR (600 MHz, DMSO-d_6) δ 10.11 (s, 1H), 7.95 (d, J = 1.9 Hz, 1H), 7.47 – 7.44 (m, 1H), 7.26 (t, J = 8.0 Hz, 1H), 7.24 – 7.19 (m, 1H), 2.05 (s, 3H); ^{13}C NMR (151 MHz, DMSO-d_6) δ 169.24, 141.11, 131.17 , 126.12 , 121.98 , 121.65 , 118.09 , 24.38 .

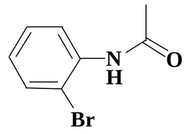

1h

N-(2-Bromo-phenyl)-acetamide (1h) [9]**:** The product was obtained as pale brown solid in 80 % yield (0.51 g); MP: 97 - 99 ℃ ; ^{1}H NMR (600 MHz, $CDCl_3$) δ 8.33 (d, J = 8.3 Hz, 1H), 7.61 (s, 1H), 7.53 (dd, J = 8.0, 1.4 Hz, 1H), 7.31 (ddd, J = 8.5, 7.4, 1.5 Hz, 1H), 7.01 – 6.92 (m, 1H), 2.24 (s, 3H); ^{13}C NMR (151 MHz, $CDCl_3$) δ 168.17 , 135.59 , 132.19 , 128.39 , 125.16 , 121.87 , 113.10 , 24.83 .

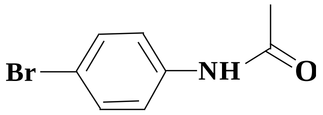

1i

N-(4-bromophenyl)acetamide (1i) [2] **:**The product was obtained as pale yellowish-brown solid in 89% yield (0.57 g); MP: 166-169 ℃; ^{1}H NMR (600 MHz, DMSO-d6) δ 10.07 (s, 1H), 7.56 (d, J = 8.8 Hz, 2H), 7.47 (d, J = 8.8 Hz, 2H), 2.05 (s, 3H); ^{13}C NMR (151 MHz, DMSO-d6) δ 169.12 , 138.83 , 131.91 , 121.36 , 115.10 , 24.31 .

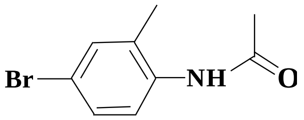

1j

N-(4-Bromo-2-methyl-phenyl)-acetamide (1j) [10]**:** The product was obtained as off-white solid in 90% yield (0.61 g); MP:160-162 ℃; ^{1}H NMR (600 MHz, $CDCl_3$) δ 7.68 (d, J = 8.3 Hz, 1H), 7.32 (d, J = 8.8 Hz, 2H), 6.97 (s, 1H), 2.21 (d, J = 17.0 Hz, 6H); ^{13}C NMR (151 MHz, $CDCl_3$) δ 168.26 , 134.64 , 133.88 , 131.14 , 129.72 , 124.70 , 118.08 , 24.27 , 17.58 .

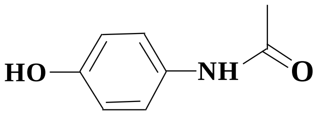

1k

N-(4-hydroxyphenyl)acetamide (1k) [3]**:** The product was obtained as white solid in 89% yield (0.40 g); MP: 170-172 ℃; ^{1}H NMR (600 MHz, DMSO-d_6) δ 9.65 (s, 1H), 9.14 (s, 1H), 7.34 (d, J = 8.8 Hz, 2H), 6.68 (d, J = 8.8 Hz, 2H), 1.98 (s, 3H); ^{13}C NMR (151 MHz, DMSO-d_6) δ 168.09 , 153.43 , 131.32 , 121.27 , 115.38 , 24.09 .

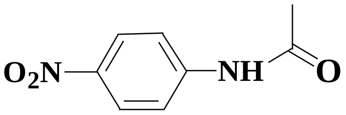

1l

N-(4-Nitro-phenyl)-acetamide (1l) [11]: The product was obtained as yellow solid in 63% yield (0.34 g); MP: 209-211 ℃; ^{1}H NMR (600 MHz, DMSO-d_6) δ 10.56 (s, 1H), 8.21 (dd, J = 9.1, 1.7 Hz, 2H), 7.95 – 7.65 (m, 2H), 2.12 (s, 3H); ^{13}C NMR (151 MHz, DMSO-d_6) δ 169.95 , 145.66 , 142.50 , 125.42 , 119.01 , 24.56 .

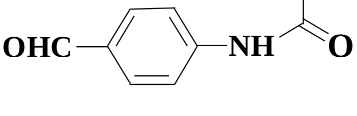

1m

N-(4-Formyl-phenyl)-acetamide (1m) [12]**:** The product was obtained as white solid in 57% yield (0.28 g); MP: 155-157 ℃; 1H NMR (600 MHz, $CDCl_3$) δ 9.92 (s, 1H), 8.07 (s, 1H), 7.95 – 7.78 (m, 2H), 7.73 (d, J = 8.3

Hz, 2H), 2.24 (s, 3H).; 13C NMR (151 MHz, $CDCl_3$) δ 191.27 , 168.97 , 143.65 , 132.12 , 131.19 , 119.20 , 24.72 .

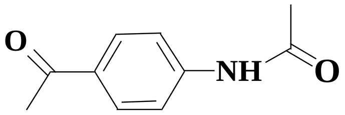

1n

N-(4-Acetyl-phenyl)-acetamide (1n) [14]**:** The product was obtained as white solid in 68% yield (0.36 g); MP: 166 - 168 ℃; ^{1}H NMR (600 MHz, DMSO-d_6) δ 10.28 (s, 1H), 8.03 – 7.83 (m, 2H), 7.80 – 7.60 (m, 2H), 2.52 (s, 3H), 2.09 (s, 3H); ^{13}C NMR (151 MHz, DMSO-d_6) δ 197.15 , 169.48 , 143.90 , 131.97 , 129.93 , 118.57 , 26.84 , 24.53 .

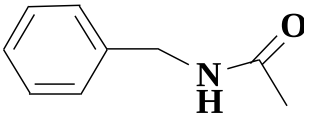

1o

N-benzylacetamide (1o) [6]**:** The product was obtained as off-white solid in 93% yield (0.42 g); MP: 60-63 ℃; ^{1}H NMR (600 MHz, DMSO-d_6) δδ 8.48 – 8.24 (m, 1H), 7.40 – 7.28 (m, 2H), 7.28 – 7.17 (m, 3H), 4.26 (dd, *J* = 9.6, 4.1 Hz, 2H), 1.92 – 1.83 (m, 3H); ^{13}C NMR (151 MHz, DMSO-d6) δ 169.95 , 139.88 , 128.73 , 127.69 , 127.21 , 42.50 , 22.92 .

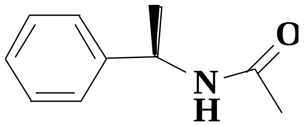

1p

N-methyl-N-phenylacetamide (1p) [4]**:** The product was obtained as yellow solid in 94% yield (0.46 g); MP: 98-101 ℃; ^{1}H NMR (600 MHz, DMSO-d6) δ 8.29 (d, J = 8.1 Hz, 1H), 7.33 – 7.23 (m, 4H), 7.25 – 7.13 (m, 1H), 4.90 (dd, J = 7.9, 6.8 Hz, 1H), 1.84 (s, 3H), 1.32 (d, J = 7.1 Hz, 3H); ^{13}C NMR (151 MHz, DMSO-d6) δ 168.97 , 145.18 , 128.69 , 127.04 , 126.38 , 48.26 , 23.06 , 22.9. $[\alpha]_D^{25}$ =124 (c = 0.5, $CHCl_3$).

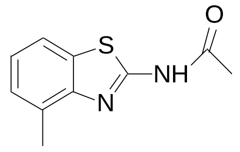

1q

N-(4-Methyl-benzothiazol-2-yl)-acetamide (1q) [15]**:** The product was obtained as white solid in 60% yield (0.37 g); MP: 250 - 252 ℃; ^{1}H NMR (600 MHz, DMSO-d6) δ 12.44 (s, 1H), 7.77 (d, J = 7.8 Hz, 1H), 7.39 – 6.96 (m, 2H), 2.57 (s, 3H), 2.19 (s, 3H); ^{13}C NMR (151 MHz, DMSO-d6) δ 169.84 , 157.36 , 147.97 , 131.44 , 130.25 , 127.08 , 123.95 , 119.48 , 23.08 , 18.36 .

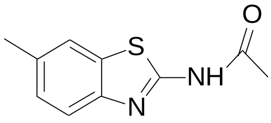

1r

N-(6-Methyl-benzothiazol-2-yl)-acetamide (1r) [16]**:** The product was obtained as white solid in 72% yield (0.44 g); MP: 216 - 218 ℃; ^{1}H NMR (600 MHz, DMSO-d_6) δ 12.28 (s, 1H), 7.87 – 7.66 (m, 1H), 7.62 (d, J = 8.2 Hz, 1H), 7.24 (dd, J = 8.3, 1.7 Hz, 1H), 2.41 (s, 3H), 2.20 (s, 3H); ^{13}C NMR (151 MHz, DMSO-d_6) δ 169.78 , 157.39 , 146.84 , 133.46 , 131.95 , 127.87 , 121.67 , 120.57 , 23.13 , 21.38 .

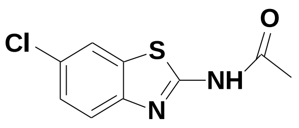

1s

N-(6-Chloro-benzothiazol-2-yl)-acetamide (1s) [16]**:** The product was obtained as white solid in 75% yield (0.51 g); MP: 225 - 227 ℃; ^{1}H NMR (600 MHz, DMSO-d6) δ 12.45 (s, 1H), 8.11 (d, J = 2.5 Hz, 1H), 7.73 (dd, J = 8.7, 1.5 Hz, 1H), 7.46 (s, 1H), 2.23 (s, 3H); ^{13}C NMR (151 MHz, DMSO-d6) δ 170.14 , 159.05 , 147.75 , 133.49 , 128.03 , 126.89 , 122.13 , 121.71 , 23.09 .

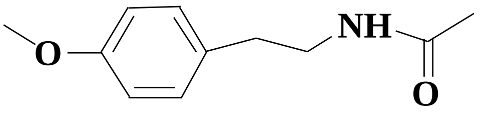

1t

N-[2-(4-Methoxy-phenyl)-ethyl]-acetamide (1t) [17]: The product was obtained as white solid in 95% yield (0.55 g); MP: 86 - 87 ℃; ^{1}H NMR (600 MHz, DMSO-d6) δ 7.89 (s, 1H), 7.12 (d, J = 8.6 Hz, 2H), 6.96 – 6.71 (m, 2H), 3.72 (s, 3H), 3.29 – 3.07 (m, 2H), 2.63 (t, J = 7.5 Hz, 2H), 1.78 (s, 3H).; ^{13}C NMR (151 MHz, DMSO-d6) δ 169.97 , 158.07 , 131.75 , 130.00 , 114.17 , 55.39 , 40.95 , 34.66 , 22.97 .

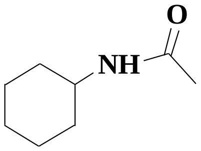

1u

N-cyclohexylacetamide (1u) [5] : The product was obtained as off-white solid in 94% yield (0.40 g); MP: 100-103 ℃; ^{1}H NMR (600 MHz, $CDCl_3$) 5.57 (s, 1H), 4.00 – 3.53 (m, 1H), 1.96 (s, 5H), 1.80 – 1.64 (m, 2H), 1.68 – 1.53 (m, 1H), 1.48 – 1.28 (m, 2H),1.23 – 1.00 (m, 3H); ^{13}C NMR (151 MHz, $CDCl_3$) δ 169.12 , 48.15 , 33.15 , 25.52 , 24.88 , 23.49.

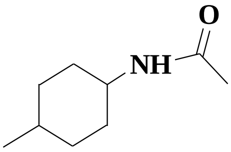

1v

N-(4-Methyl-cyclohexyl)-acetamide (1v) [18]**:** The product was obtained as white solid in 95% yield (0.44 g); MP: 139 - 140 ℃; ^{1}H NMR (600 MHz, DMSO-d6) δ 7.67 (d, J = 7.9 Hz, 1H), 3.42 (dtd, J = 11.7, 7.7, 3.9 Hz, 1H), 1.81 – 1.67 (m, 5H), 1.68 – 1.59 (m, 2H), 1.32 – 1.19 (m, 1H), 1.19 – 1.02 (m, 2H), 1.03 – 0.89 (m, 2H), 0.85 (d, J = 6.6 Hz, 3H); ^{13}C NMR (151 MHz, DMSO-d6) δ 168.86 , 47.99 , 34.05 , 32.75 , 31.94 , 23.06 , 22.60 .

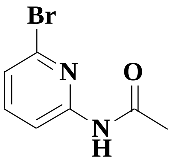

1w

N-(6-Bromo-pyridin-2-yl)-acetamide (1w) [13]: The product was obtained as off-white solid in 89% yield (0.57 g); MP: 158-159 ℃; ^{1}H NMR (600 MHz, $CDCl_3$) δ 8.16 (d, *J* = 8.2 Hz, 1H), 8.09 (s, 1H), 7.56 (t, *J* = 7.9 Hz, 1H), 7.21 (dd, *J* = 7.7, 0.8 Hz, 1H), 2.21 (s, 3H); ^{13}C NMR (151 MHz, $CDCl_3$) δ 168.65 , 151.28 , 140.69 , 139.12 , 123.52 , 112.27 , 24.67 .

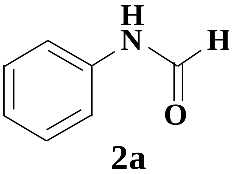

2a

N-Phenyl-formamide (2a) [19]**:** The product was obtained as off-white solid in 89% yield (0.34 g); MP: 48-50 ℃; ^{1}H NMR (600 MHz, $CDCl_3$) Mixture of rotamers is observed. Ratio: 5/5. Major rotamer: δ 8.47 (s, 1H), 8.35 (d, J = 2.0 Hz, 1H), 7.58 (dd, J = 8.6, 1.0 Hz, 1H), 7.39 –7.30 (m, 2H), 7.17 – 7.09 (m, 2H); Minor rotamer: δ 9.19 (d, J = 9.0 Hz, 1H), 8.72 (d, J = 11.4

Hz, 1H), 7.58 (dd, J = 8.6, 1.0 Hz, 1H,), 7.39 – 7.30 (m, 2H,), 7.22 – 7.17 (m, 1H), 7.17 – 7.09 (m, 1H); ^{13}C NMR (151 MHz, $CDCl_3$) δ 162.90 , 159.39 , 136.88 , 125.25 , 120.00 , 118.69 , 77.08 .

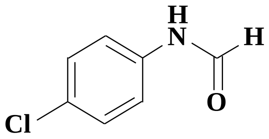

2b

N-(2-Chloro-phenyl)-formamide (2b) [20]: The product was obtained as yellow brown solid in 85% yield (0.40 g); MP: 104-106 ℃; ^{1}H NMR (600 MHz, DMSO-d_6) Mixture of rotamers is observed. Ratio: 5.9/4.1; Major rotamer: δ 8.31 (d, J = 1.2 Hz, 1H), 8.23 (s, 1H), 7.65 (m, 2H), 7.39-7.23 (m, 2H,); Minor rotamer: δ 8.82 (d, J = 11.6 Hz, 1H), 7.38 (s, 1H), 7.39-7.23 (m, 2H) 7.21 (m, 2H); ^{13}C NMR (151 MHz, DMSO-d_6) δ 162.89 , 160.18 , 160.11 , 160.09 , 137.45 , 137.43 , 129.67 , 129.21 , 129.20 , 127.69 , 121.19 , 121.11 , 119.47 , 119.38 .

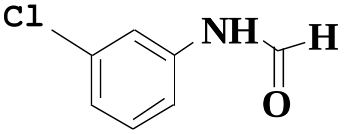

2c

N-(3-Chloro-phenyl)-formamide (2c) [21] **:** The product was obtained as cream white solid in 89% yield (0.42 g); MP: 49-50 ℃; ^{1}H NMR (600 MHz, DMSO-d_6) δ 10.36 (t, J = 4.1 Hz, 1H), 8.62 (t, J = 2.0 Hz, 1H), 8.47 – 8.20 (m, 2H), 2.21 – 1.98 (m, 3H); ^{13}C NMR (151 MHz, DMSO-d_6) δ 169.85 , 144.63 , 144.60 , 139.25 , 137.24 , 128.18 , 128.09 , 120.09 , 24.25 .

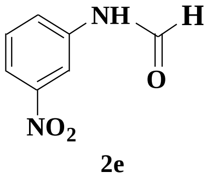

2e

N-(3-Nitro-phenyl)-formamide (2d) [19]**:** The product was obtained as yellow solid in 71% yield (0.35 g); MP: 138-139 ℃; ^{1}H NMR (600 MHz, DMSO-d6) Mixture of rotamers is observed. Ratio: 7.1/2.9 Major rotamer: δ 8.63 (s, 1H), 8.38 (t, J = 2.0 Hz, 1H), 7.96-7.89 (m, 2H), 7.89-7.88 (m, 2H); (Minor rotamer: δ 8.96 (d, J = 10.4 Hz,1H), 7.97 (d, J = 1.2 Hz, 1H), 7.89-7.88 (m, 1H), 7.87 (dd, J = 8.0 and 1.6 Hz, 1H), 7.66-7.63 (m, 1H); ^{13}C NMR (151 MHz, DMSO-d6) δ 160.85 , 148.39 , 139.40 , 130.88 , 125.58 , 118.80 , 113.77 .

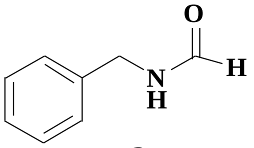

2e

N-Benzyl-formamide (2e) [19]: The product was obtained as white solid in 92% yield (0.37 g); MP: 61-62 ℃**;** ^{1}H NMR (600 MHz, DMSO-d_6) δ 8.53 (s, 1H), 8.23 – 8.08 (m, 1H), 7.40 – 7.19 (m, 5H), 4.34 (s, 2H); ^{13}C NMR (151 MHz, DMSO-d_6) δ 161.64 , 139.28 , 128.82 , 127.73 , 127.39 , 41.12 .

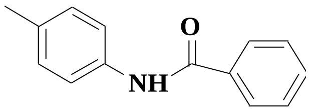

3a

N-p-Tolyl-benzamide (3a) [22]**:** The product was obtained as gray solid in 89% yield (0.57 g); MP: 156-158 ℃; ^{1}H NMR (600 MHz, $CDCl_3$) δ 7.85 (dt, J = 7.1, 1.4 Hz, 3H), 7.55 – 7.42 (m, 5H), 7.16 (d, J = 8.2 Hz, 2H), 2.34 (s, 3H); ^{13}C NMR (151 MHz, $CDCl_3$) δ 165.64 , 135.27 , 135.05 , 134.24 , 131.74 , 129.58 , 128.75 , 127.00 , 120.35 , 120.23 , 20.93 .

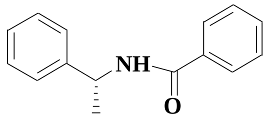

3b

N-(1-Phenyl-ethyl)-benzamide (3b) [23]**:** The product was obtained as white solid in 94% yield (0.64 g); MP: 137 - 138 ℃**;** ^{1}H NMR (600 MHz, $CDCl_3$) δ 7.80 – 7.74 (m, 2H), 7.52 – 7.47 (m, 1H), 7.45 – 7.34 (m, 5H), 7.31 – 7.27 (m, 1H), 5.34 (q, J = 6.9 Hz, 1H), 1.61 (d, J = 6.9 Hz, 3H); ^{13}C NMR (151 MHz, $CDCl_3$) δ 166.54 , 143.06 , 134.54 , 131.51 , 128.77 , 128.58 , 127.50 , 126.90 , 126.26 , 49.13 , 21.71 . $[\alpha]_D^{25}$ =+19.9 (c = 1.0, $CHCl_3$).

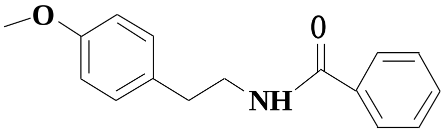

3c

N-[2-(4-Methoxy-phenyl)-ethyl]-benzamide (3c) [24]**:** The product was obtained as white solid in 97% yield (0.74 g); MP: 120-121 ℃**;** ^{1}H NMR (600 MHz, $CDCl_3$) δ 7.75 – 7.62 (m, 2H), 7.52 – 7.44 (m, 1H), 7.40 (dd, J = 8.4, 7.0 Hz, 2H), 7.15 (d, J = 8.5 Hz, 2H), 6.87 (d, J = 8.6 Hz, 2H), 3.80 (s, 3H), 3.68 (td, J = 6.9, 5.8 Hz, 2H), 2.87 (t, J = 6.9 Hz, 2H); ^{13}C NMR (151 MHz, $CDCl_3$) δ 167.43 , 158.31 , 134.62 , 131.39 , 130.85 , 129.75 , 128.55 , 126.79 , 114.12 , 55.28 , 41.19 , 34.75 .

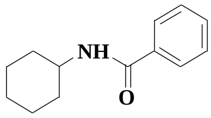

3d

N-Cyclohexyl-benzamide (3d) [25]**:** The product was obtained as white solid in 93% yield (0.57 g); MP: 146-147 ℃**;** ^{1}H NMR (600 MHz, $CDCl_3$) δ 7.87 – 7.64 (m, 2H), 7.54 – 7.34 (m, 3H), 5.99 (s, 1H), 3.98 (dt, J = 6.8,

2.8 Hz, 1H), 2.03 (dt, J = 12.5, 3.9 Hz, 2H), 1.91 – 1.55 (m, 4H), 1.53 – 1.33 (m, 2H), 1.33 – 1.09 (m, 3H); ^{13}C NMR (151 MHz, $CDCl_3$) δ 166.60 ,135.06 , 131.25 , 128.52 , 126.80 , 48.56 , 33.21 , 25.58 , 24.91

4. Synthesis and characterization of 1-Acetylimidazole [26]

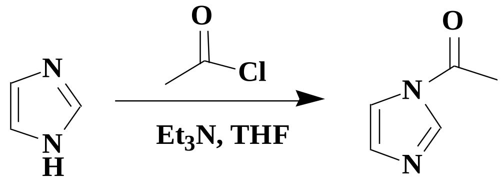

Cetyl chloride (1.26 mL, 17.4 mmol) was slowly added to a stirred solution of the imidazole （1.0g, 14.5 mmol） in anhydrous tetrahydrofuran（20 mL） under ice, under N_2. The suspension was stirred for 30 minutes and triethylamine (2.4 mL, 17.4 mmol) was added. The reaction mixture was stirred at 0 °C for 12 hours. Then the reaction mixture was filtered, and the solvent was evaporated under reduced pressure to give 1-acetylimidazole （1.53g, 13.9mmol, 94.5%） as a white solid.

5. References

1 Rao, S. N.; Mohan, D. C.; Adimurthy, S. Aibn−promoted amidation of anilines with 1, 3-diketones via oxidative cleavage of C–C bond under aerobic conditions. *Tetrahedron*, **2016**, 72, 4889-4894.

2 Marjani, A. P.; Hosseini, S. A.; Shokri, Z.; Maleki, N. Co_3O_4 nanoparticles prepared by oxidative precipitation method: an efficient and reusable heterogeneous catalyst for n -formylation of amines. *Research on Chemical Intermediates*, **2017**, 43, 413-422.

3 Schwenger, A.; Frey, W.; Richert, C. reagents with a crystalline coat. *Angew. Chem. Int. Ed.*, **2016**, 55, 13617-13617.

4 Al-Sehemi, A. G., Atkinson, R. S., & Fawcett, J. kinetic resolution of amines with enantiopure 3-*N,N*-diacylaminoquinazolin-4(3h)-ones. *Journal of the Chemical Society Perkin Transactions*, **2002**, 33, 257-274.

5 Yoshida, T.; Kawamura, S.; Nakata, K. Chemoselective n-acetylation of primary aliphatic amines promoted by pivalic or acetic acid using ethyl acetate as an acetyl donor. *Tetrahedron Letters*. **2017**, 58, 1181.
6 Sheng, H.; Zeng, R.; Wang, W.; Luo, S.; Feng, Y.; Liu, J. An efficient heterobimetallic lanthanide alkoxide catalyst for transamidation of amides under solvent‐free conditions. *Adv. Synth. Catal*. **2017**, 359, 302.
7 Kalla, R. M. N.; Lim, J.; Bae, J.; Kim, I. Sulfated choline ionic liquid-catalyzed acetamide synthesis by grindstone method. *Tetrahedron Letters*. **2017**, 58, 1595-1599.
8 Debnath, P.; Baeten, M.; Lefevre, N.; Van Daele, S.; Maes, B. U. W. synthesis of secondary amides from N‐substituted amidines by tandem oxidative rearrangement and isocyanate elimination. Cheminform. *Advanced Synthesis and Catalysis*. **2015**, 46, 197–209.
9 Broese, T.; Francke, R. Electrosynthesis using a recyclable mediator–electrolyte system based on ionically tagged phenyl iodide and 1,1,1,3,3,3-hexafluoroisopropanol. *Organic Letters*. **2016**. 18, 5896.
10 Borror, A. L.; Chinoporos, E.; Filosa, M. P.; Herchen, S. R.; Petersen, C. P.; Stern, C. A. regioselectivity of electrophilic aromatic substitution: syntheses of 6- and 7-sulfamoylindolines and -indoles. *Journal of Organic Chemistry*. **1988**, 53, 2047
12 Patil, V. V.; Shankarling, G. S. Steric-hindrance-induced regio- and chemoselective oxidation of aromatic amines. *Journal of Organic Chemistry*. **2016**, 47, 7876.
13 Pan, X.; Dong, J.; Shi, Y.; Shao, R.; Wei, F.; Wang, J. Discovery of novel bcr-abl inhibitors with diacylated piperazine as the flexible linker. *Organic & Biomolecular Chemistry*. **2015**, 13, 7050.
14 Chang, D.; Zhu, D.; Zou, P., Shi, L. Cheminform abstract: cleavage of C-N bonds in guanidine derivatives and its relevance to efficient C-N bonds formation. *Tetrahedron,* **2015**, 71, 1684-1693.
15 Nitta, A.; Fujii, H.; Sakami, S.; Nishimura, Y.; Ohyama, T.; Satoh, M. (3R)-3-amino-4-(2,4,5-trifluorophenyl)-N-{4-[6-(2-methoxyethoxy)benzothiazol-2-yl]tetrahydropyran-4-ylbutanamide as a potent dipeptidyl peptidase IV inhibitor for the treatment of type 2 diabetes. *Bioorganic & Medicinal Chemistry Letters*. **2008**, 18, 5435-5438.

16 Rothweiler, U.; Stensen, W.; Brandsdal, B. O.; Isaksson, J.; Leeson, F. A.; Engh, R. A. Probing the atp-binding pocket of protein kinase DYRK1A with benzothiazole fragment molecules. *Journal of Medicinal Chemistry*. **2016**, 59, 9814.
17 Copinga, S.; Tepper, P. G.; Grol, C. J.; Horn, A. S.; Dubocovich, M. L. 2-amido-8-methoxytetralins: a series of nonindolic melatonin-like agents. *Journal of Medicinal Chemistry*. **1993**, 36, 2891-2898.
18 Tichý, M.; Holanová, J.; Starý, I.; Stará, I. G.; Závada, J. Axially chiral selectors of C2 symmetry bound to silica: synthesis and hplc-evaluation. *Collection of Czechoslovak Chemical Communications*. **1995**, 60, 645-658.
19 Patre, R. E.; Mal, S.; Nilkanth, P. R.; Ghorai, S. K.; Deshpande, S. H.; El, Q. M. First report on bio-catalytic N-formylation of amines using ethyl formate. *Chem. Commun.* **2017**, 53, 2382.
20 Liu, X. F.; Ma, R.; Qiao, C.; Cao, H.; He, L. N. Fluoride-catalyzed methylation of amines by reductive functionalization of co2 with hydrosilanes. *Eur. J. Chem.* **2016**, 22, 16489.
21 Ke, Z.; Zhang, Y.; Cui, X.; Shi, F. supported nano-gold-catalyzed N-formylation of amines with paraformaldehyde in water under ambient conditions. *Green Chemistry*. **2016**, 47, 808-816.
22 Zhang, J.; Ma, Y.; Ma, Y. Synthesis of secondary amides through palladium(Ⅱ)-catalyzed aminocarbonylation of arylboronic acids with amines / hydrazines and CO. *European Journal of Organic Chemistry*, **2018**, 2018, 1720.
23 Viaud, P.; Coeffard, V.; Thobiegautier, C.; Beaudet, I.; Galland, N.; Quintard, J. P. Electrochemical cleavage of sulfonamides: an efficient and tunable strategy to prevent β-fragmentation and epimerization. *Organic Letters*. **2012**,14, 942-945.
24 Minor, D. L.; Wyrick, S. D.; Charifson, P. S.; Watts, V. J.; Nichols, D. E.; Mailman, R. B. Synthesis and molecular modeling of 1-phenyl-1,2,3,4-tetrahydroisoquinolines and related 5,6,8,9-tetrahydro-13bh-dibenzo[a,h]quinolizines as D1 dopamine antagonists. *Journal of Medicinal Chemistry*. **1994**, 37, 4317.
25 Das, B. G.; Ghorai, P. Stereoselective direct reductive amination of ketones with electron-deficient amines using ReO /NaPF catalyst. *Organic & Biomolecular Chemistry*. **2013**, 11, 4379-4382.

26 Harper, J. L.; Smith, R. A. J.; Bedford, J. J.; Leader, J. P. Synthesis, acidity and ^{19}F nmr characteristics of imidazoles bearing 1-fluorinated substituents with potential application as probes for intracellular ph determination. *Tetrahedron*. **1997**, 53, 8211-8224.

6. [1] H NMR and [13] C NMR spectra of N-Acetamides, N-Formamides and N- Benzoylation

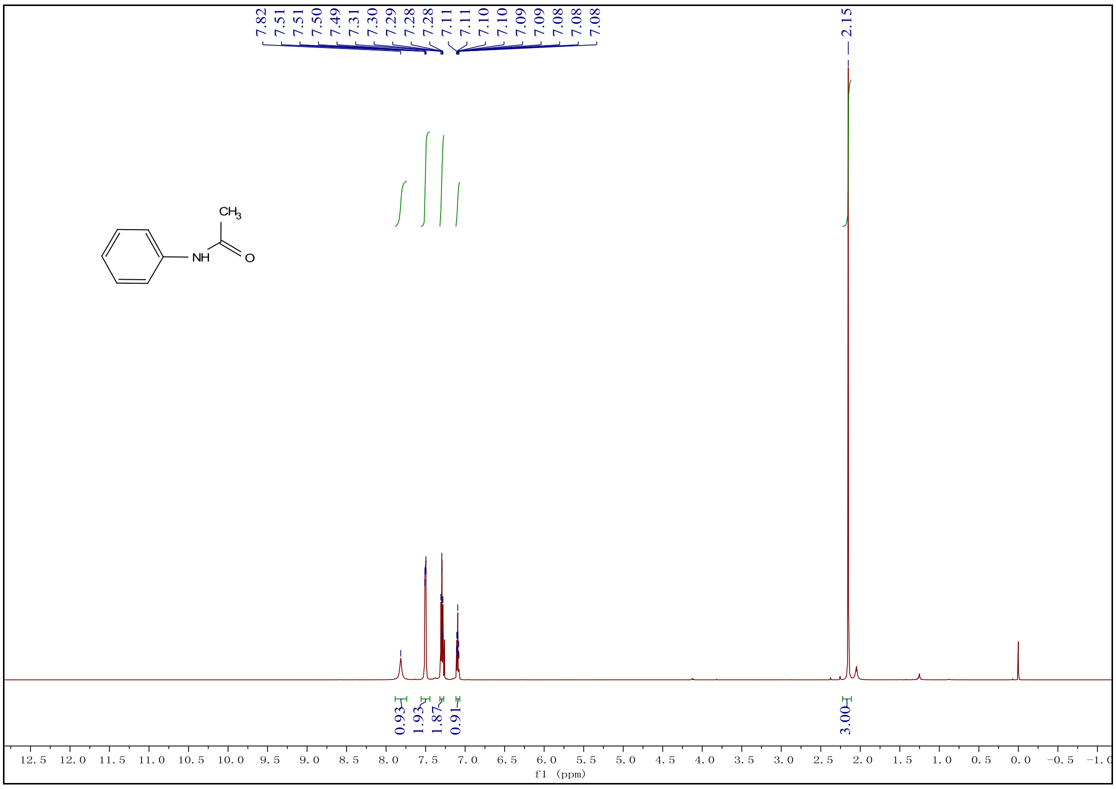

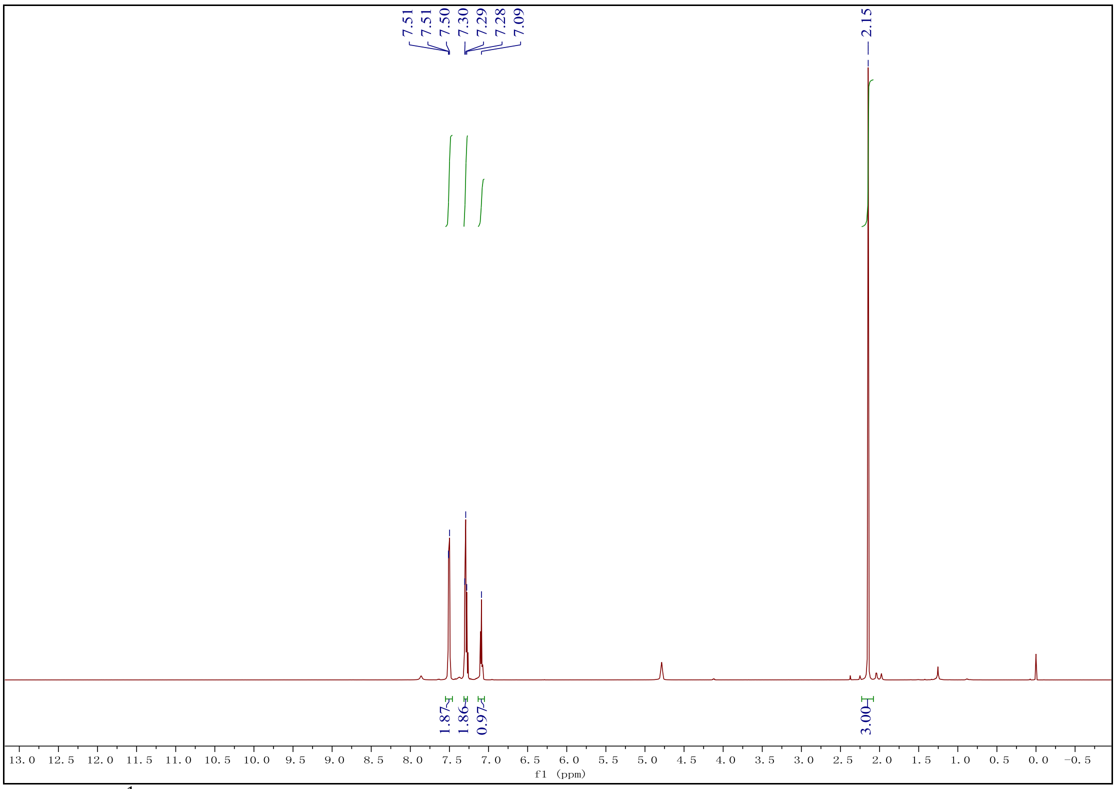

Figure 1. ^{1}H NMR spectrum of **1a** (Solvent: $CDCl_3$)

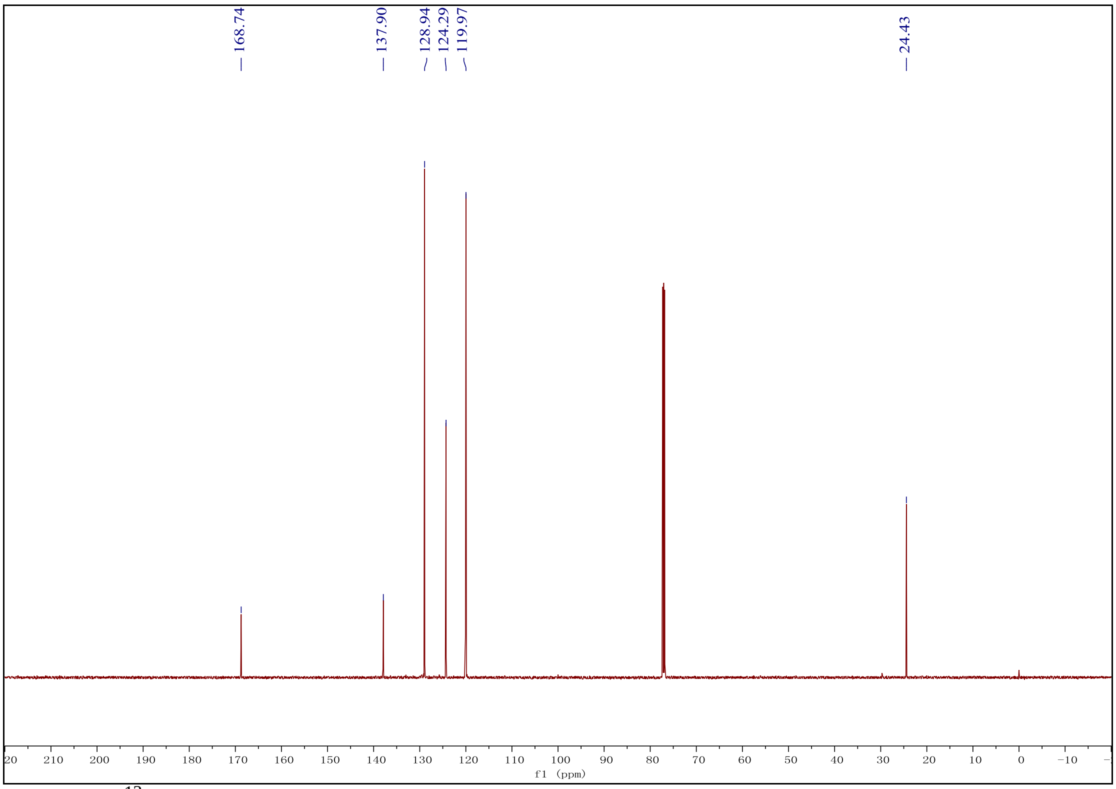

Figure 2. ^{13}C NMR spectrum of **1a** (Solvent: $CDCl_3$)

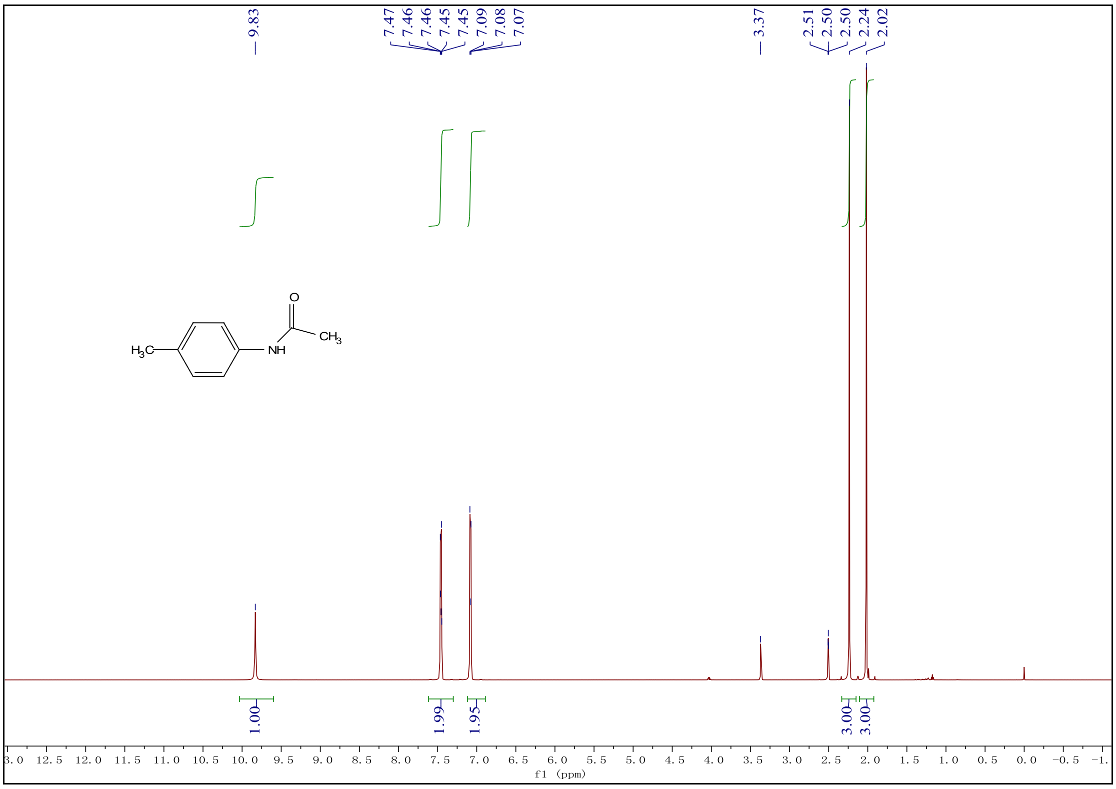

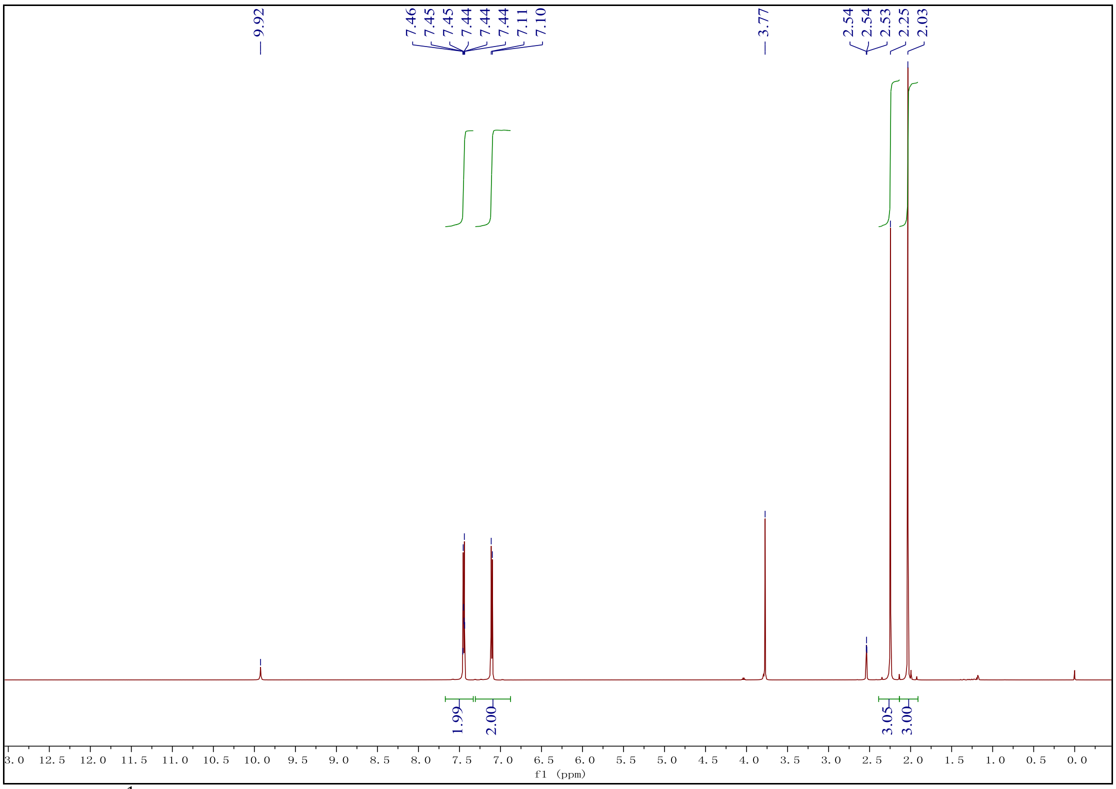

Figure 3. ^{1}H NMR spectrum of **1b** (Solvent: DMSO-d6)

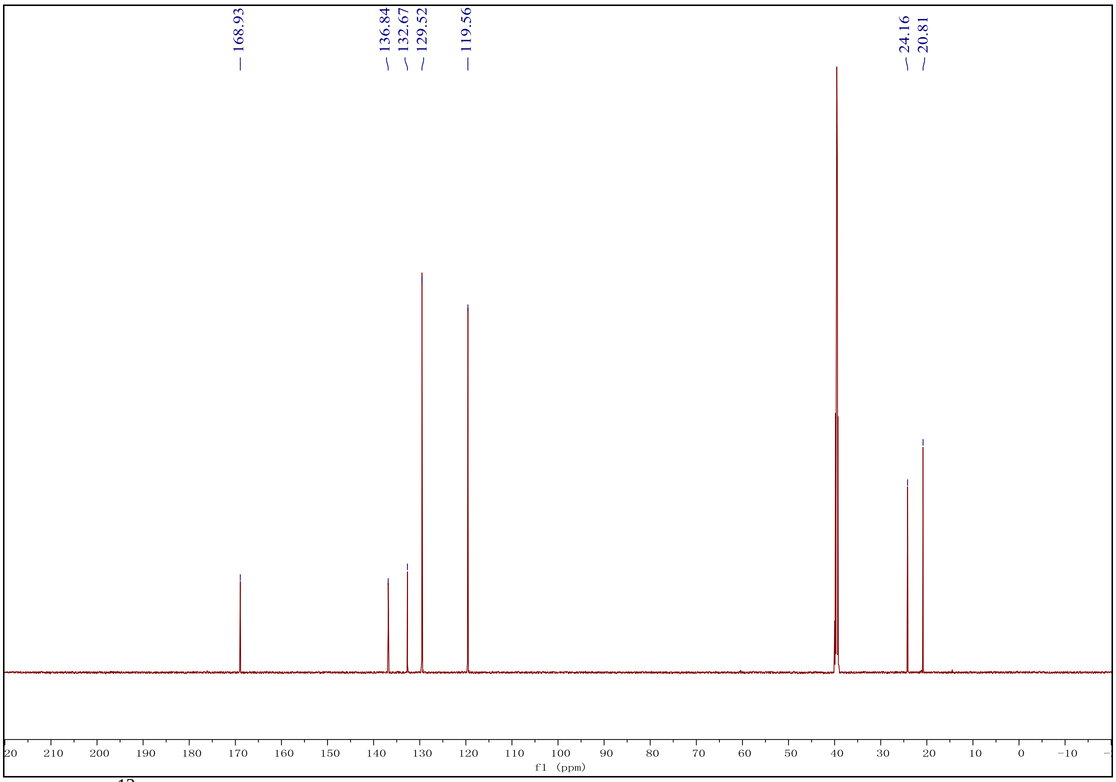

Figure 4. ^{13}C NMR spectrum of **1b** (Solvent: DMSO-d6)

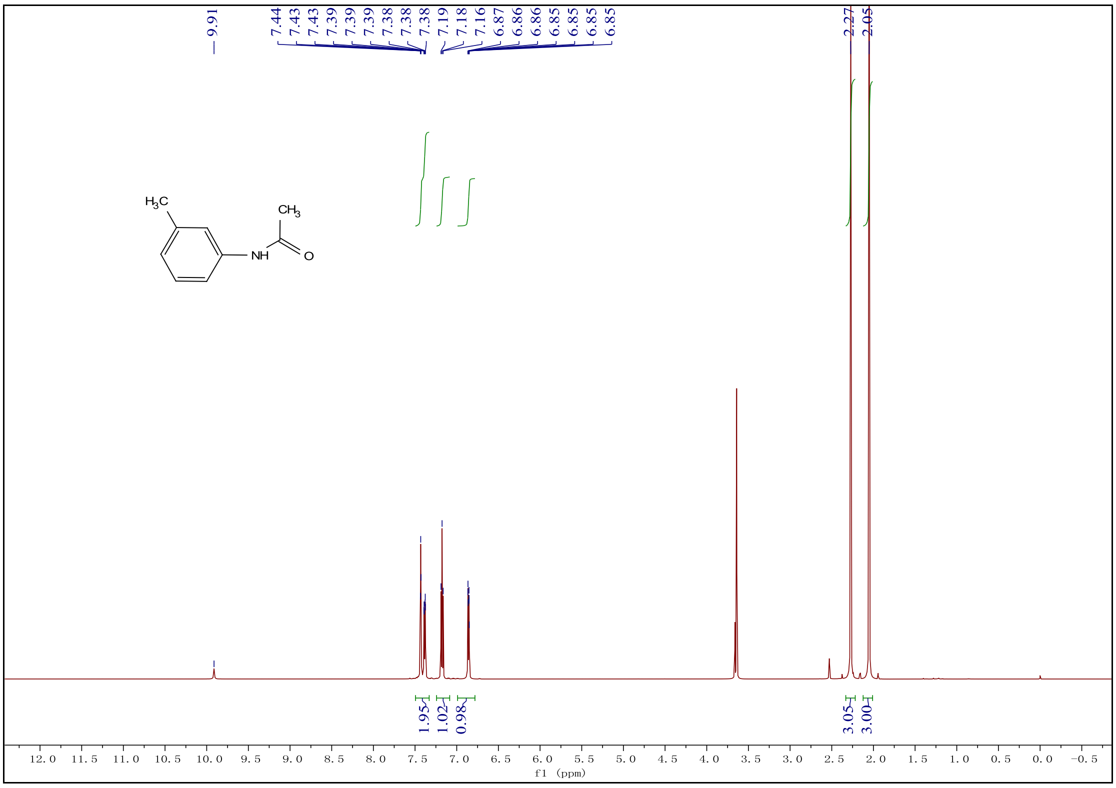

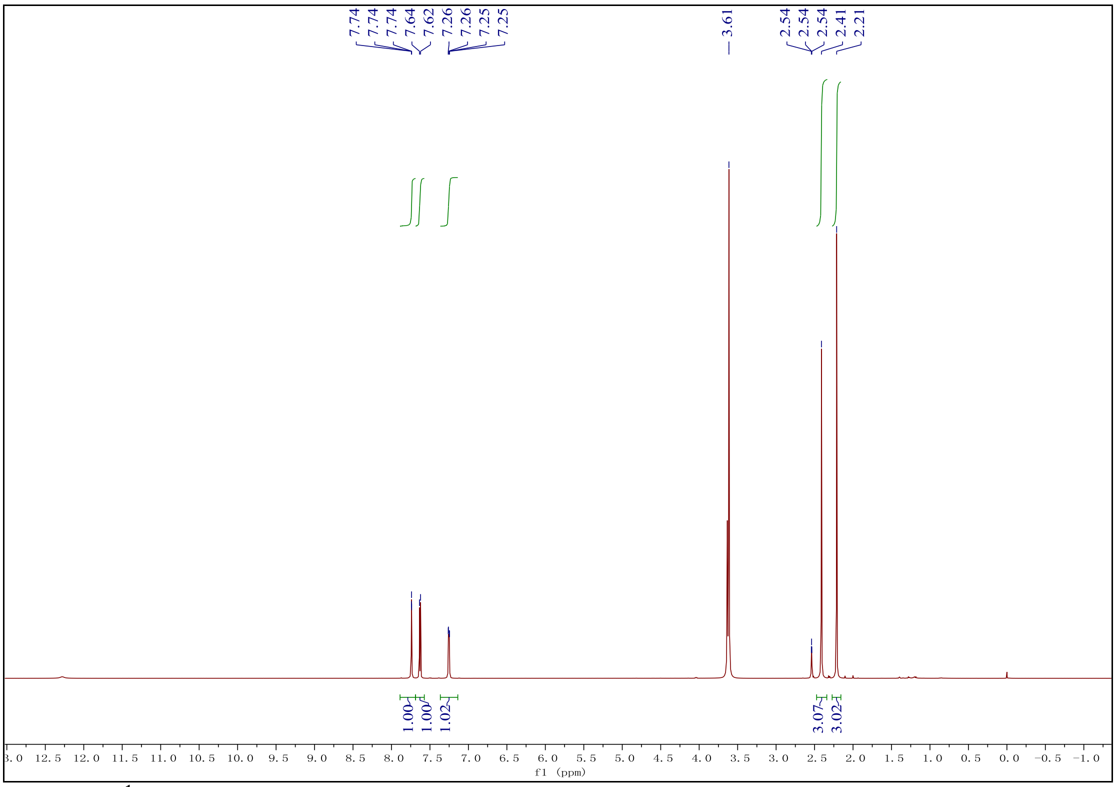

Figure 5. ^{1}H NMR spectrum of **1c** (Solvent: DMSO-d6)

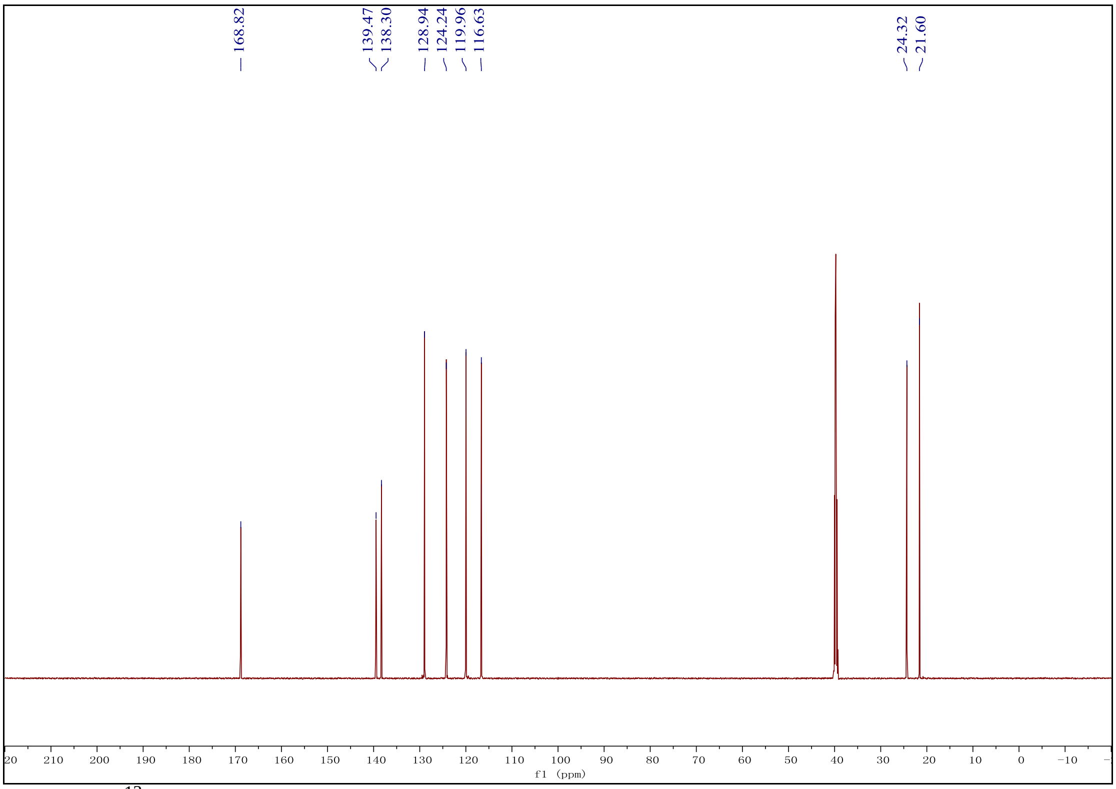

Figure 6. ^{13}C NMR spectrum of **1c** (Solvent: DMSO-d6)

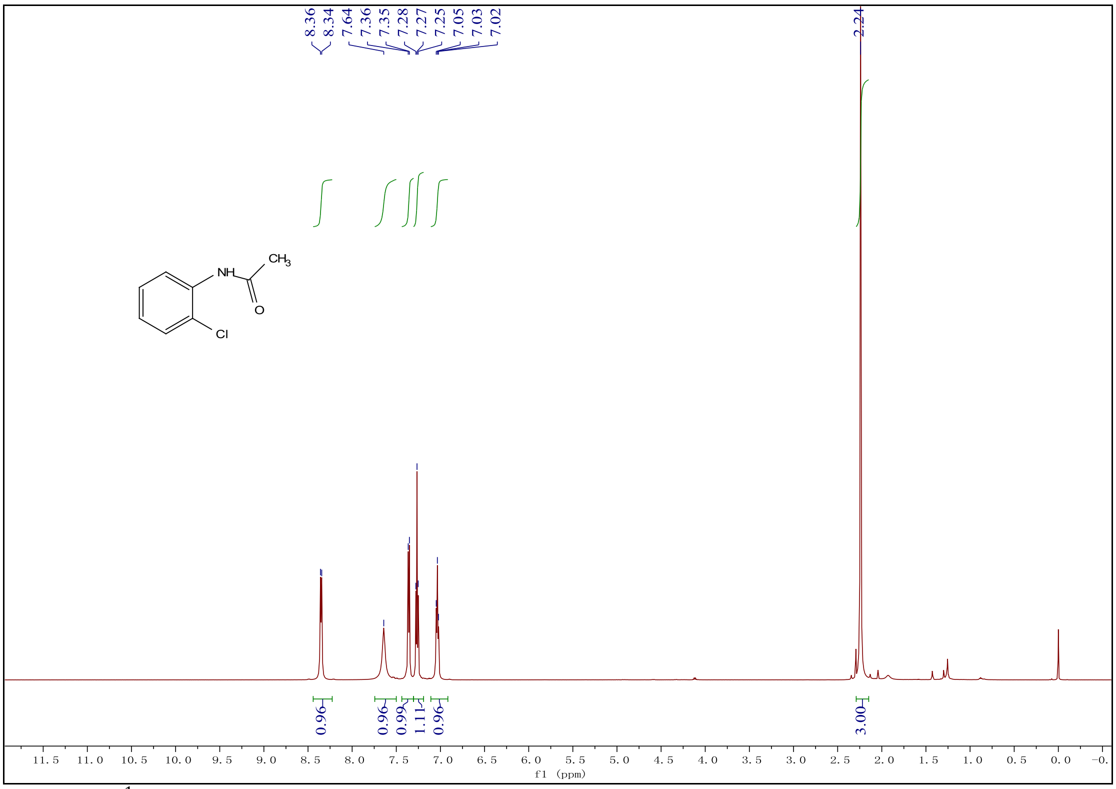

Figure 7. ^{1}H NMR spectrum of **1d** (Solvent: $CDCl_3$)

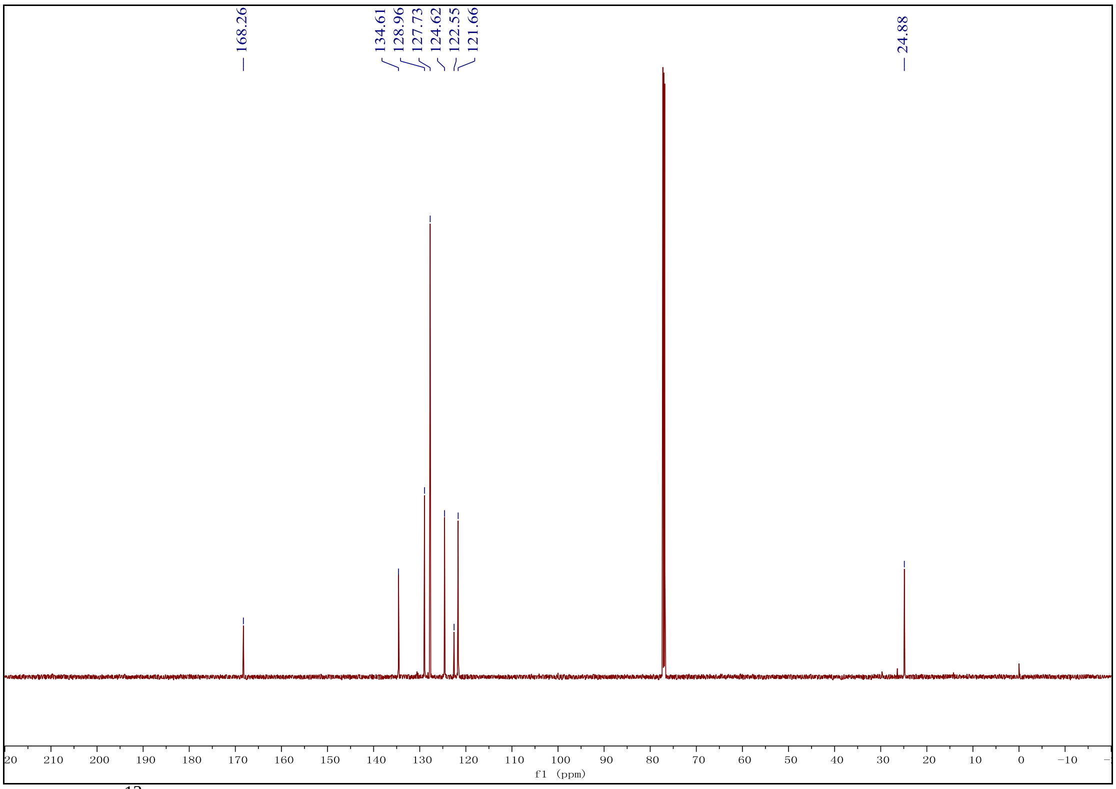

Figure 8. ^{13}C NMR spectrum of **1d** (Solvent: $CDCl_3$)

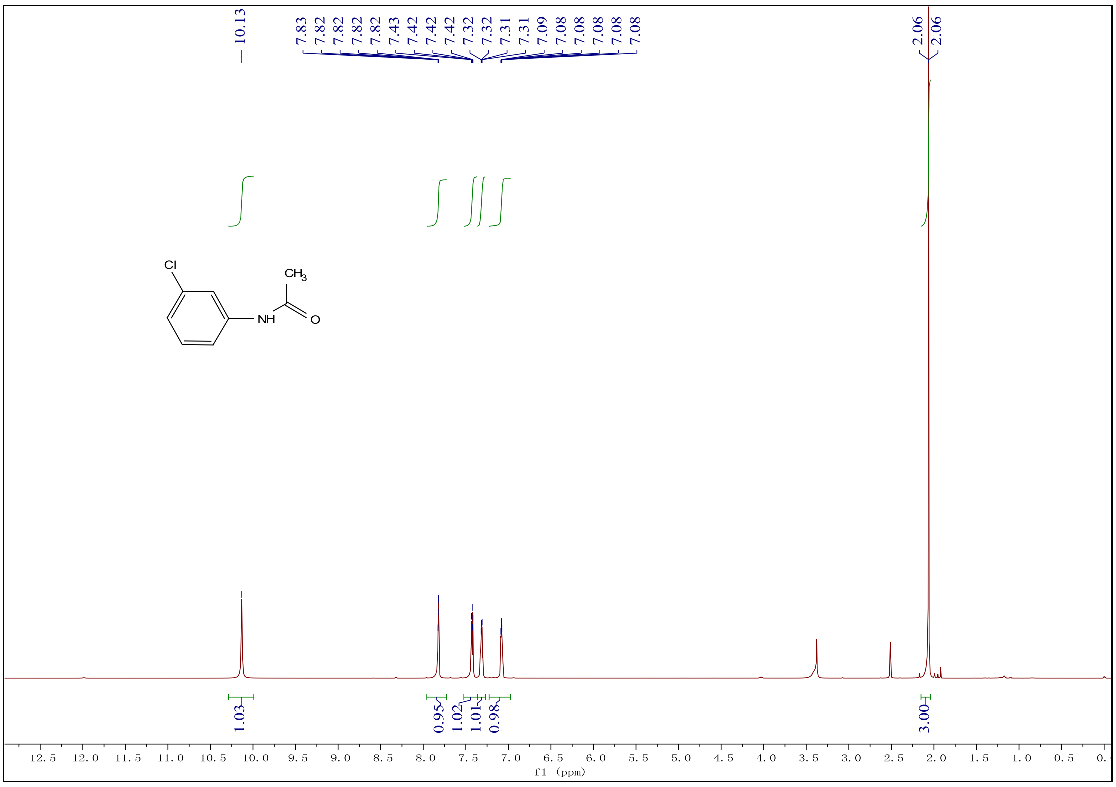

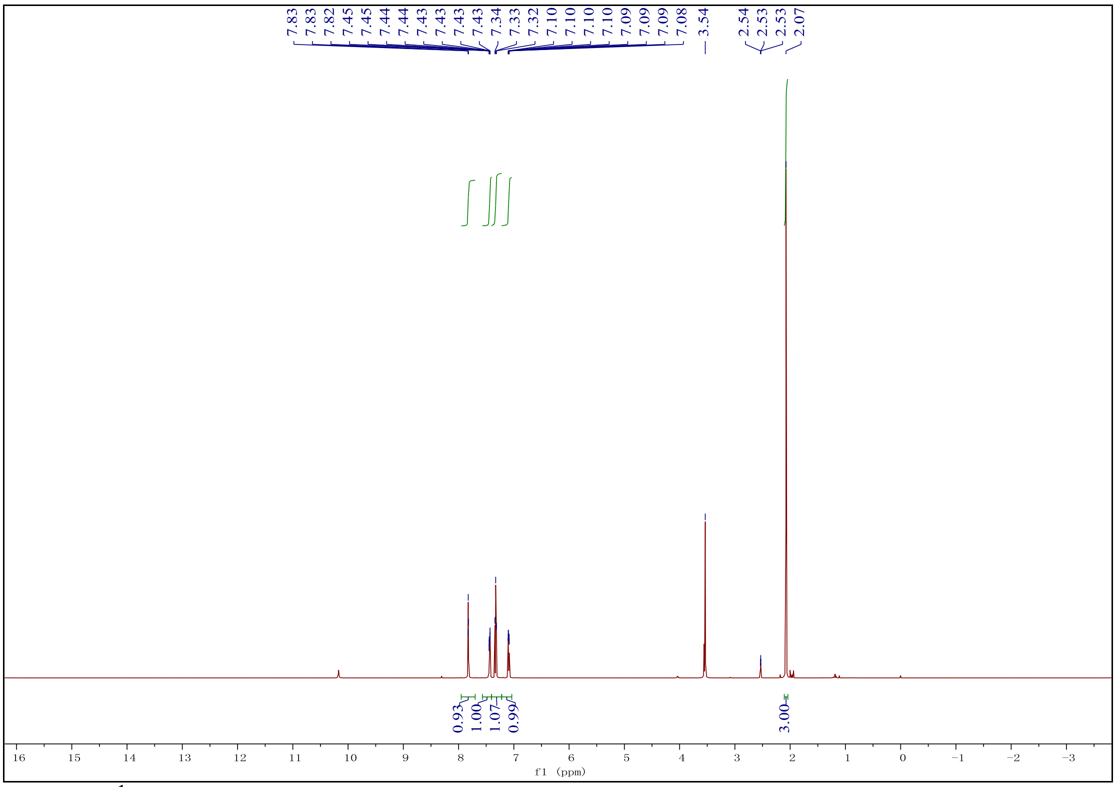

Figure 9. ^{1}H NMR spectrum of **1e**(Solvent: DMSO-d6)

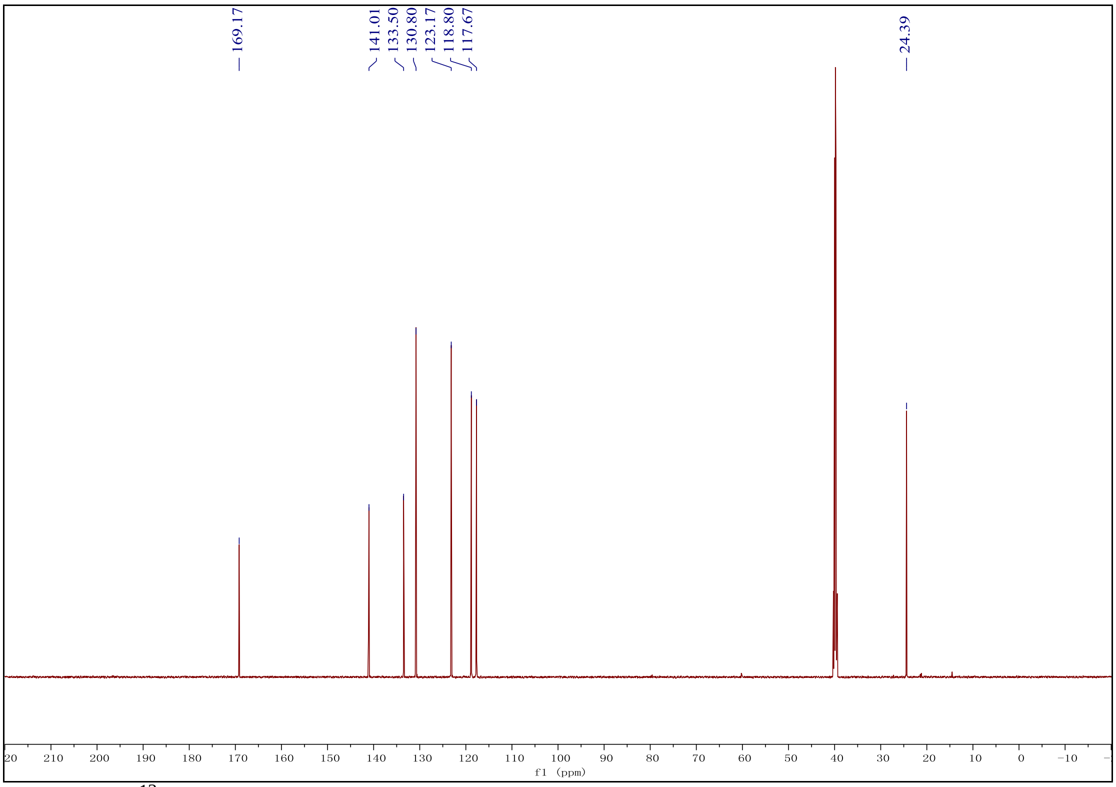

Figure 10. ${}^{13}C$ NMR spectrum of **1e** (Solvent: DMSO-d6)

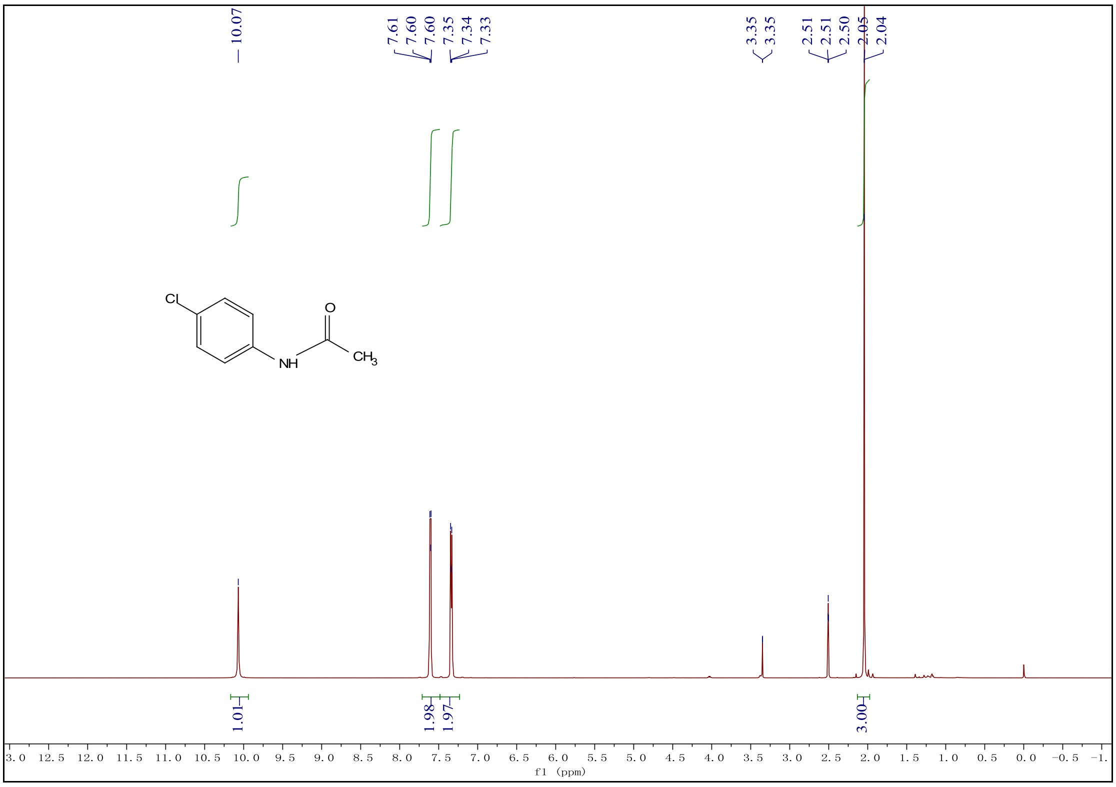

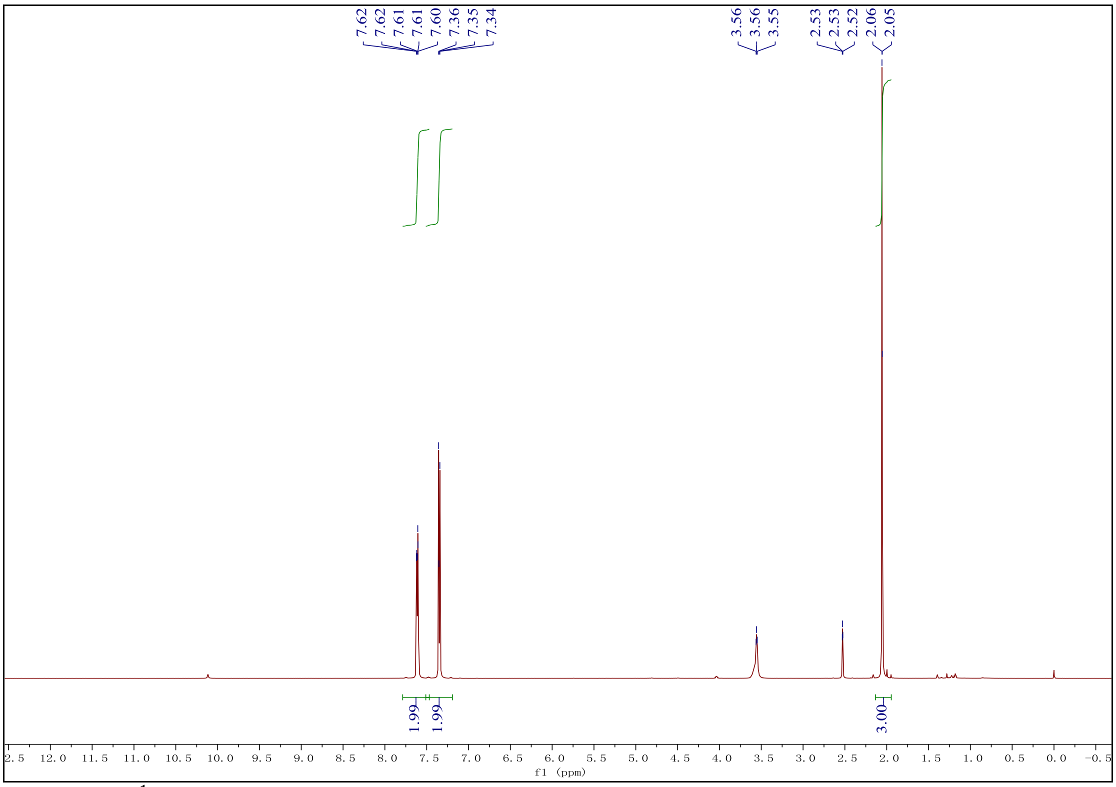

Figure 11. ^{1}H NMR spectrum of **1f** (Solvent: DMSO-d6)

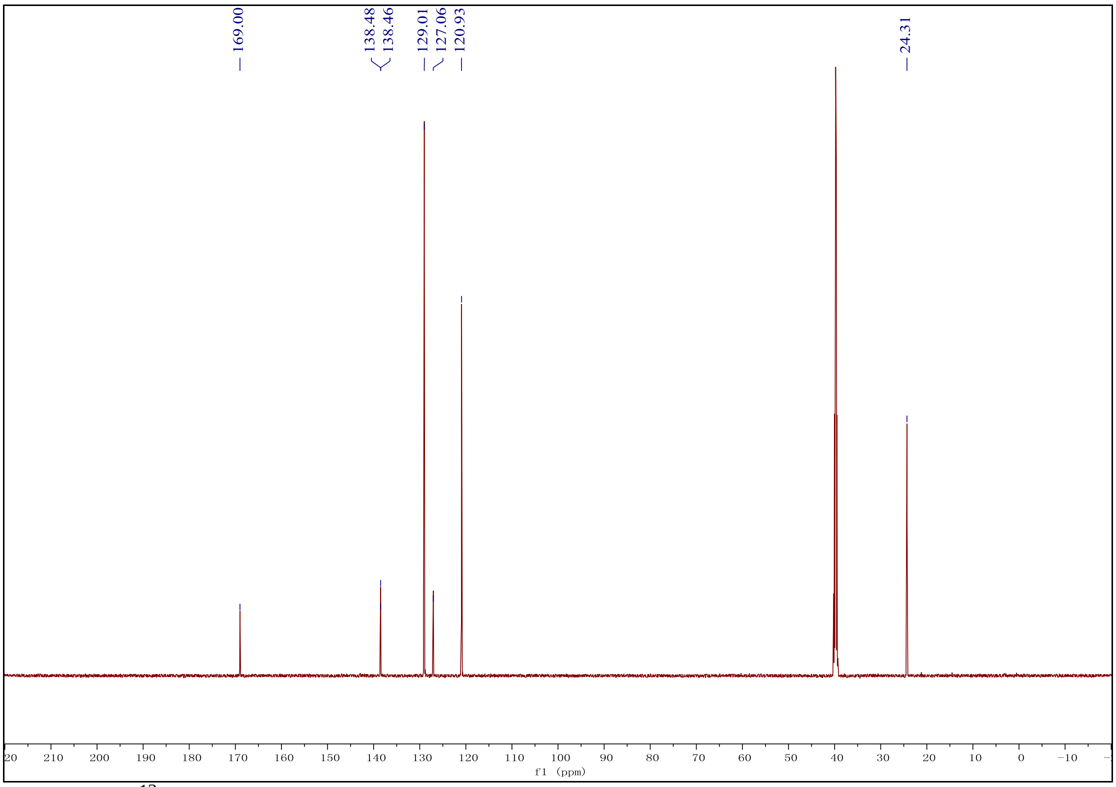

Figure 12. ^{13}C NMR spectrum of **1f** (Solvent: DMSO-d6)

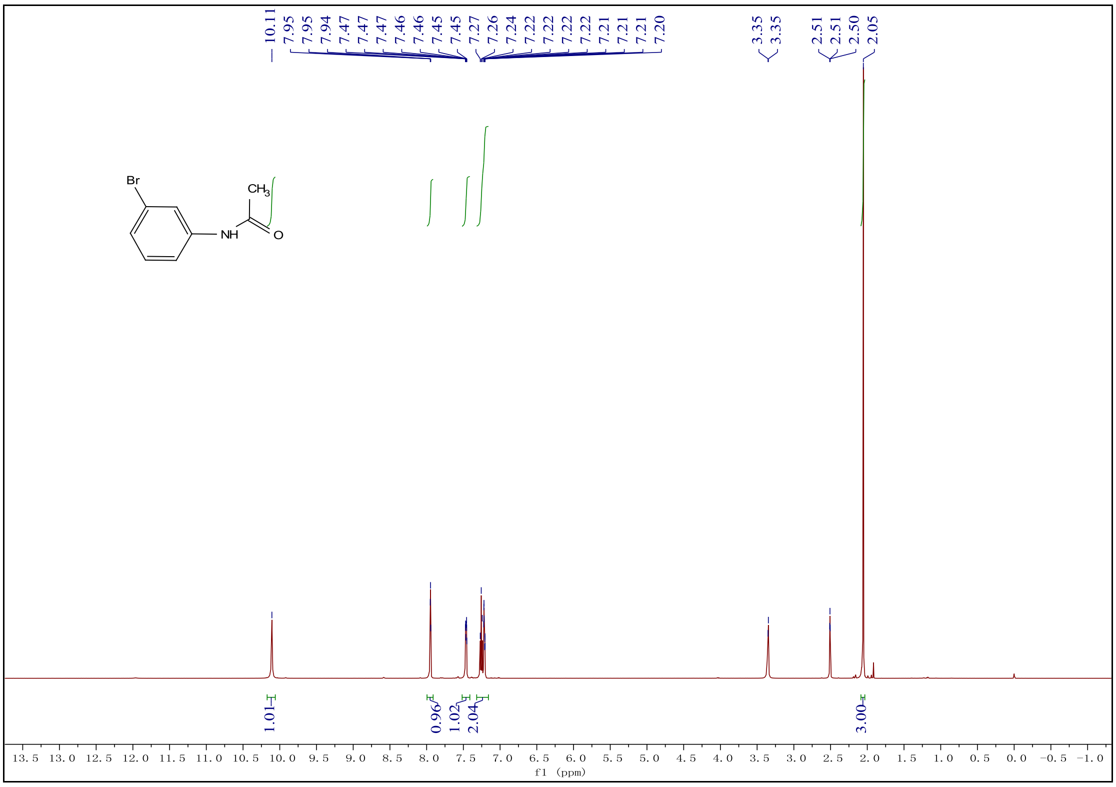

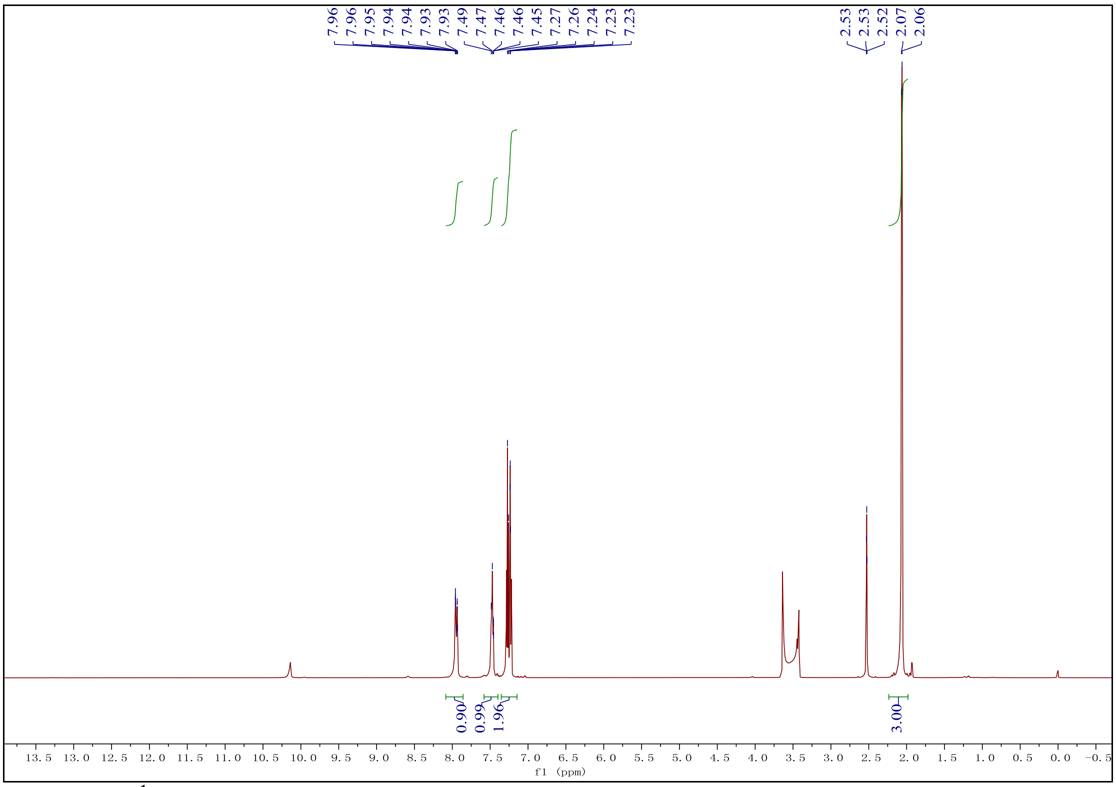

Figure 13. ^{1}H NMR spectrum of **1g** (Solvent: DMSO-d6)

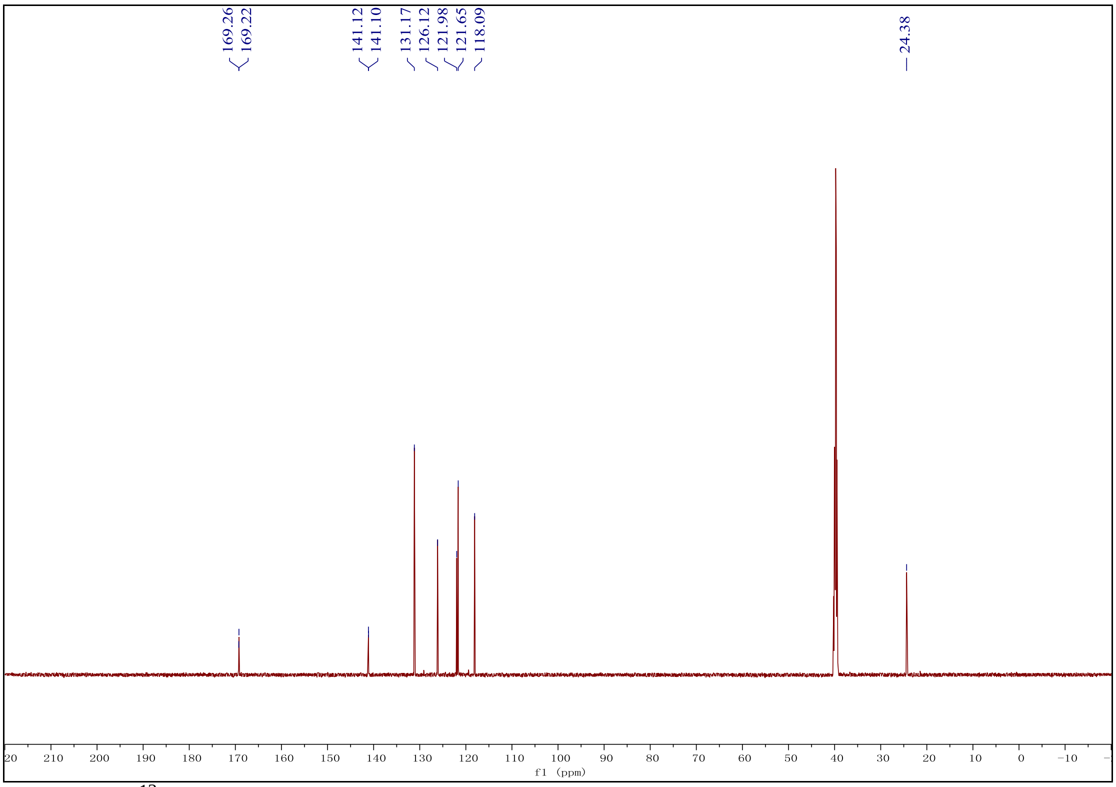

Figure 14. ^{13}C NMR spectrum of **1g** (Solvent: DMSO-d6)

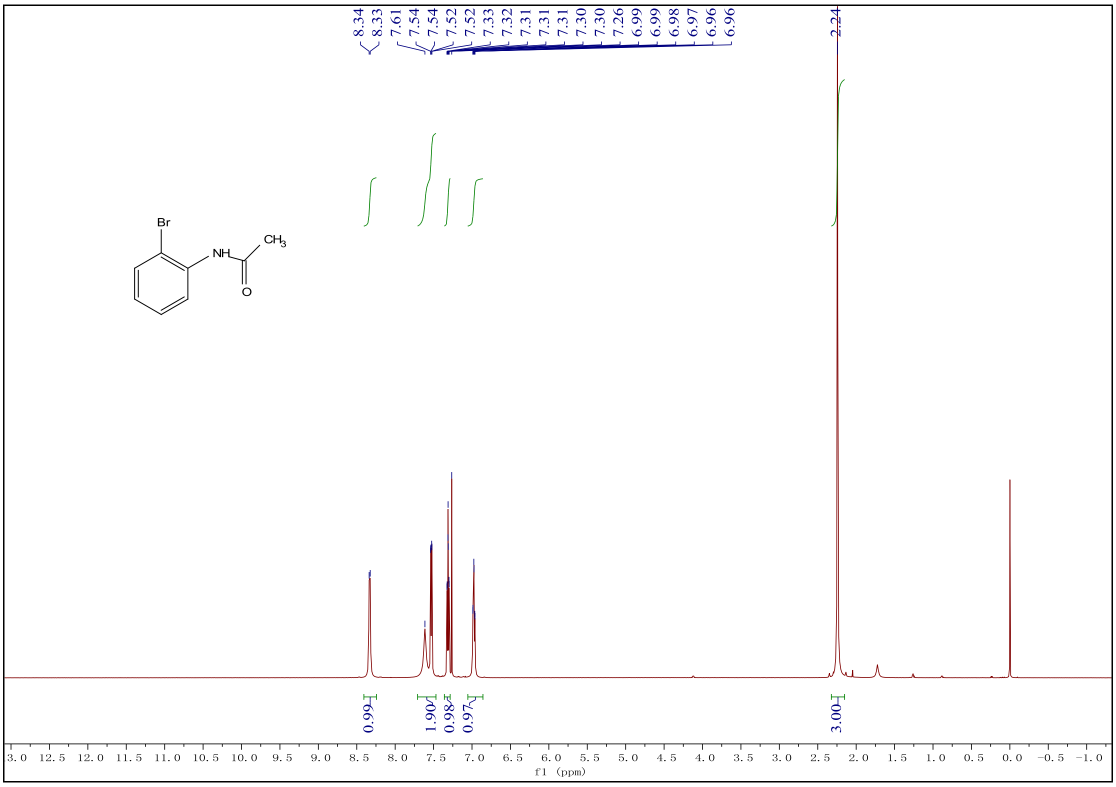

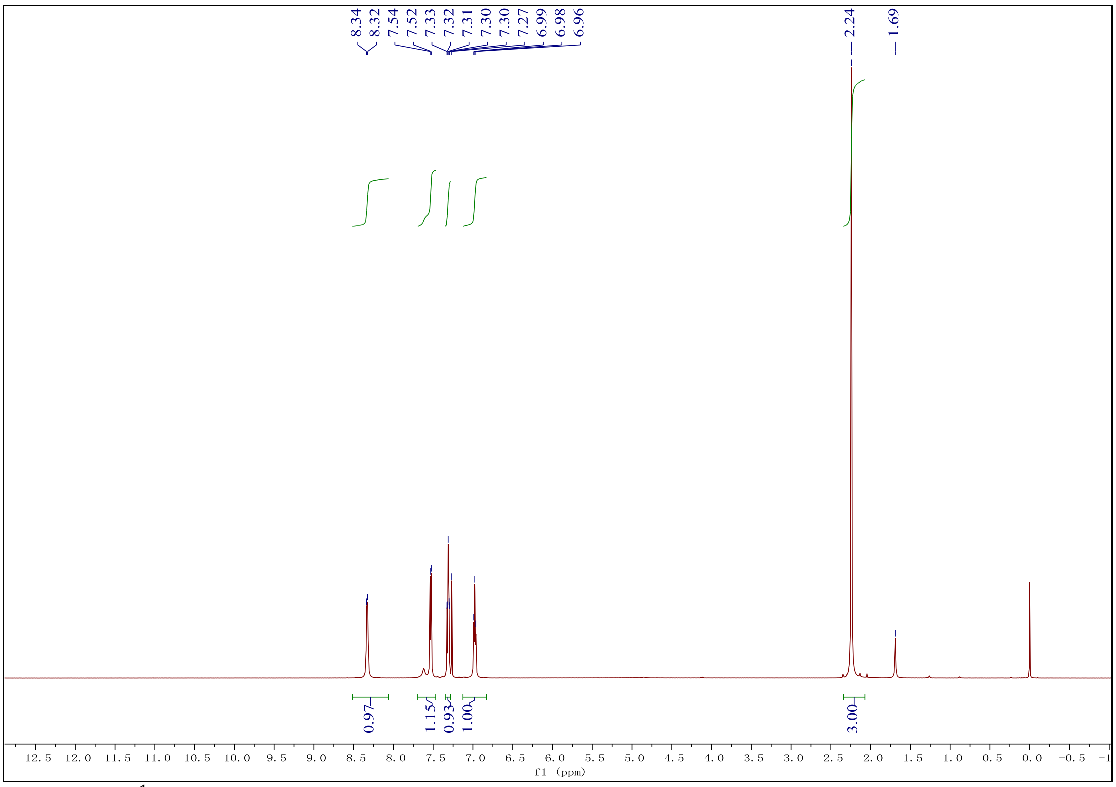

Figure 15. ^{1}H NMR spectrum of **1h** (Solvent: $CDCl_3$)

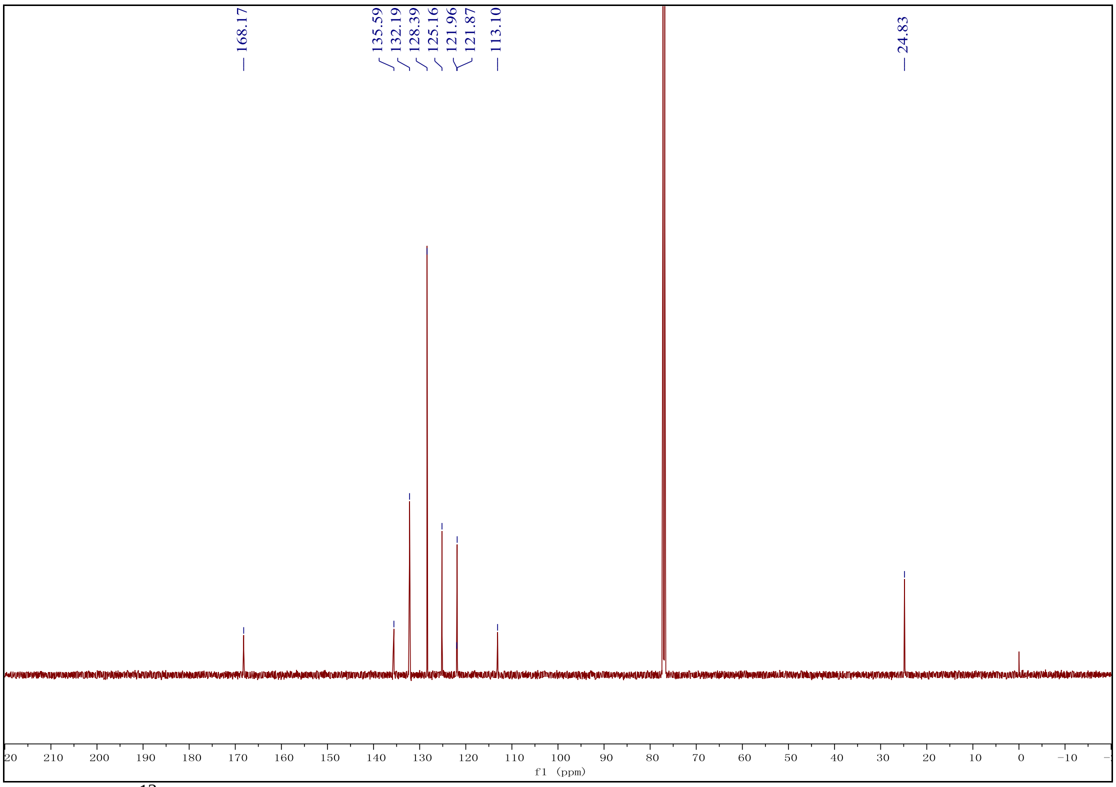

Figure 16. ^{13}C NMR spectrum of **1h** (Solvent: $CDCl_3$)

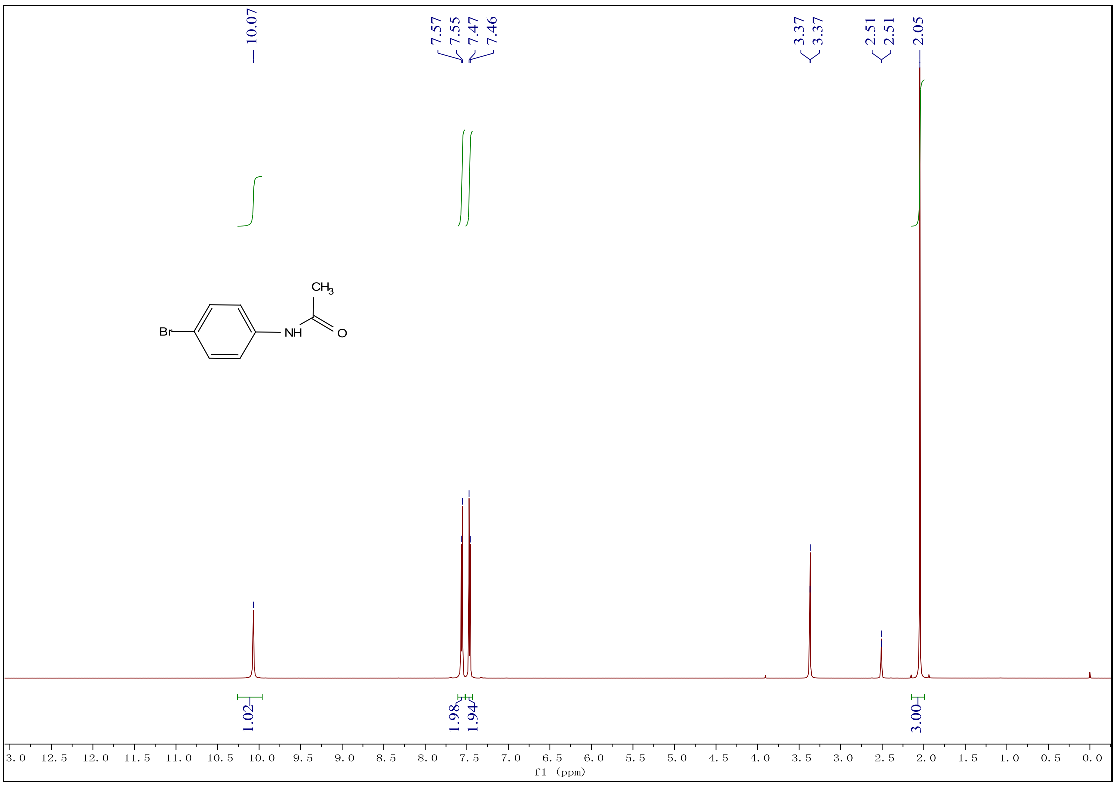

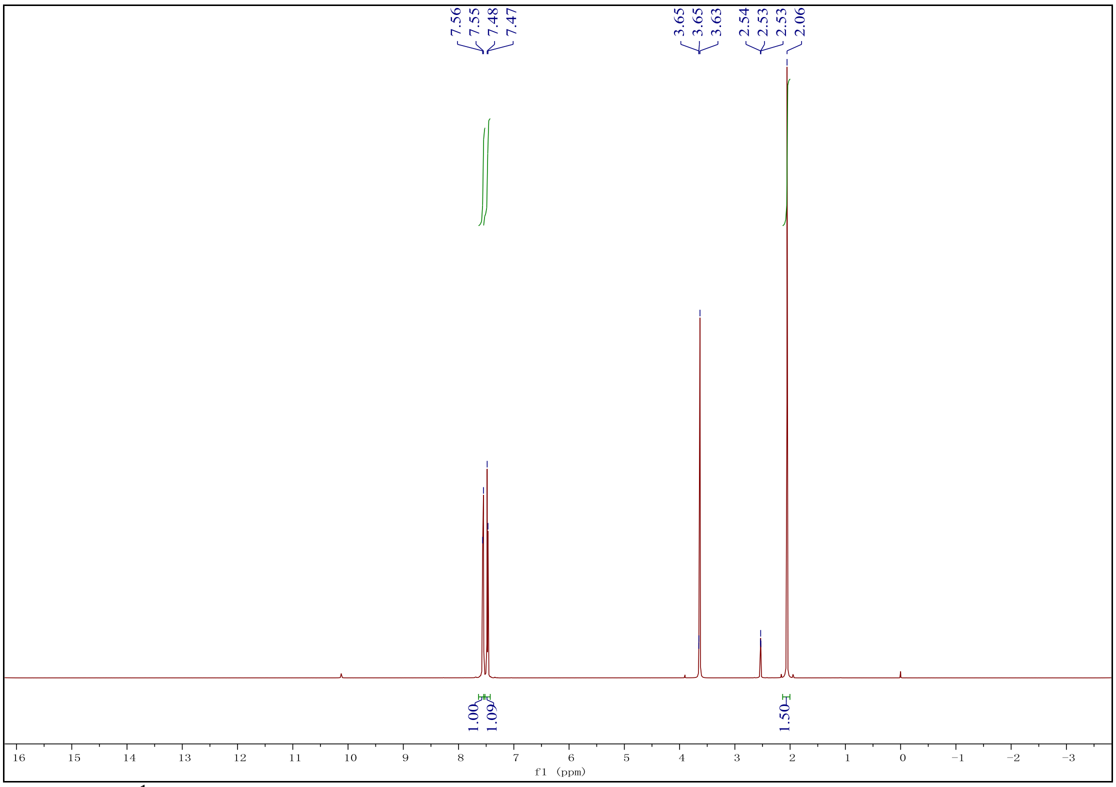

Figure 17. ^{1}H NMR spectrum of **1i** (Solvent: DMSO-d6)

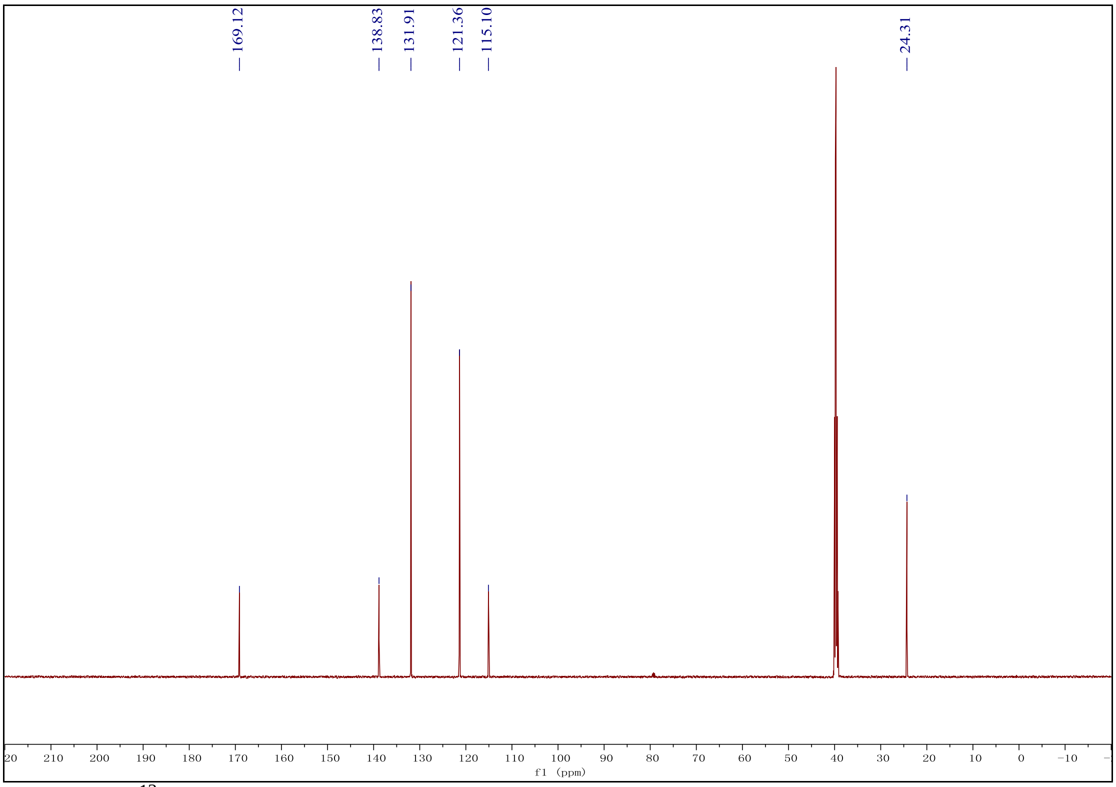

Figure 18. ^{13}C NMR spectrum of **1i** (Solvent: DMSO-d6)

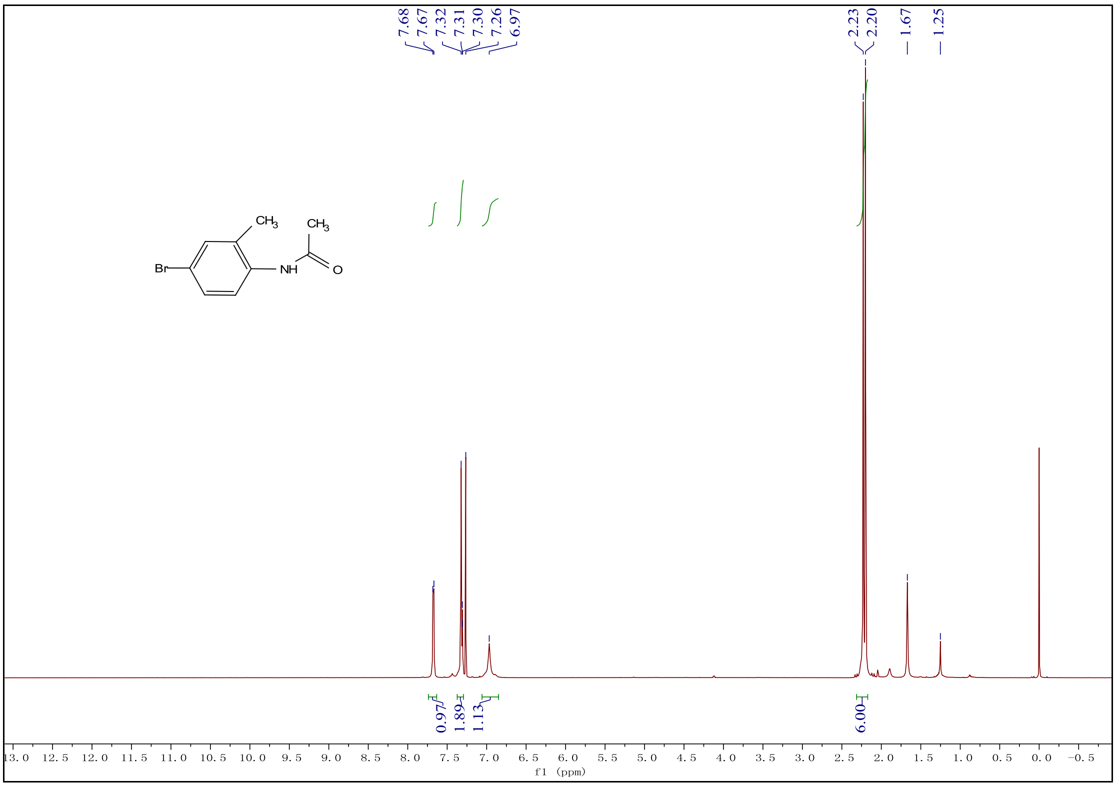

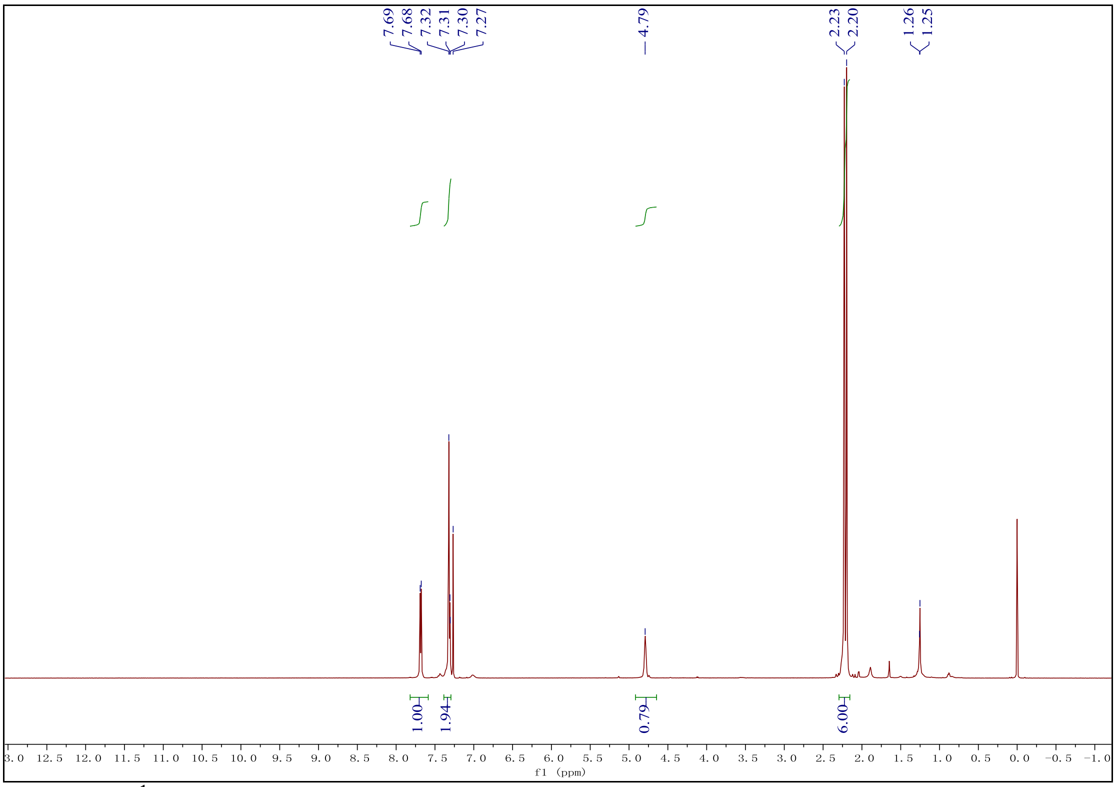

Figure 19. ^{1}H NMR spectrum of **1j** (Solvent: $CDCl_3$)

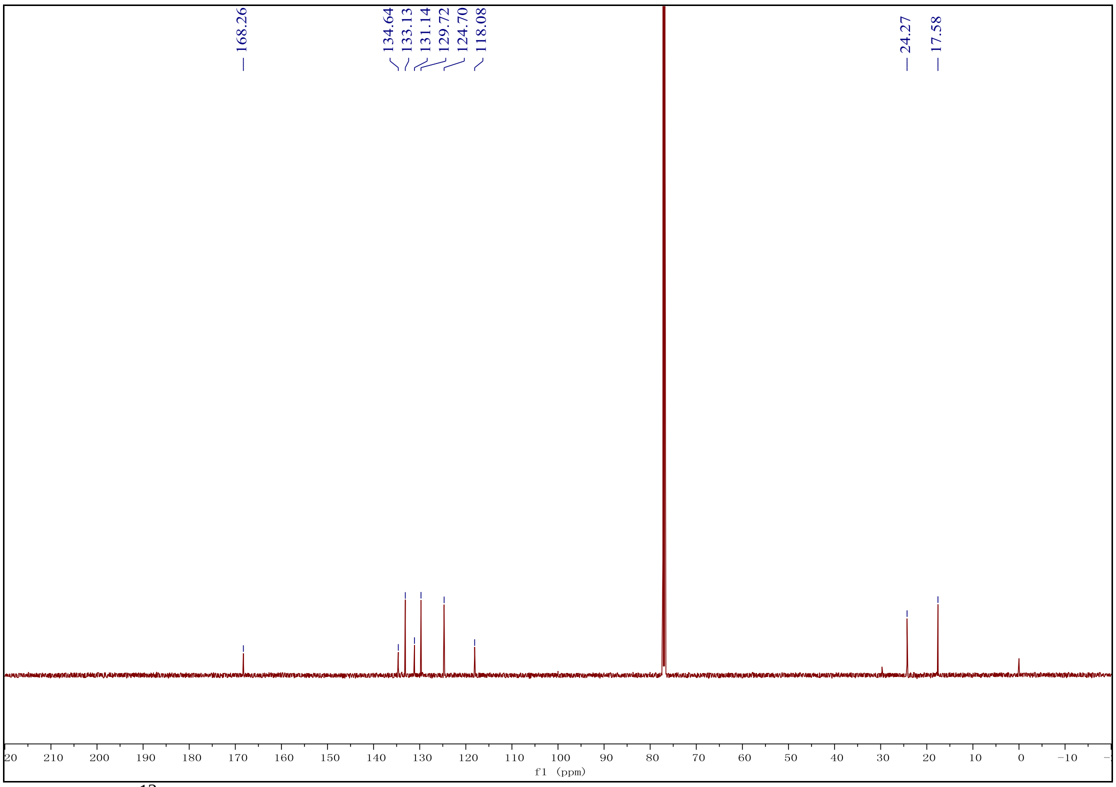

Figure 20. ^{13}C NMR spectrum of **1j** (Solvent: $CDCl_3$)

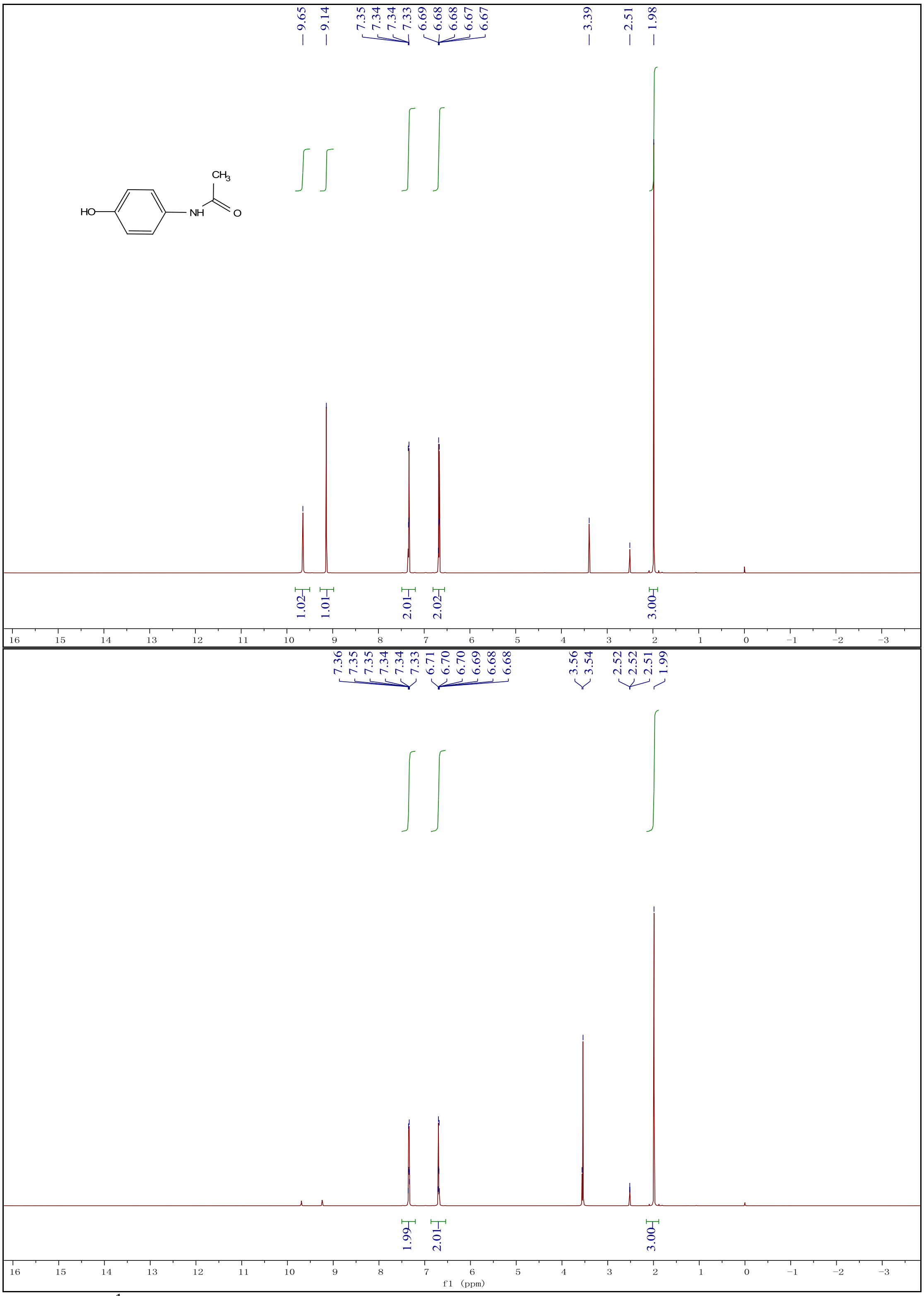

Figure 21. ^{1}H NMR spectrum of **1k** (Solvent: DMSO-d6)

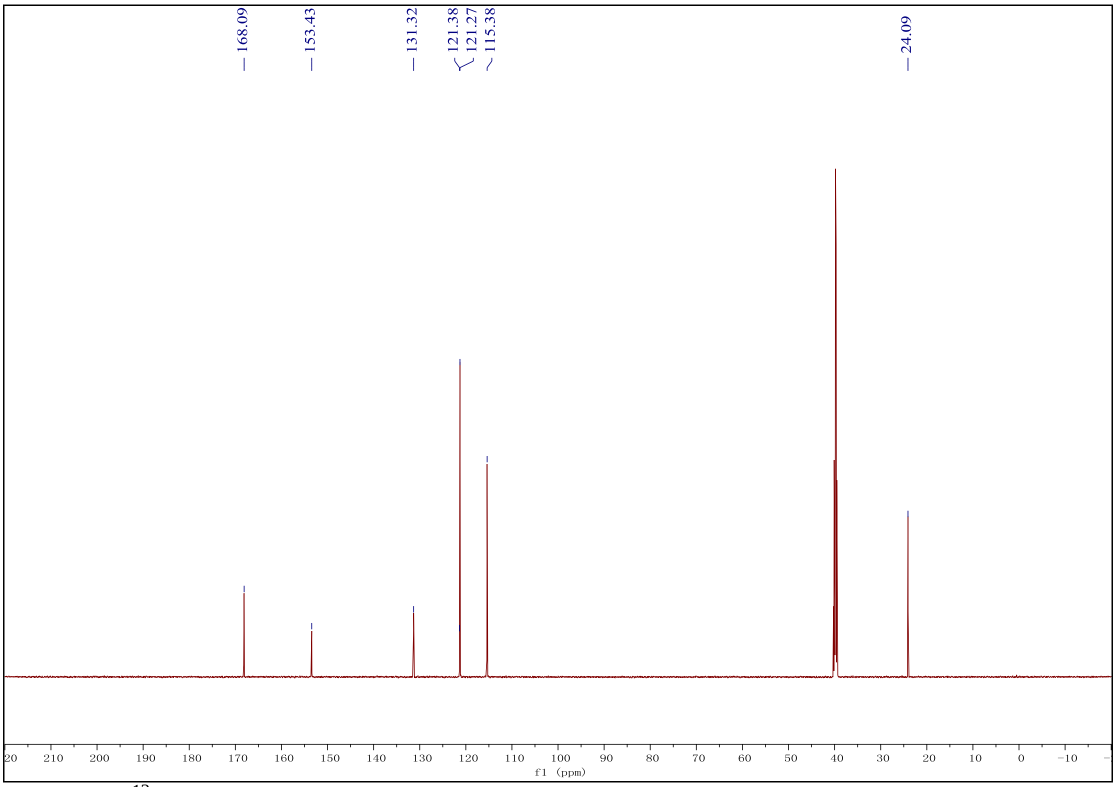

Figure 22. ^{13}C NMR spectrum of **1k** (Solvent: DMSO-d6)

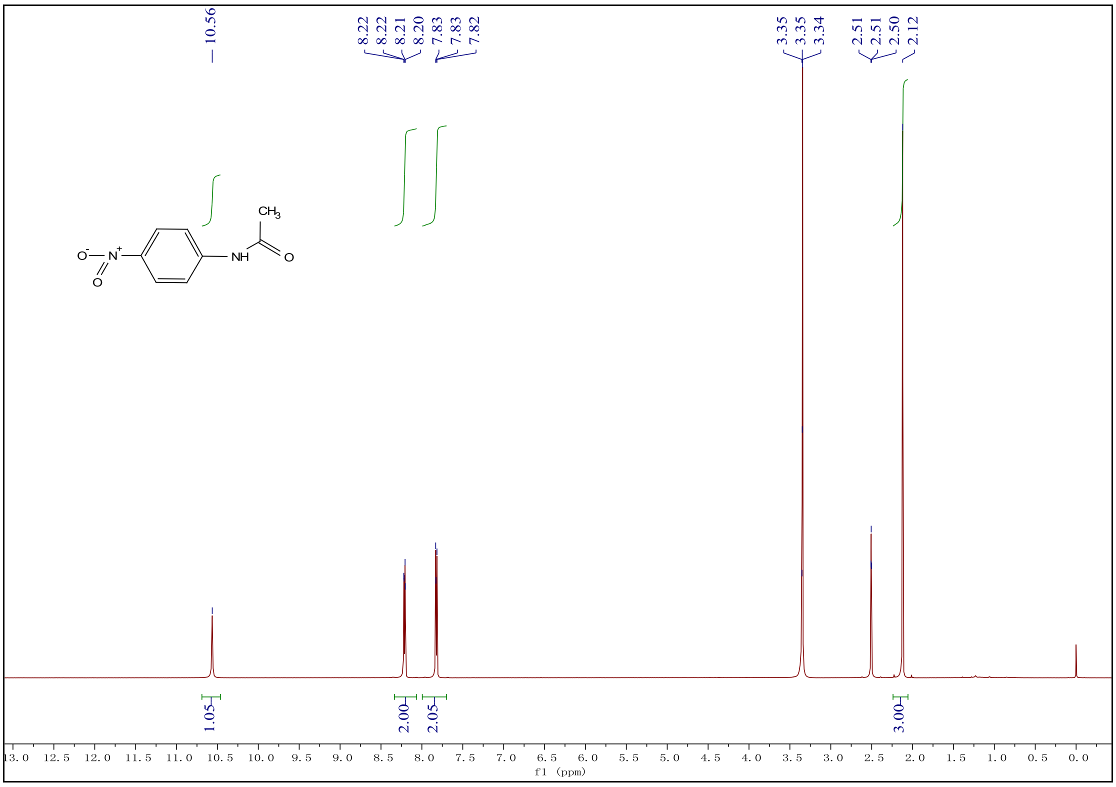

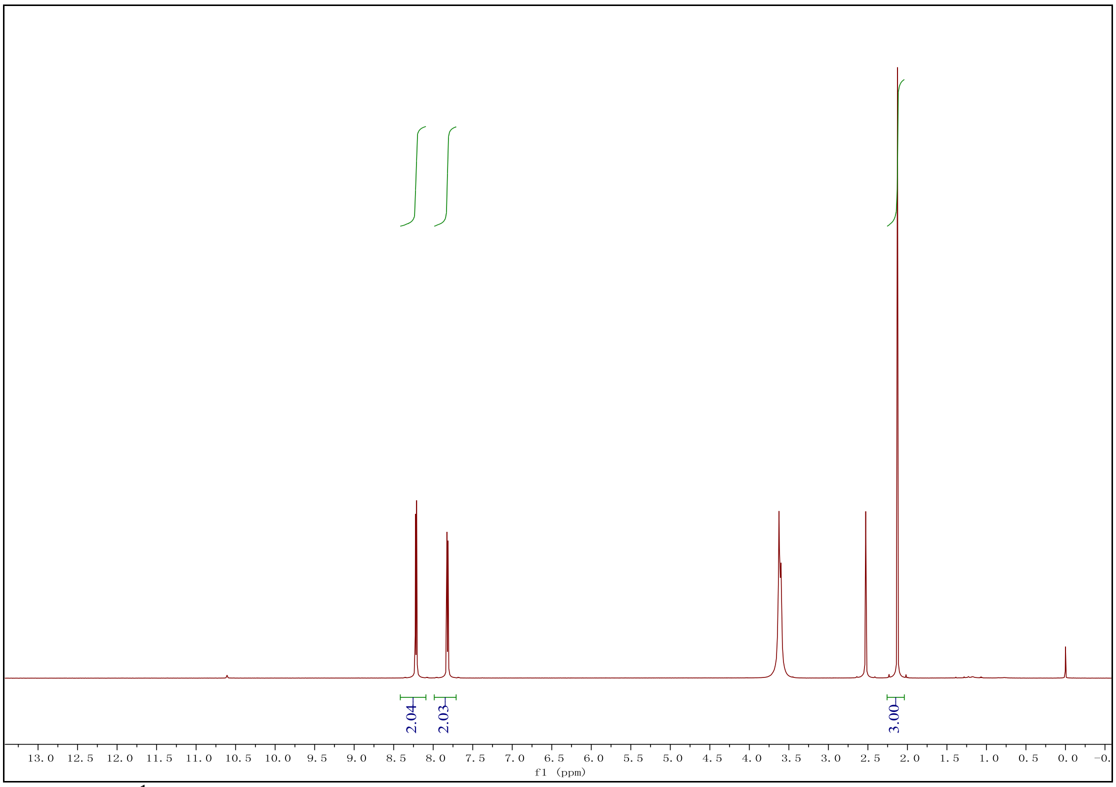

Figure 23. ^{1}H NMR spectrum of **1l** (Solvent: DMSO-d6)

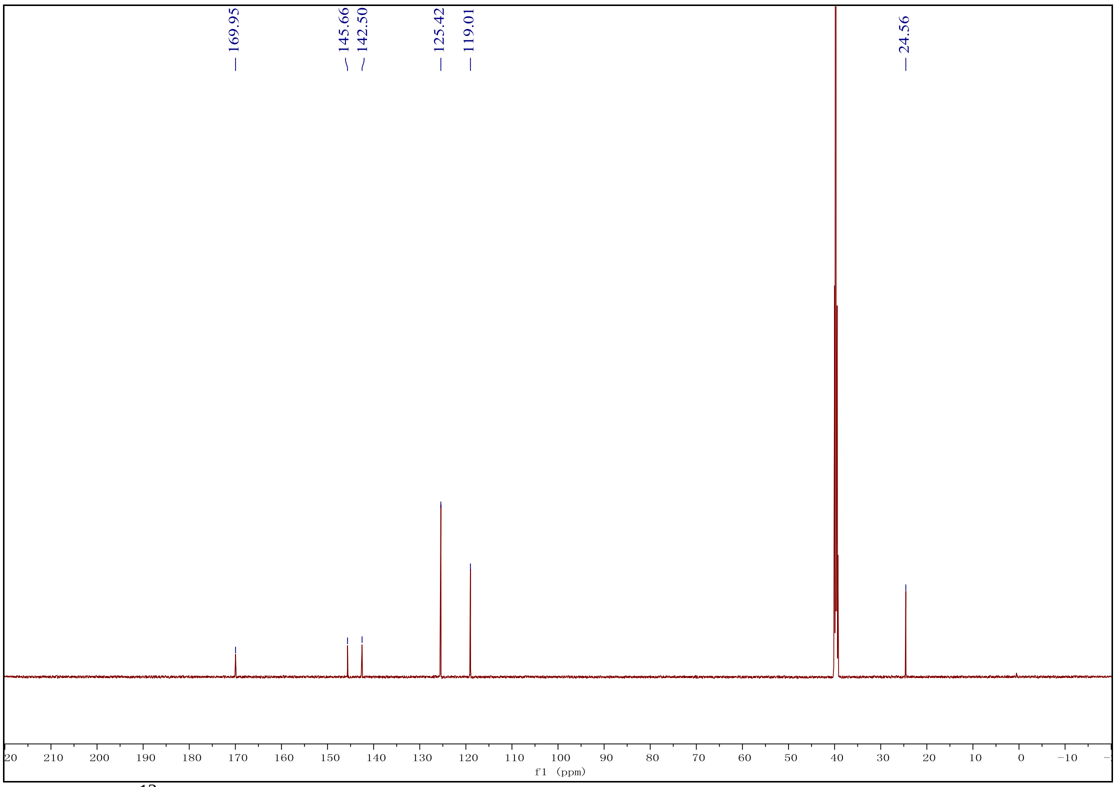

Figure 24. ^{13}C NMR spectrum of **1l** (Solvent: DMSO-d6)

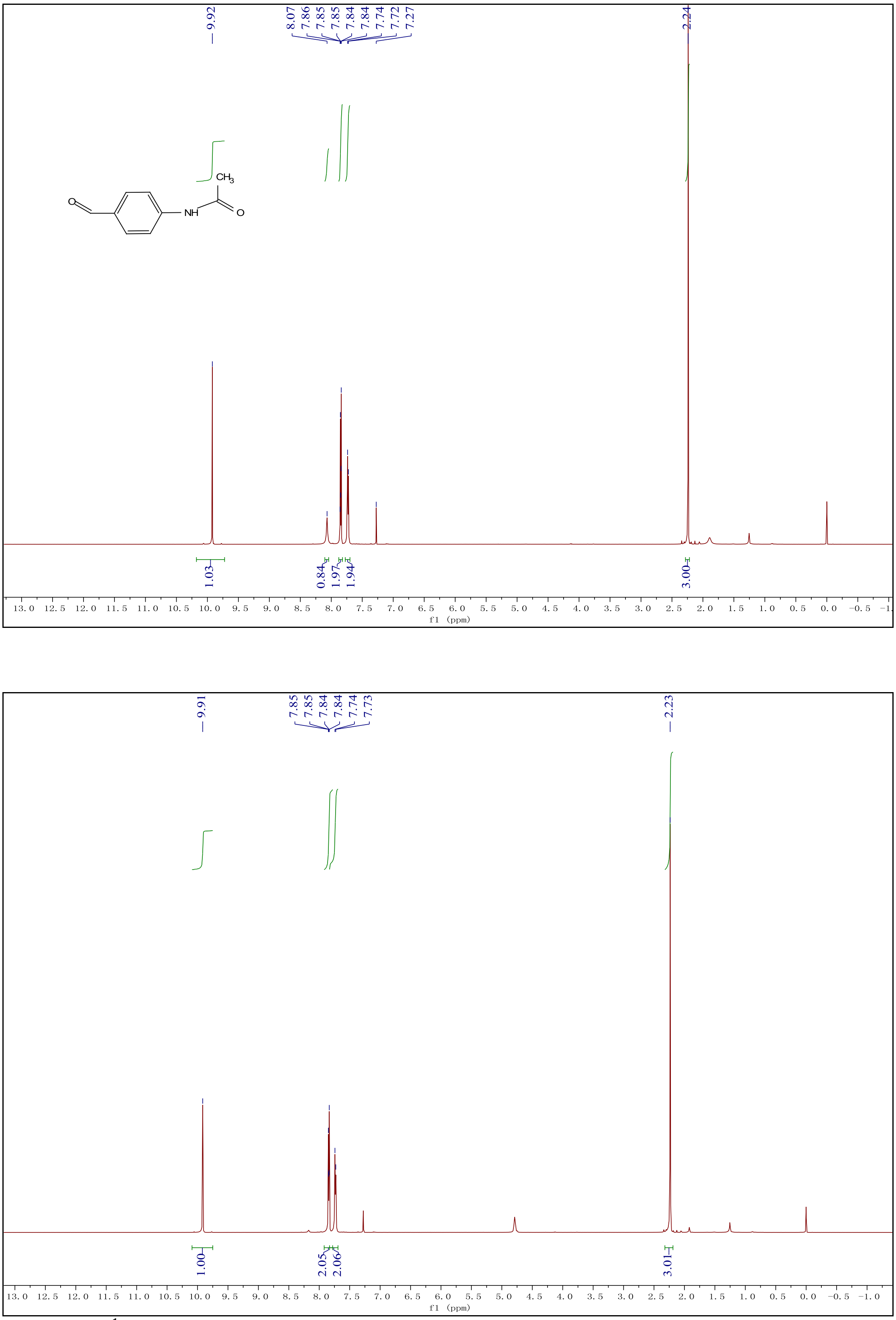

Figure 25. ^{1}H NMR spectrum of **1m** (Solvent: $CDCl_3$)

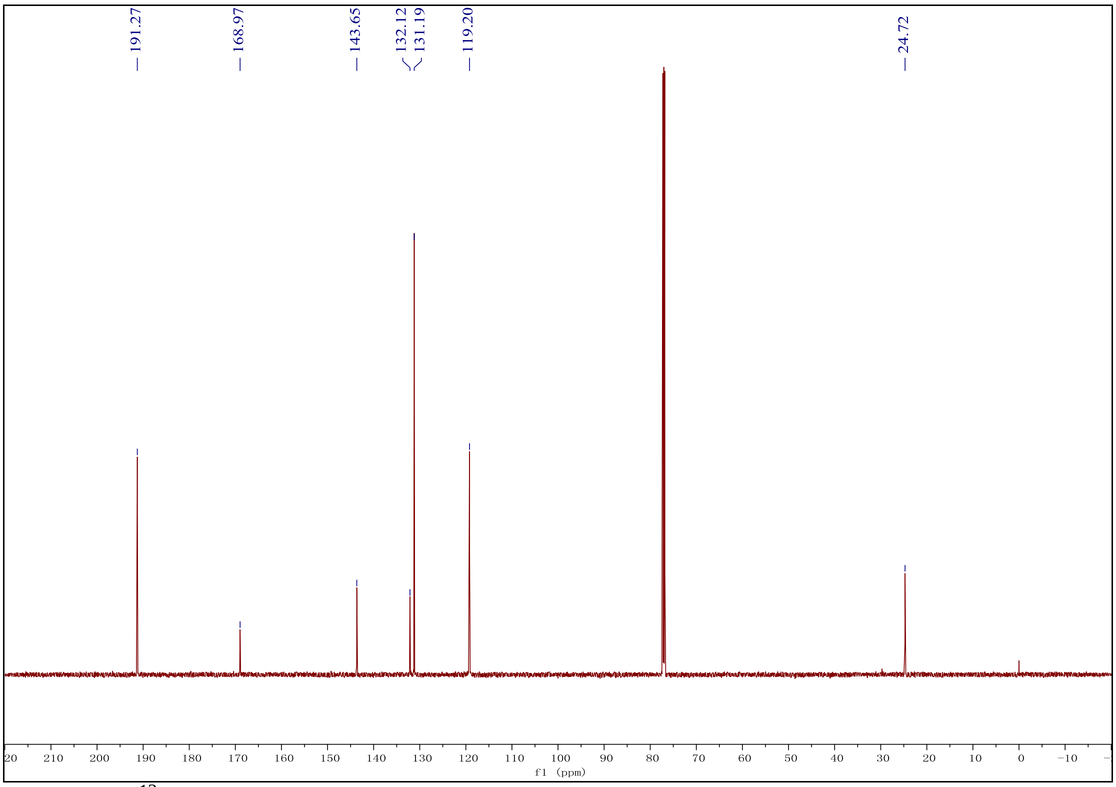

Figure 26. ^{13}C NMR spectrum of **1m** (Solvent: $CDCl_3$)

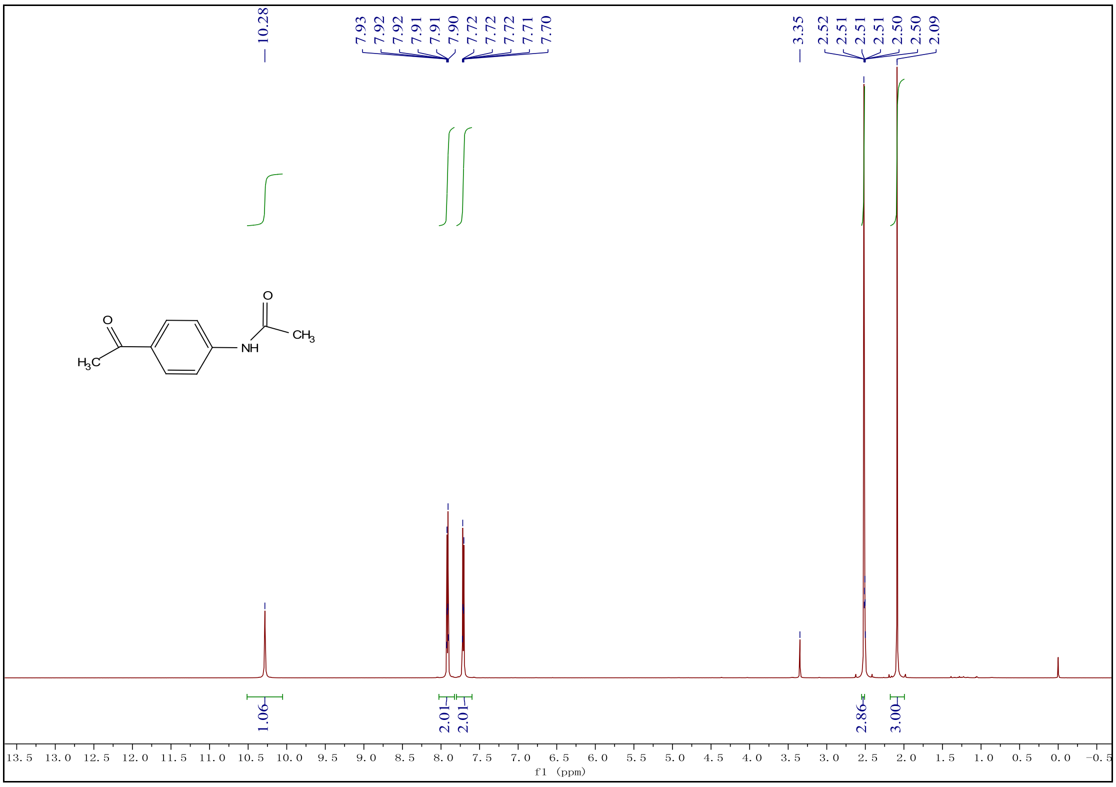

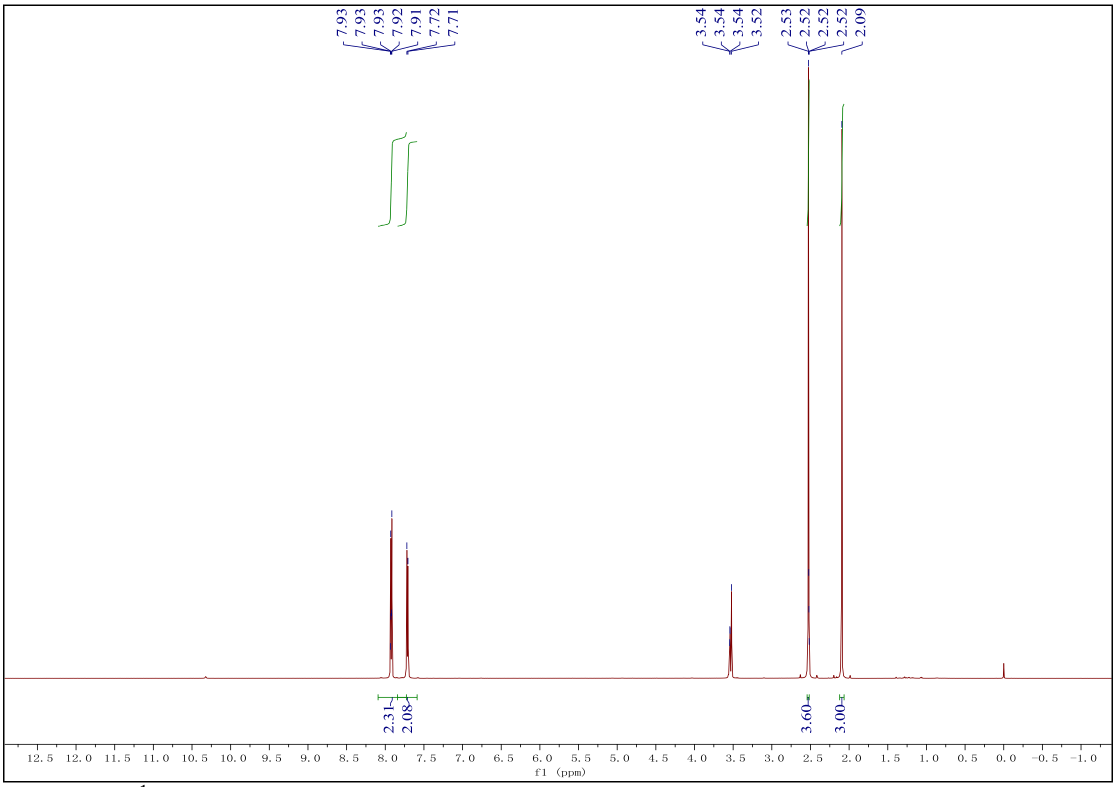

Figure 27. ^{1}H NMR spectrum of **1n** (Solvent: DMSO-d6)

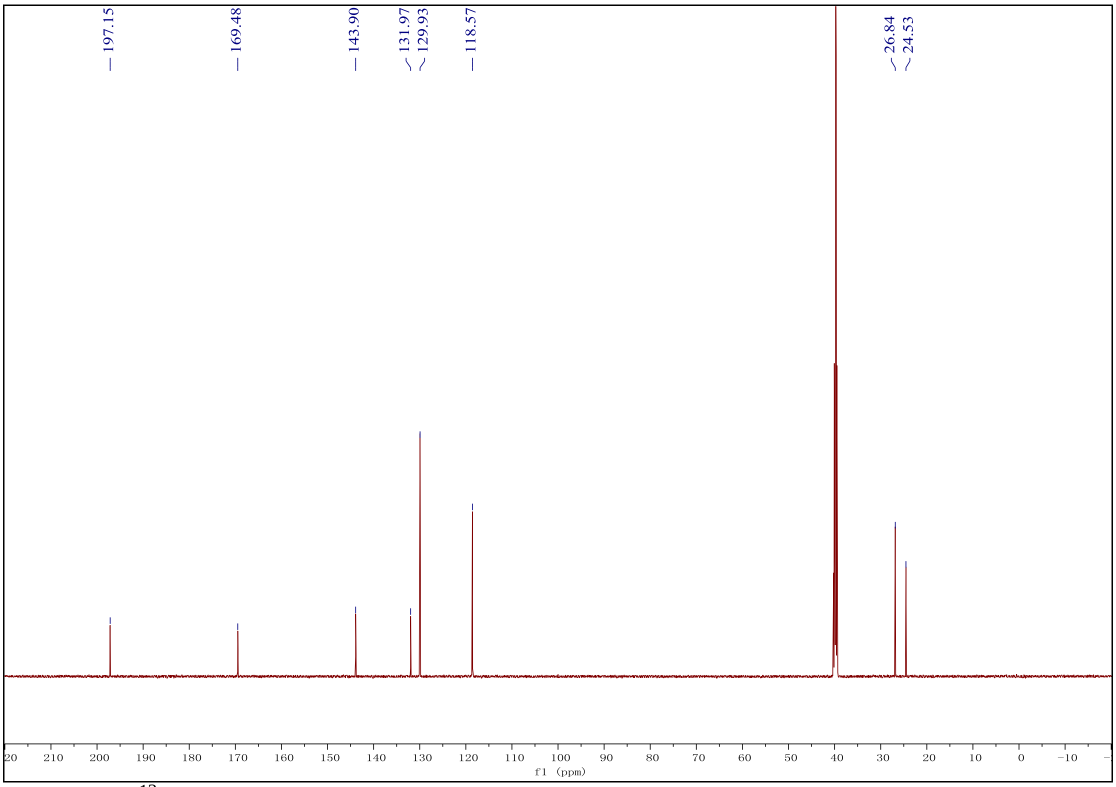

Figure 28. ^{13}C NMR spectrum of **1n** (Solvent: DMSO-d6)

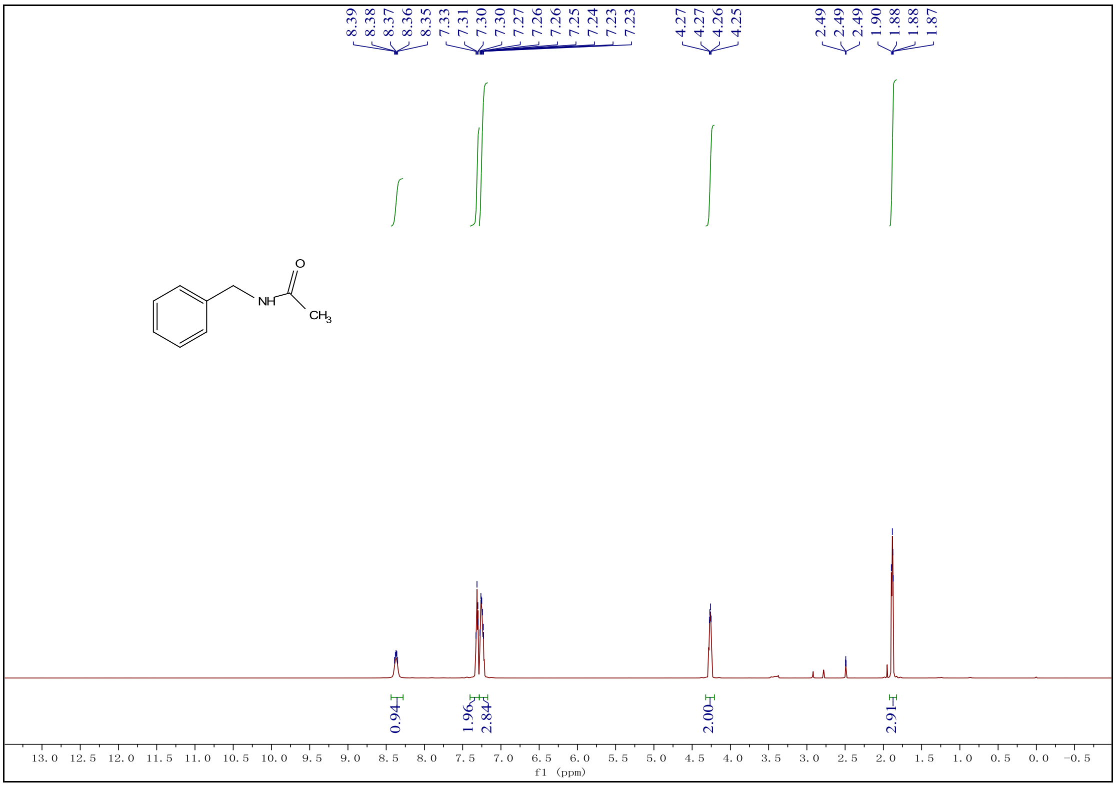

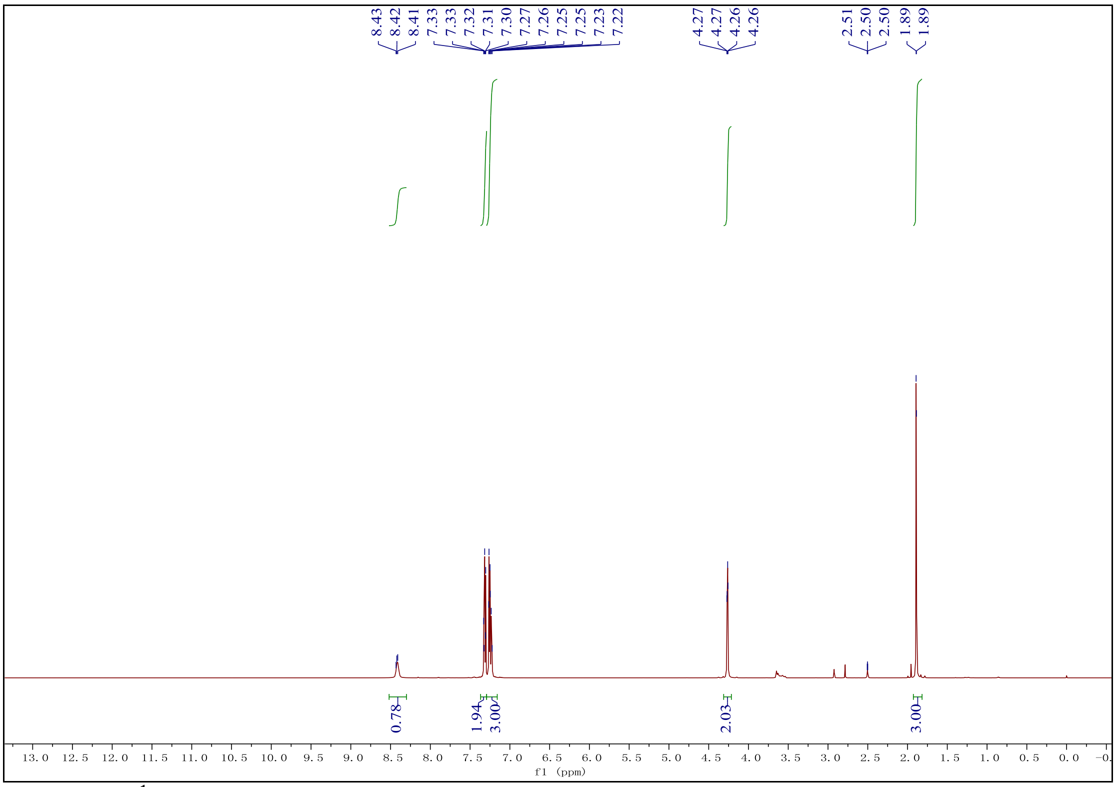

Figure 29. ^{1}H NMR spectrum of **1o** (Solvent: DMSO-d6)

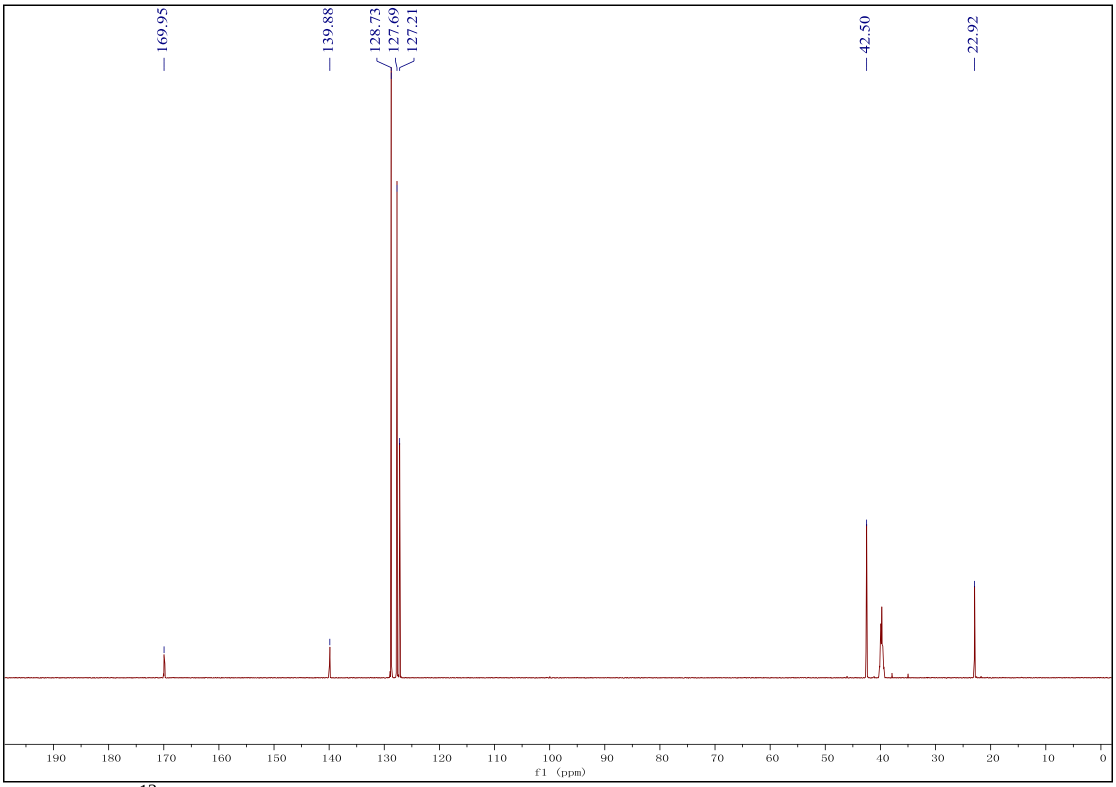

Figure 30. ^{13}C NMR spectrum of **1o** (Solvent: DMSO-d6)

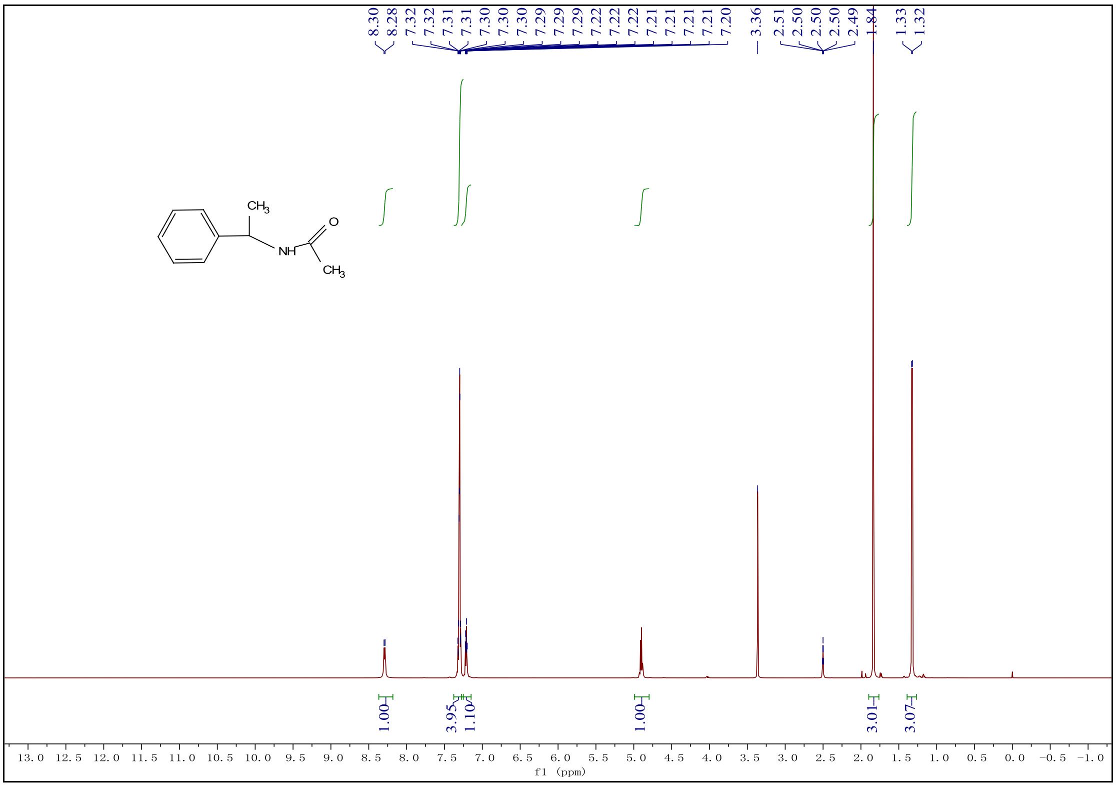

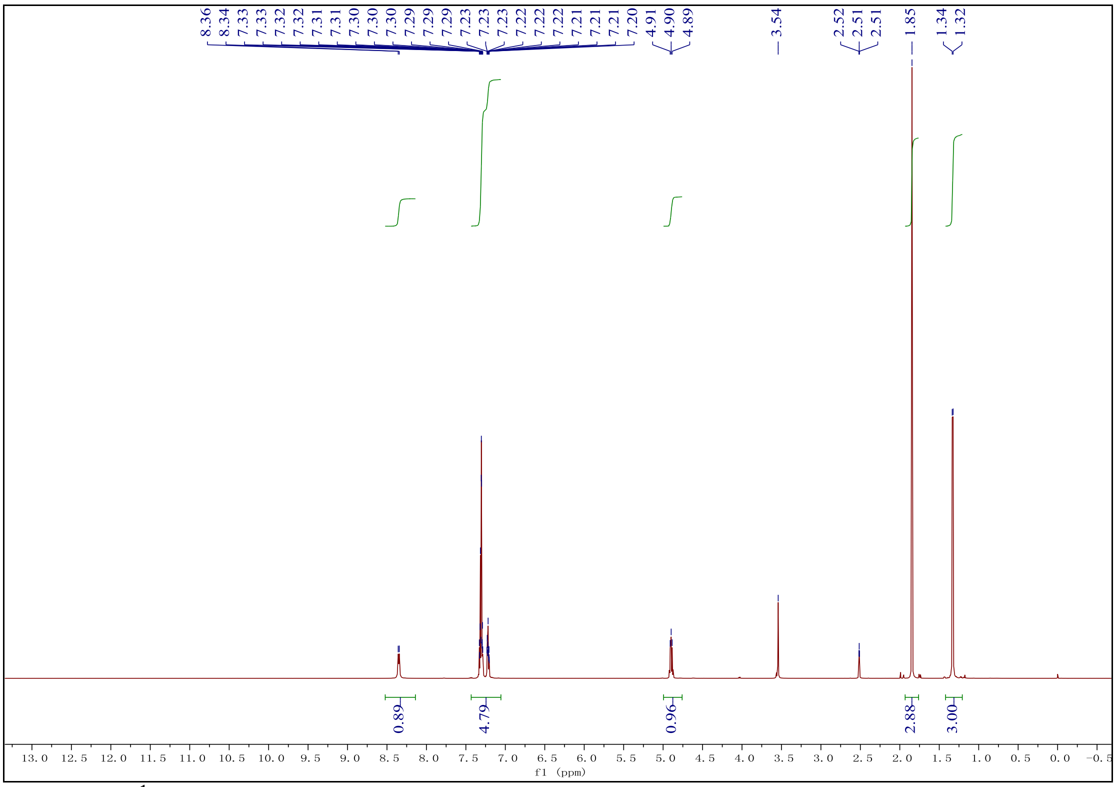

Figure 31. ^{1}H NMR spectrum of **1p** (Solvent: DMSO-d6)

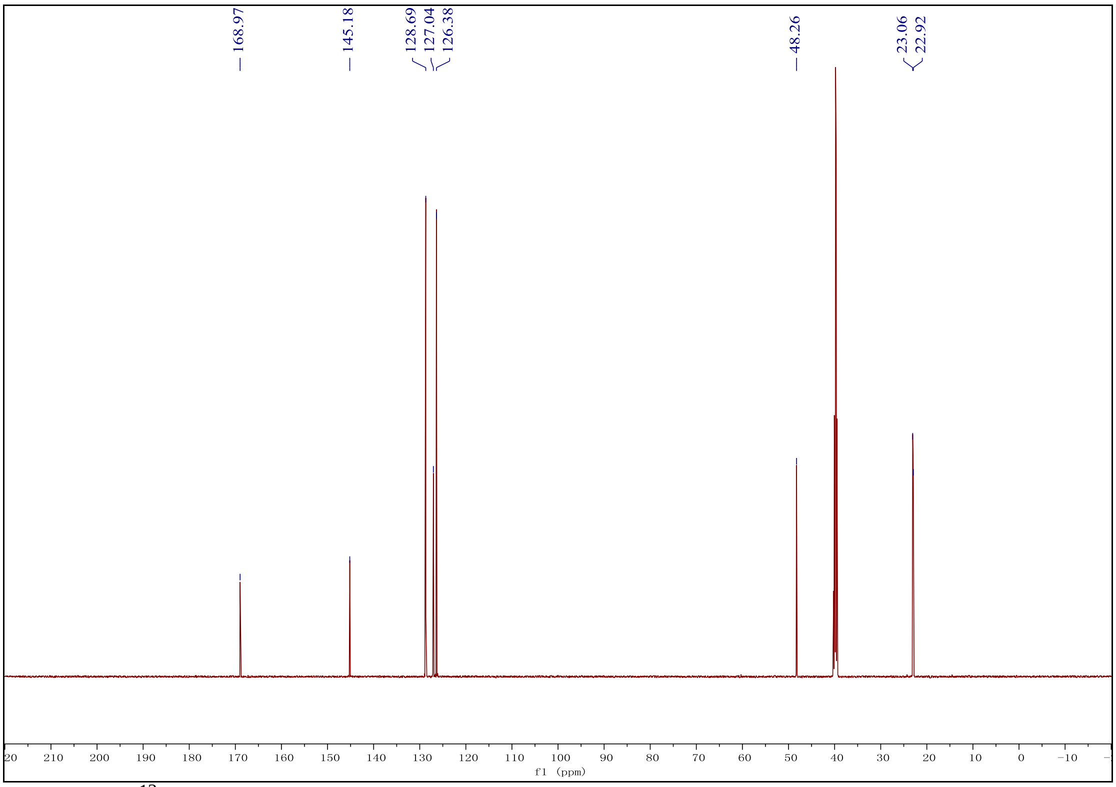

Figure 32. ^{13}C NMR spectrum of **1p** (Solvent: DMSO-d6)

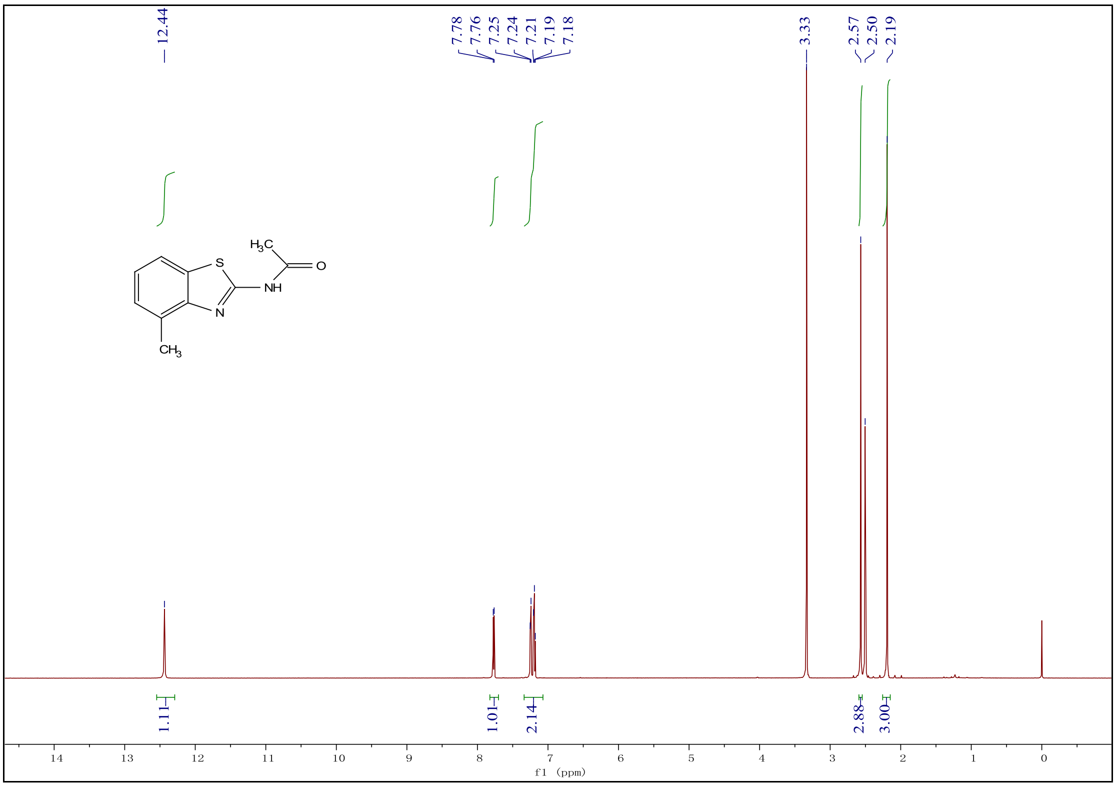

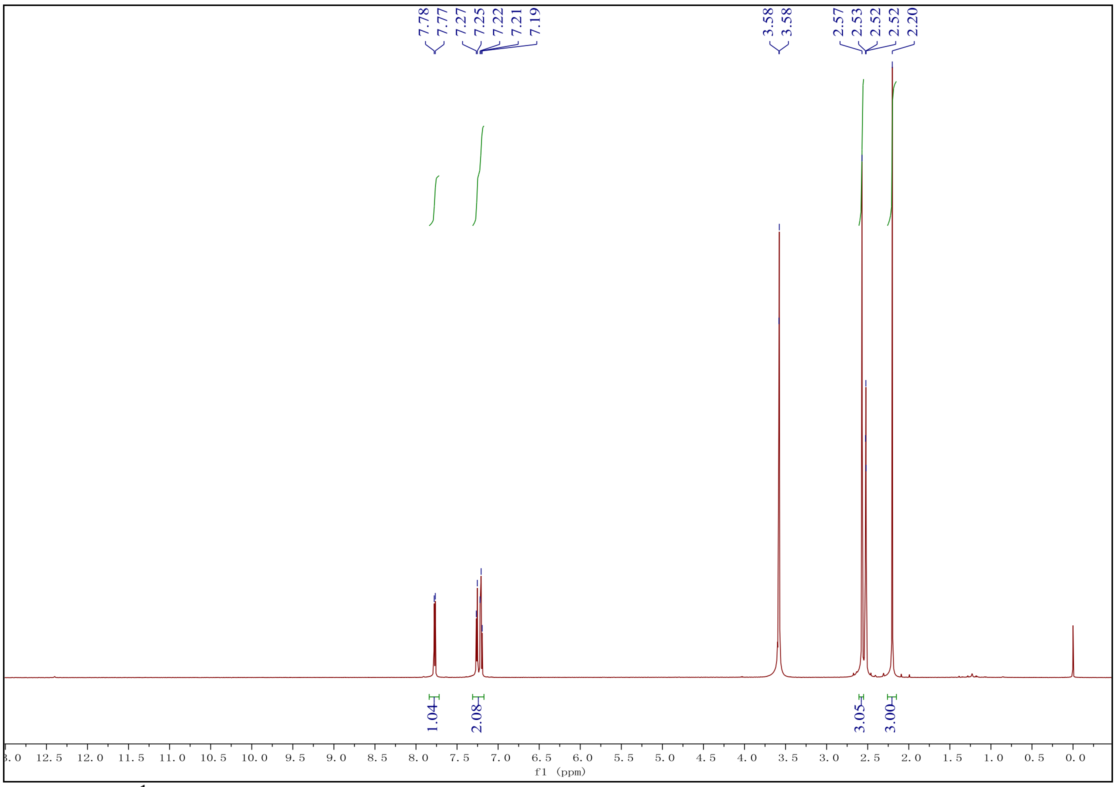

Figure 33. ^{1}H NMR spectrum of **1q** (Solvent: DMSO-d6)

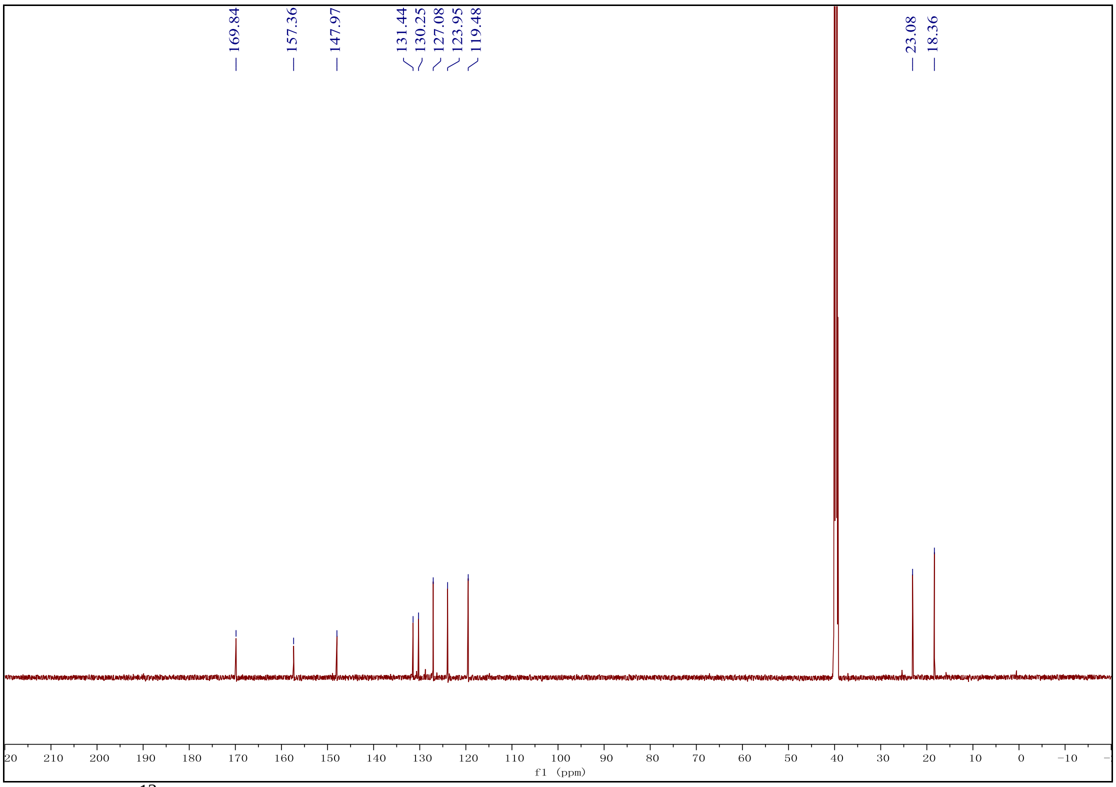

Figure 34. ^{13}C NMR spectrum of **1q** (Solvent: DMSO-d6)

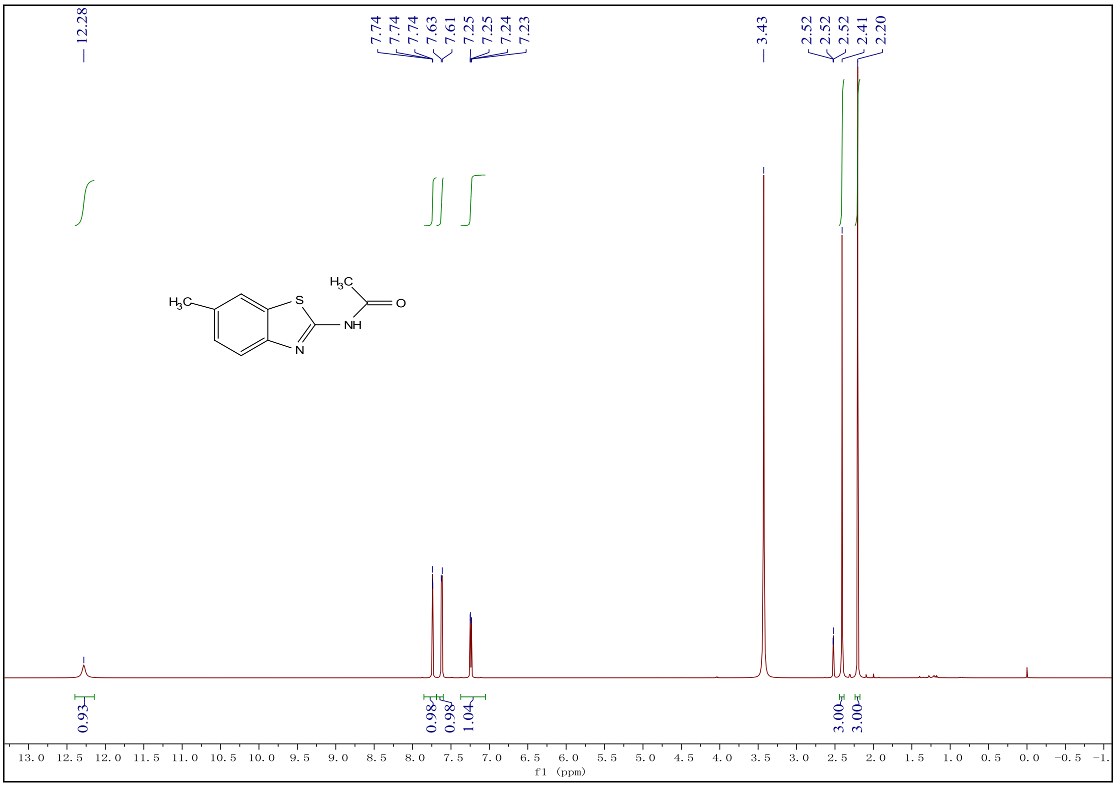

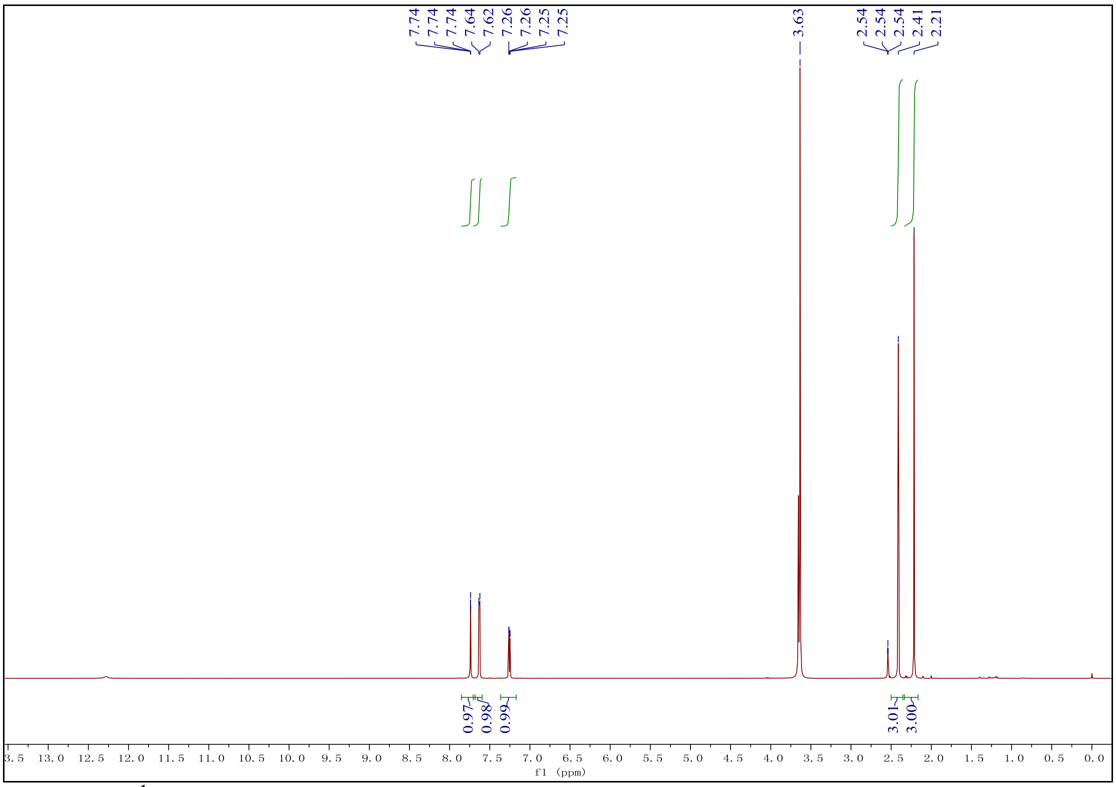

Figure 35. ^{1}H NMR spectrum of **1r** (Solvent: DMSO-d6)

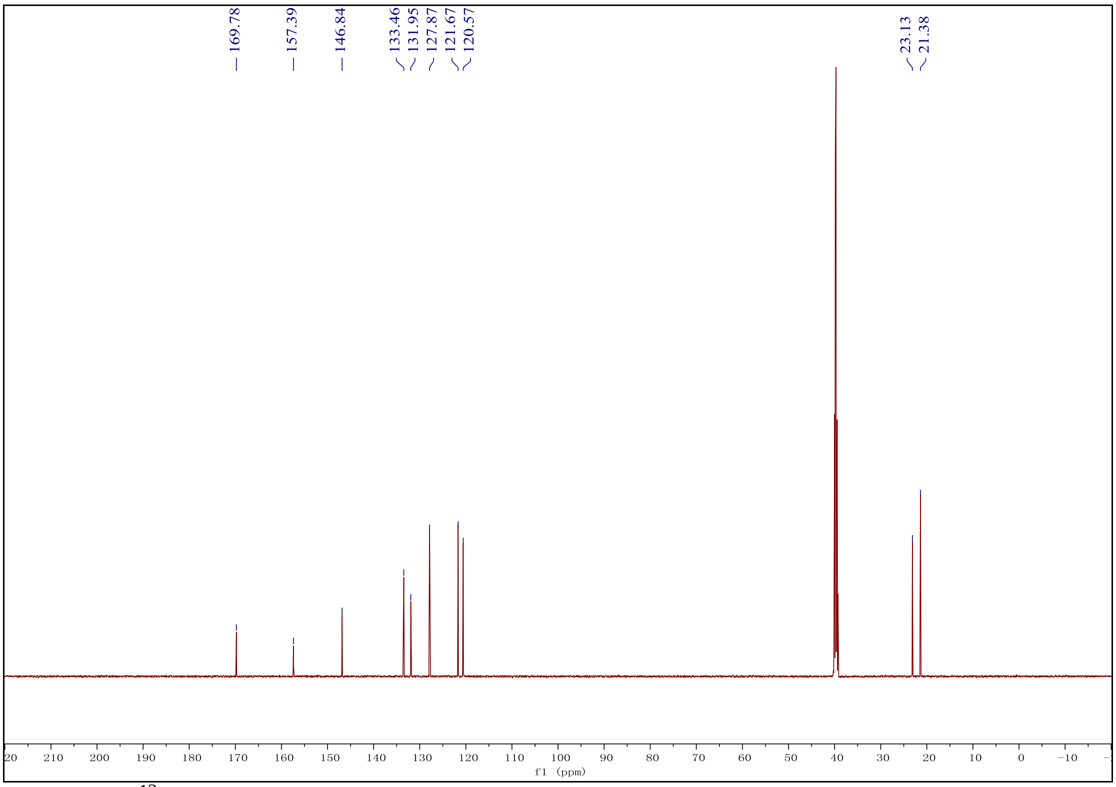

Figure 36. ^{13}C NMR spectrum of **1r** (Solvent: DMSO-d6)

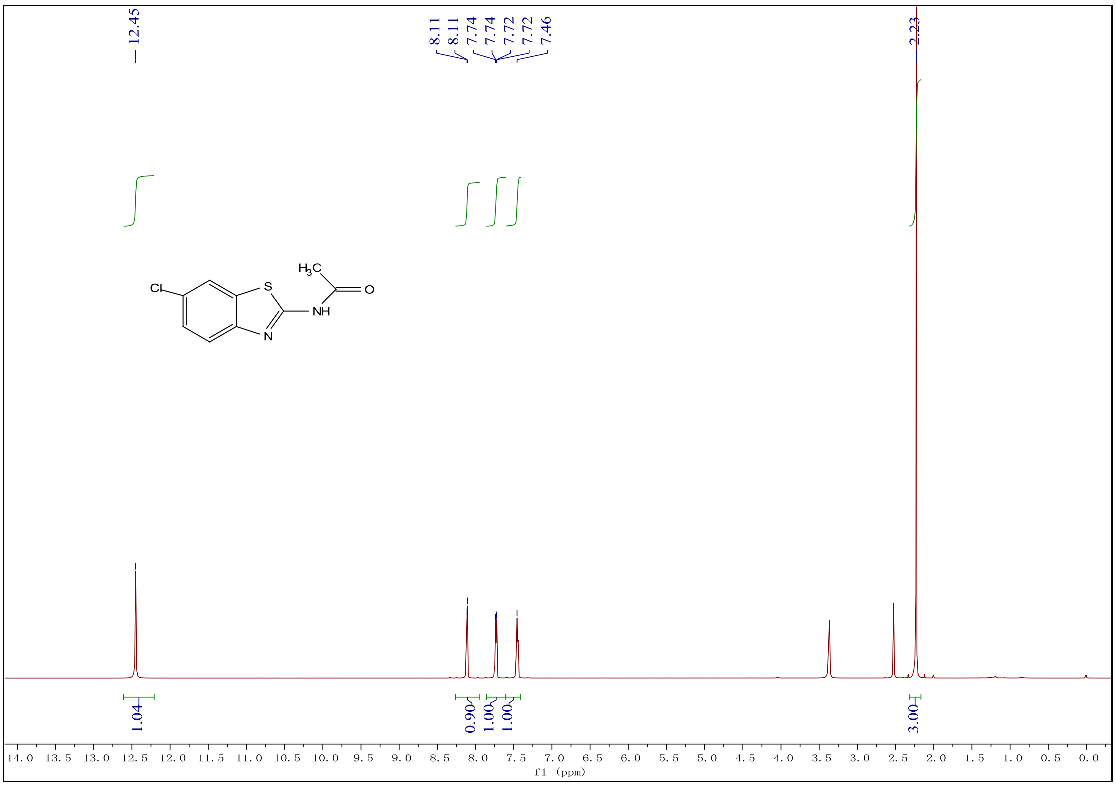

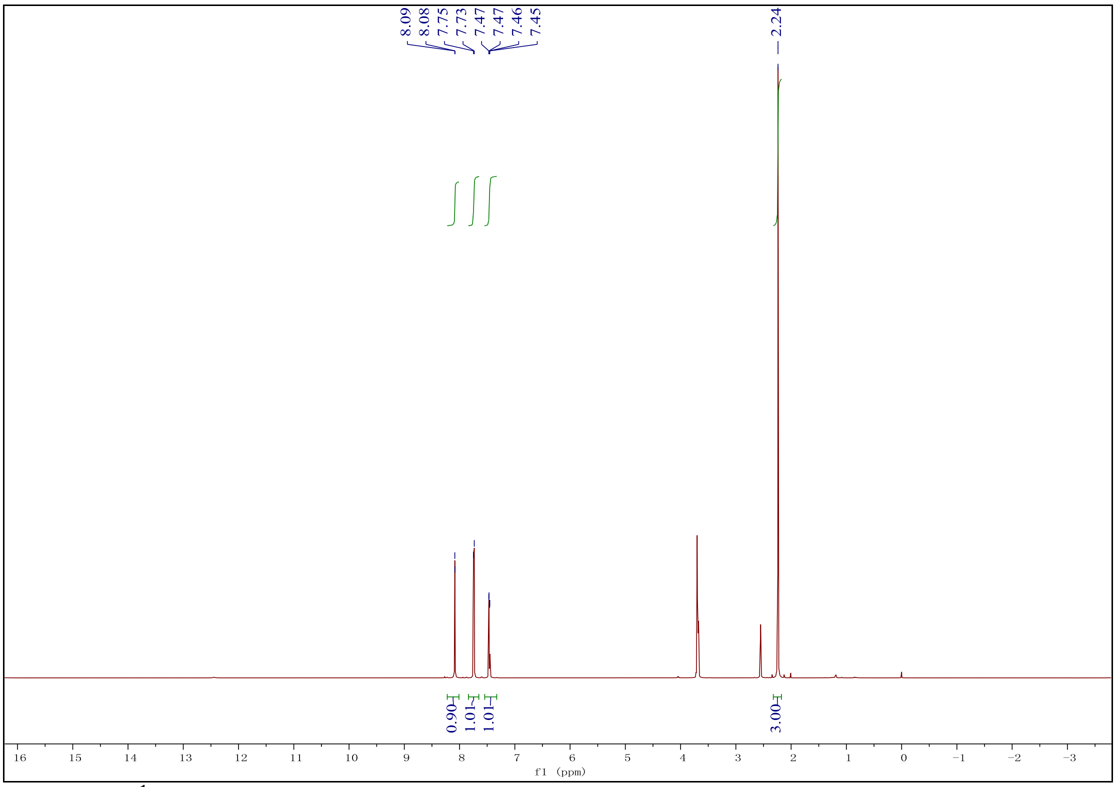

Figure 37. ^{1}H NMR spectrum of **1s**(Solvent: DMSO-d6)

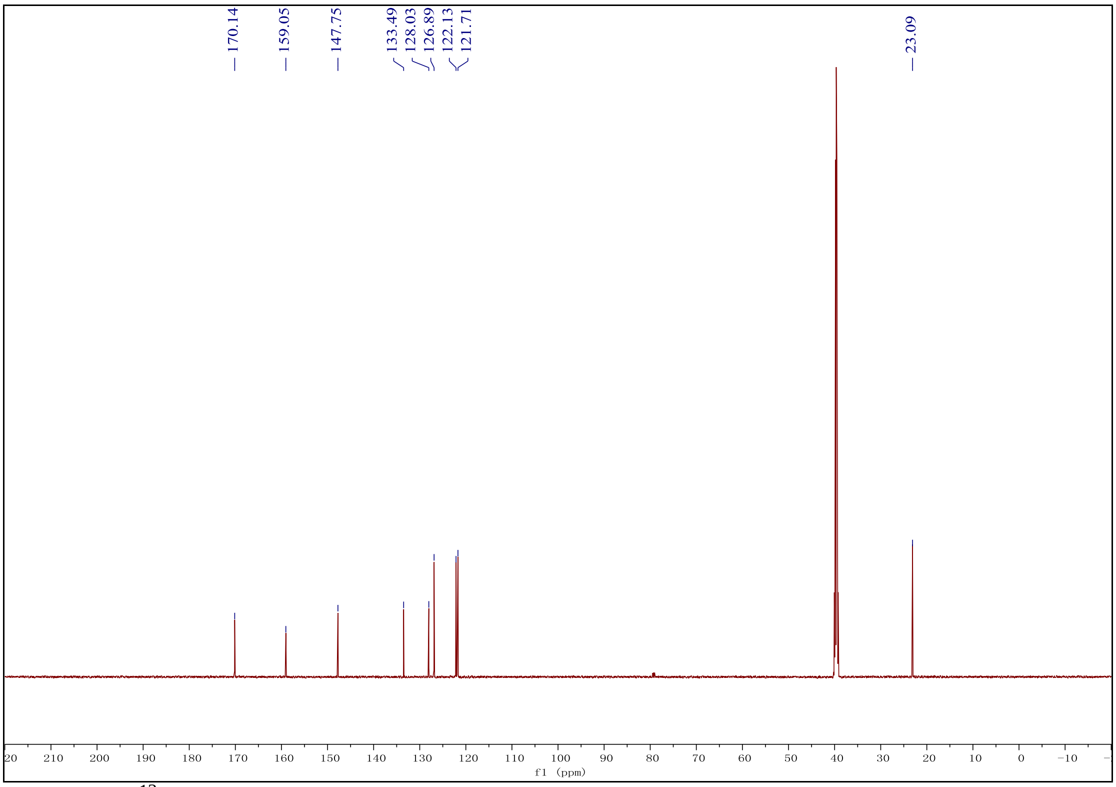

Figure 38. ^{13}C NMR spectrum of **1s** (Solvent: DMSO-d6)

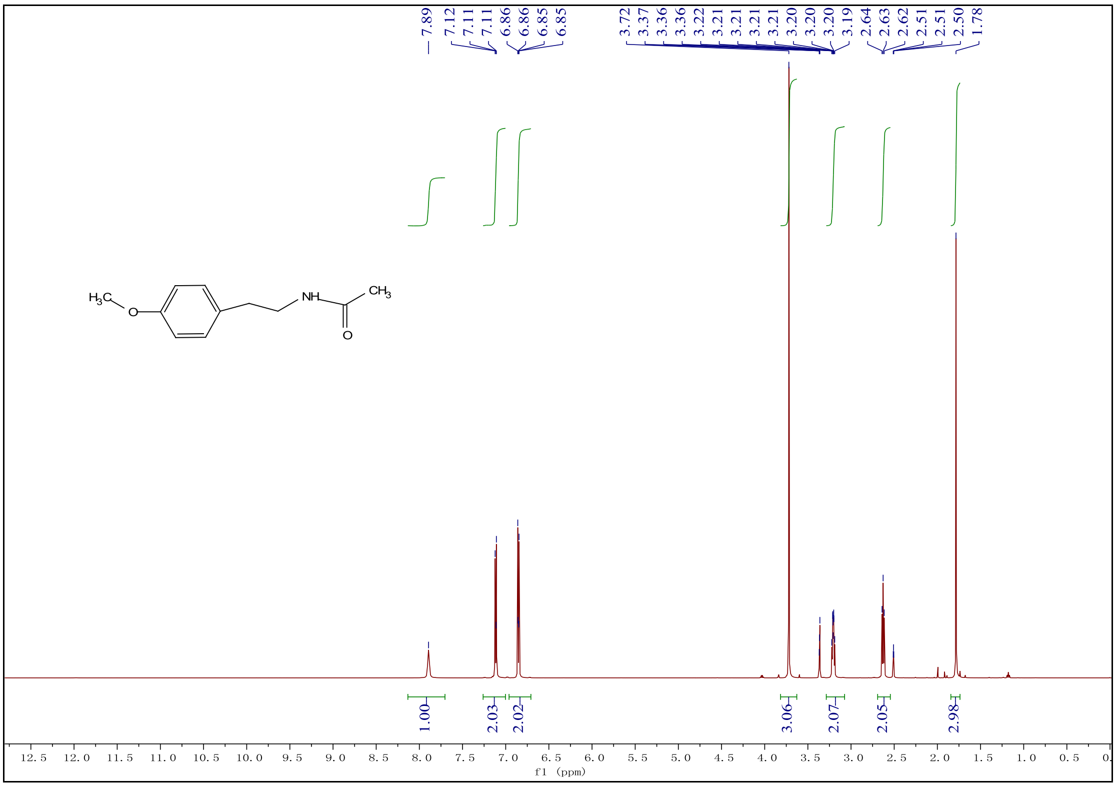

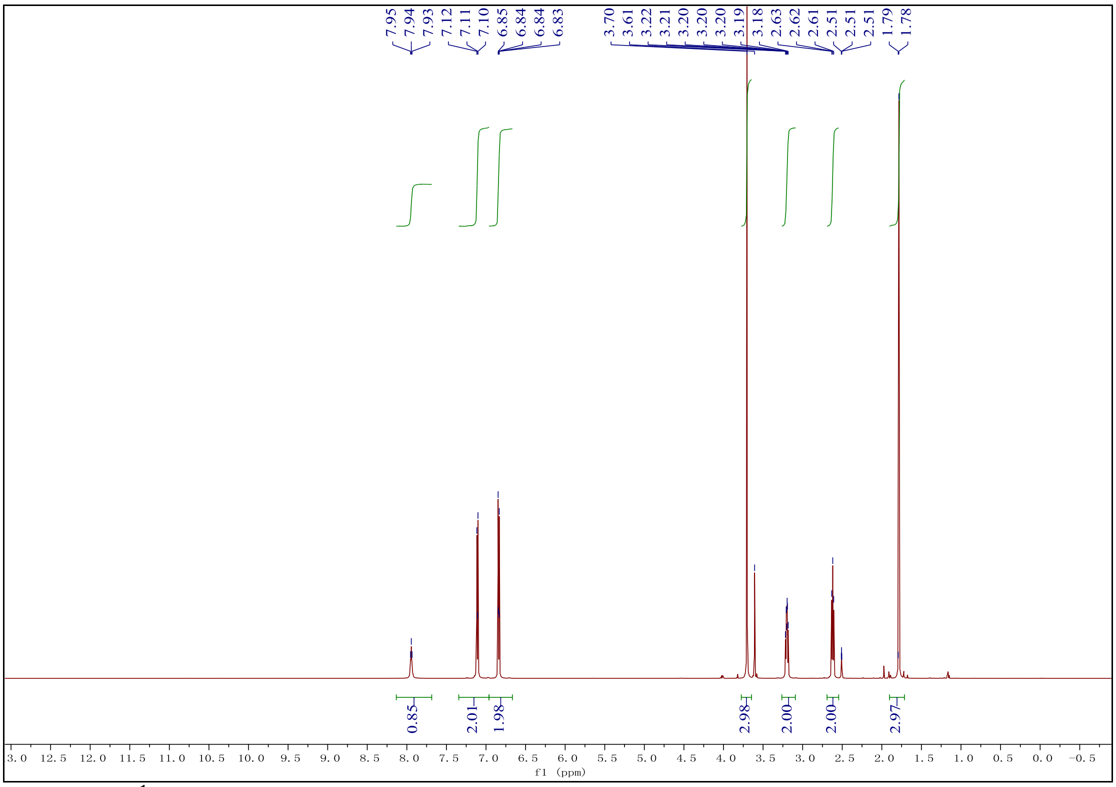

Figure 39. ^{1}H NMR spectrum of **1t** (Solvent: DMSO-d6)

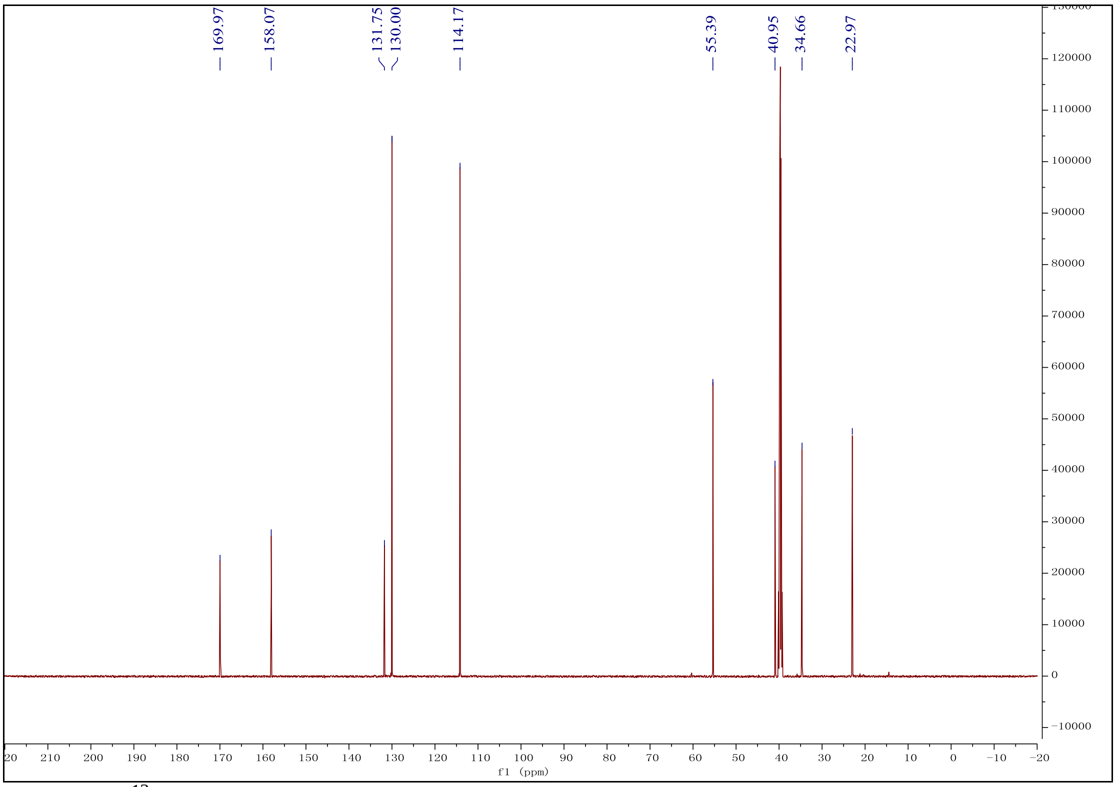

Figure40. ^{13}C NMR spectrum of **1t** (Solvent: DMSO-d6)

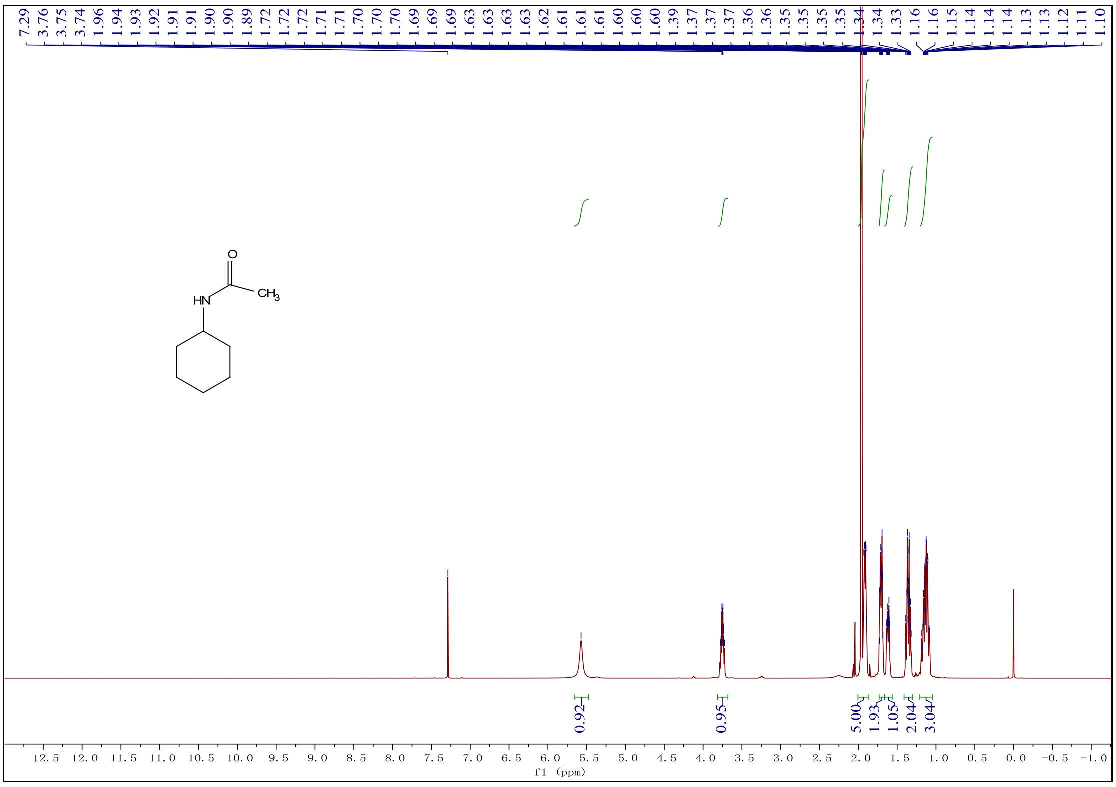

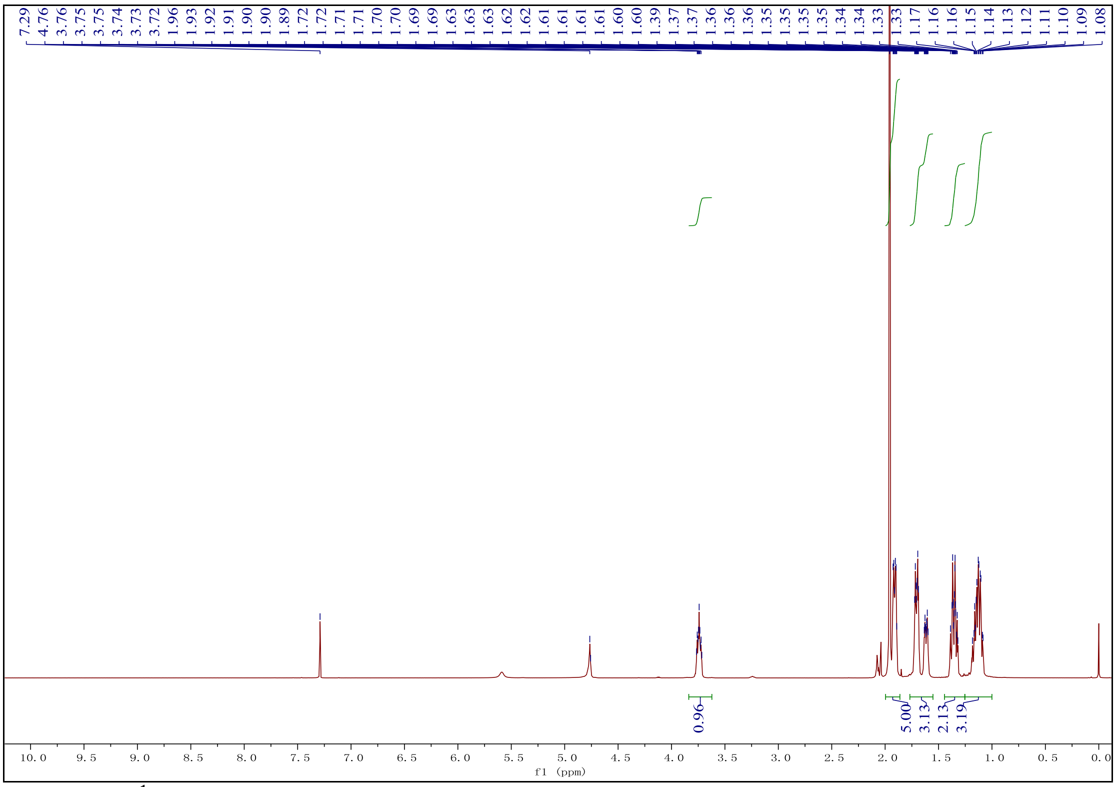

Figure 41. ^{1}H NMR spectrum of **1u** (Solvent: $CDCl_3$)

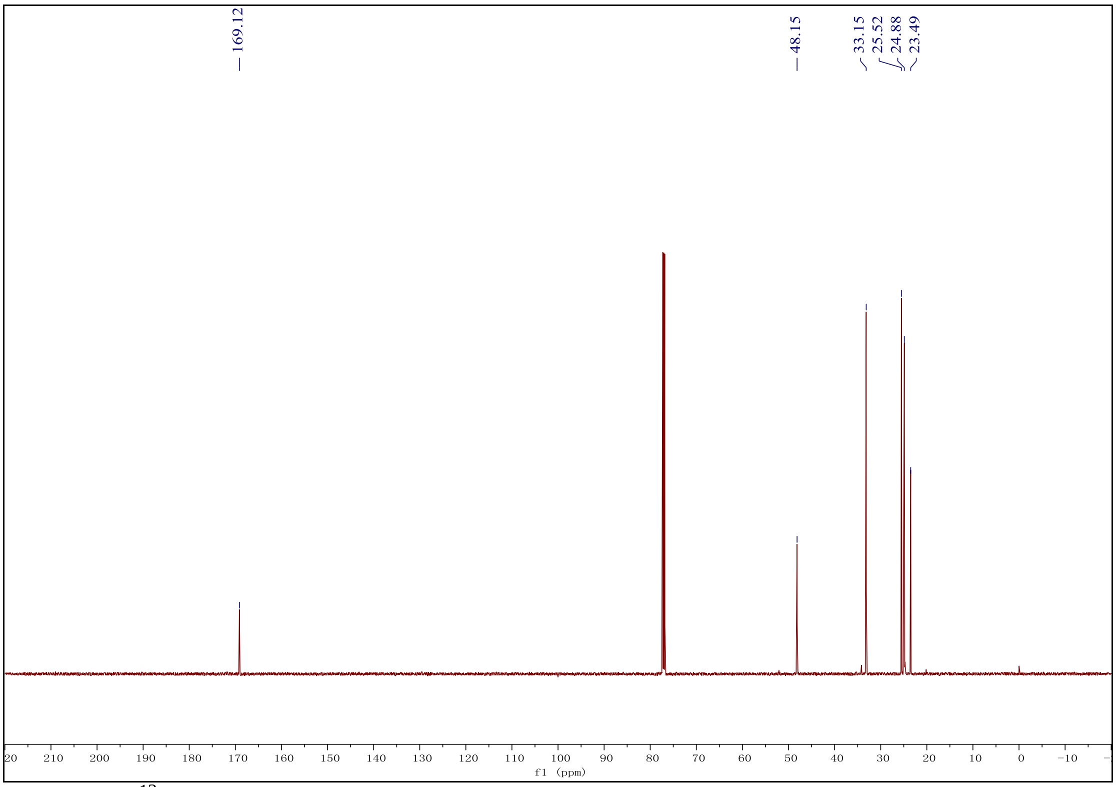

Figure 42. ^{13}C NMR spectrum of **1u** (Solvent: $CDCl_3$)

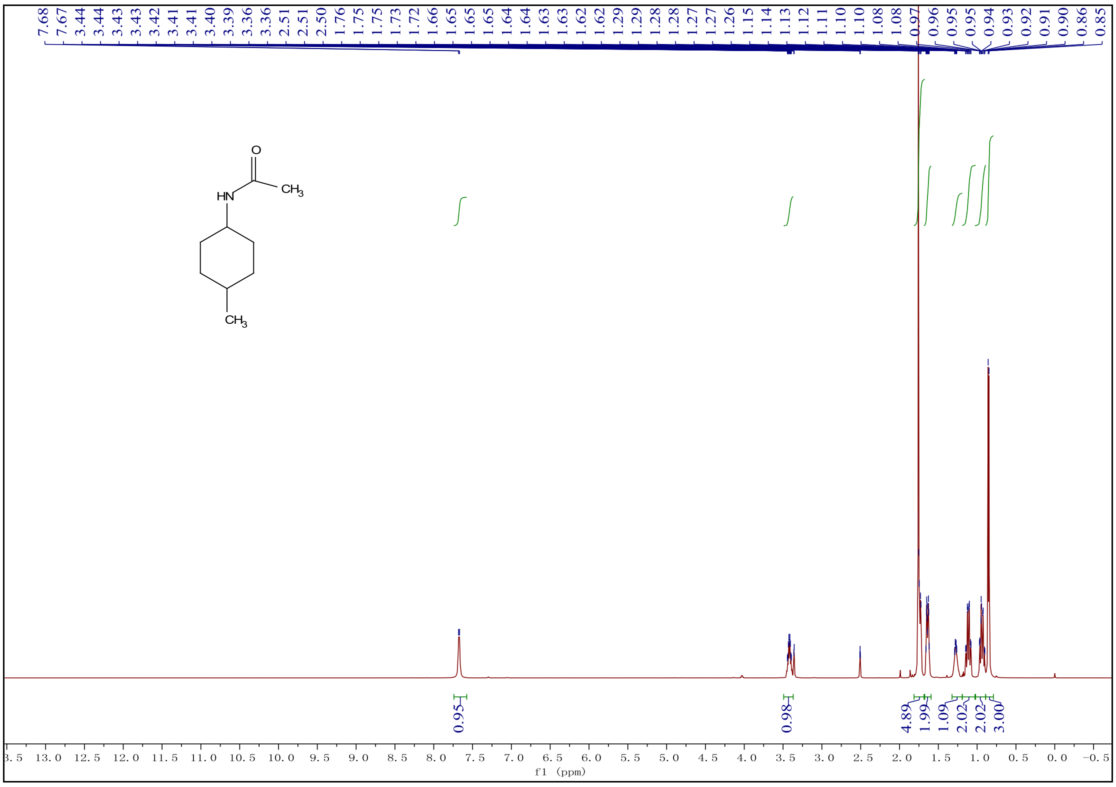

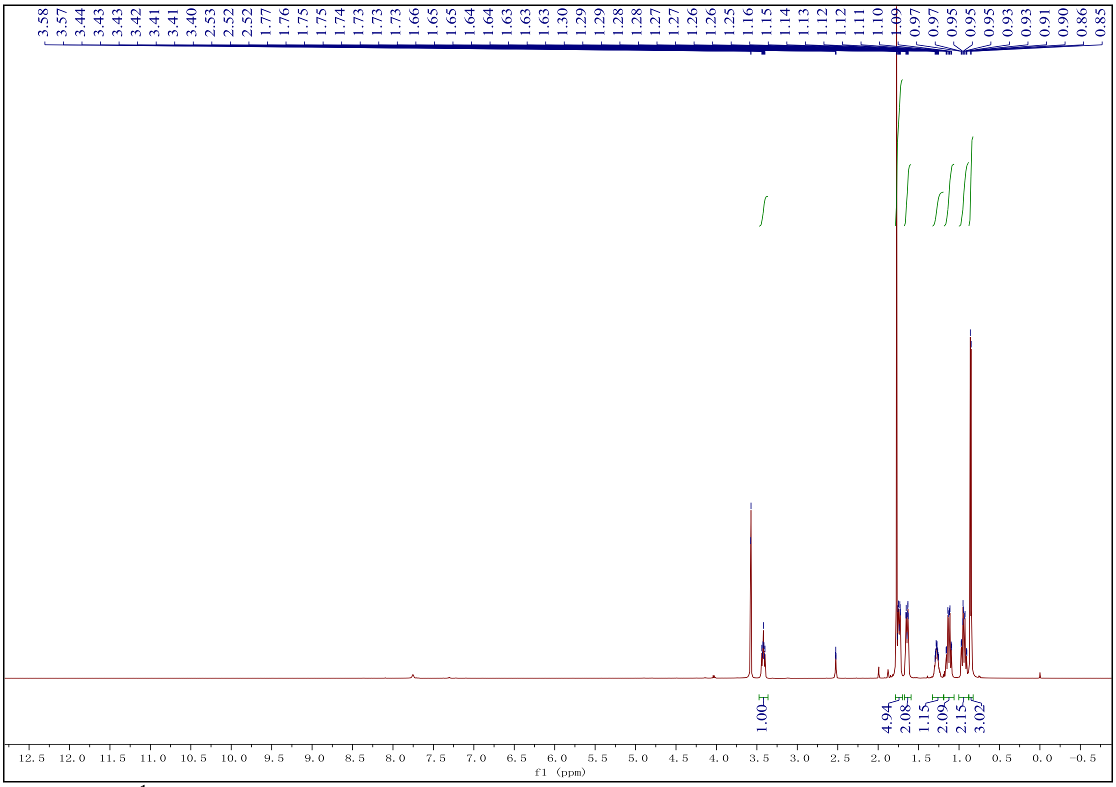

Figure 43. ^{1}H NMR spectrum of **1v** (Solvent: $CDCl_3$)

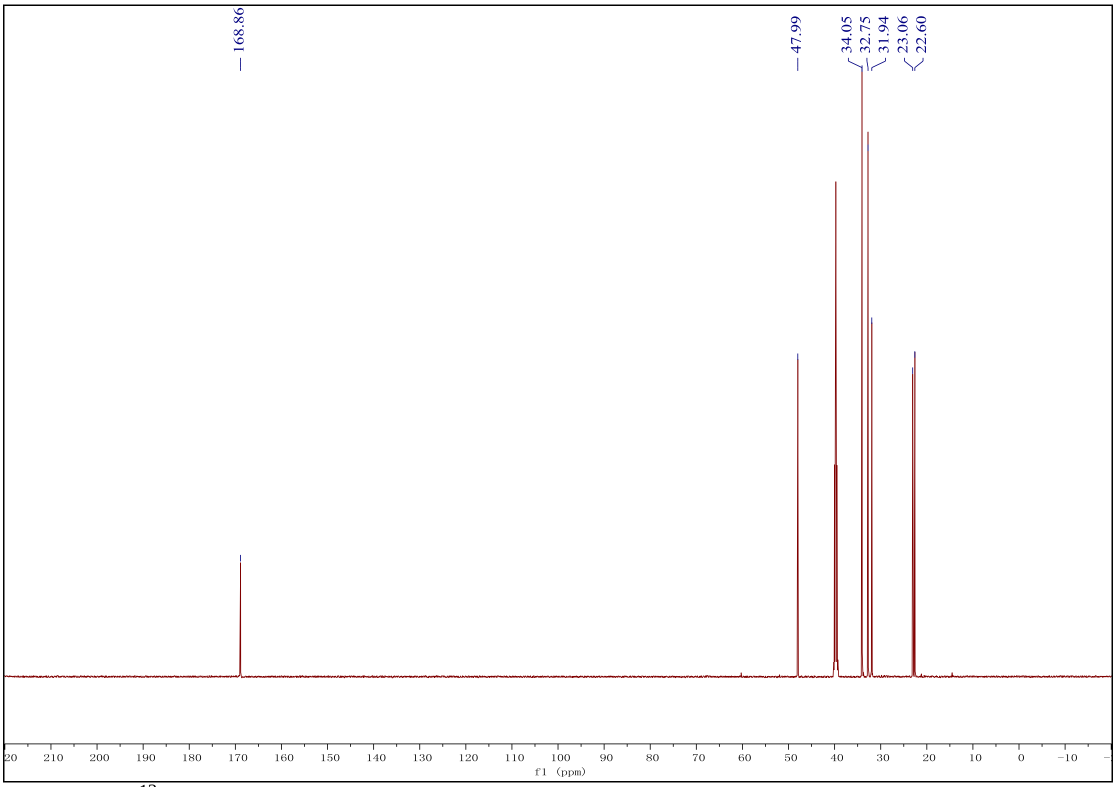

Figure 44. ^{13}C NMR spectrum of **1v** (Solvent: $CDCl_3$)

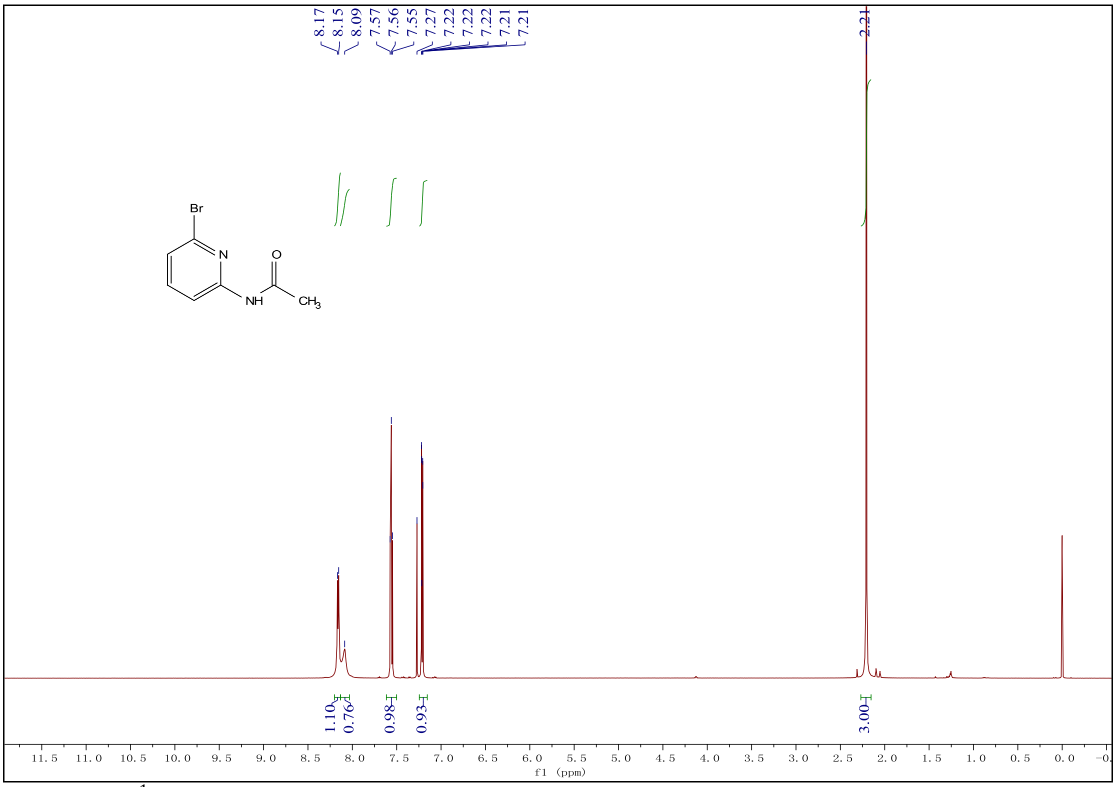

Figure 45. ^{1}H NMR spectrum of **1w** (Solvent: $CDCl_3$)

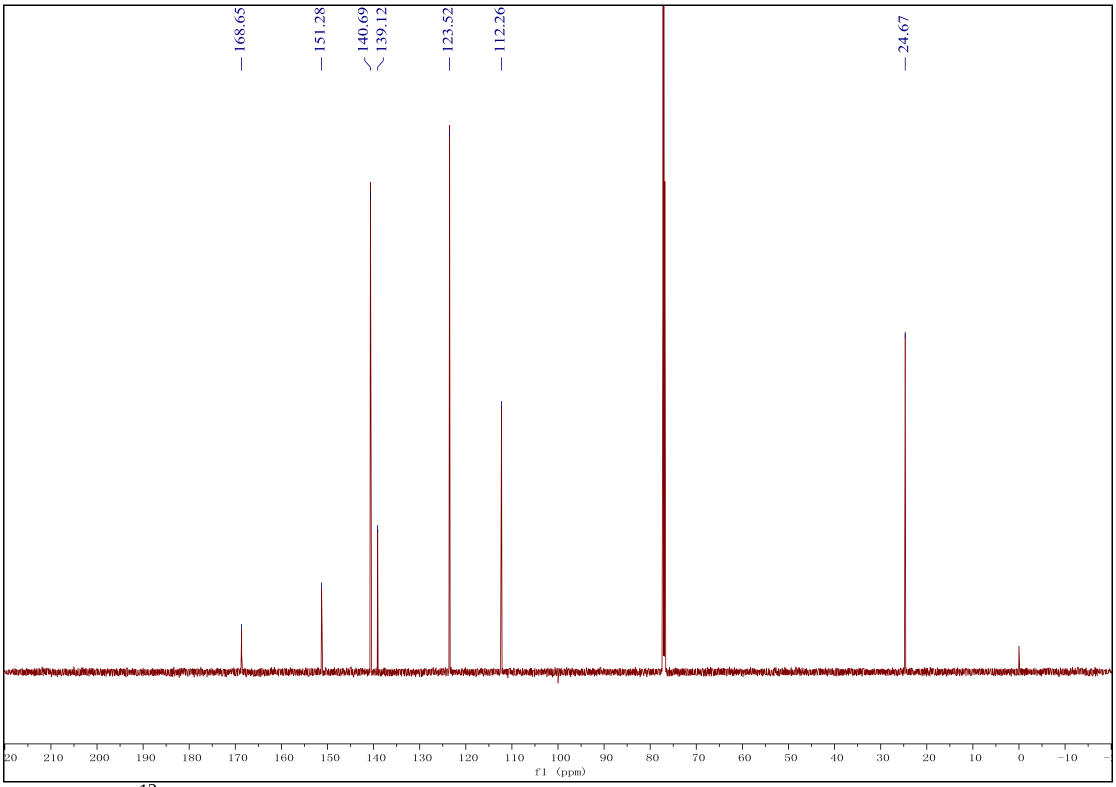

Figure 46. ^{13}C NMR spectrum of **1w** (Solvent: $CDCl_3$)

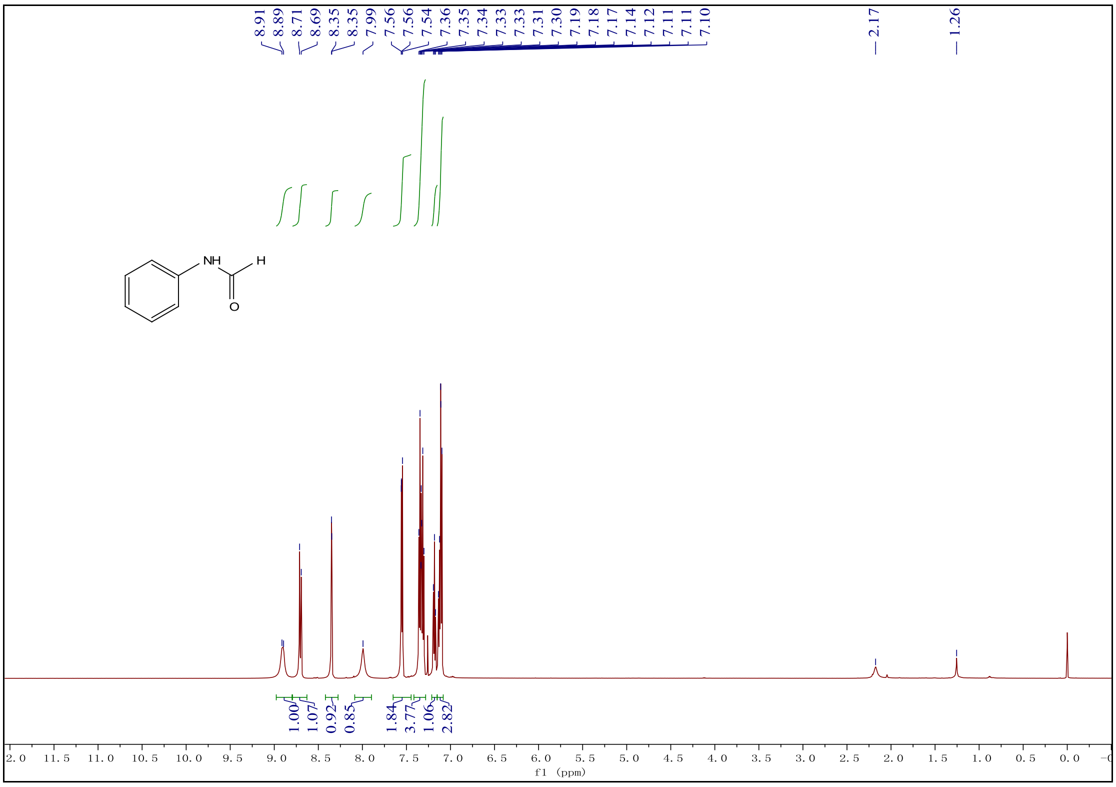

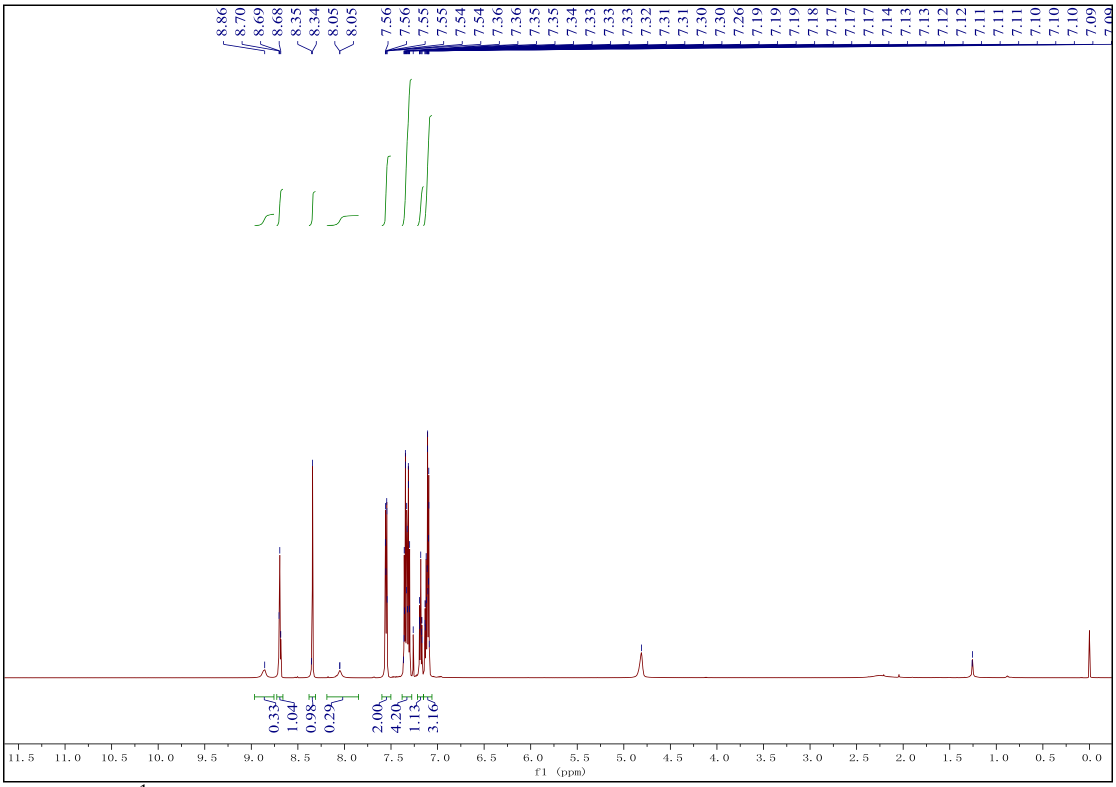

Figure 47. ^{1}H NMR spectrum of **2a** (Solvent: $CDCl_3$)

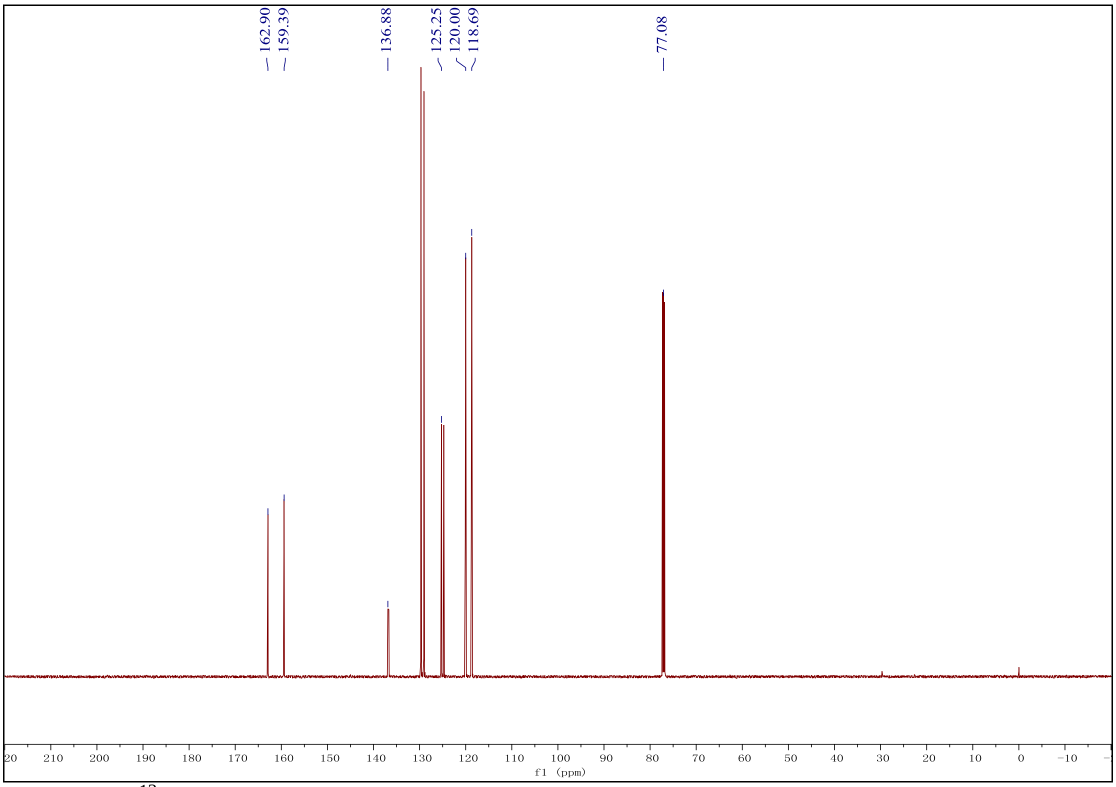

Figure 48. ^{13}C NMR spectrum of **2a** (Solvent: $CDCl_3$)

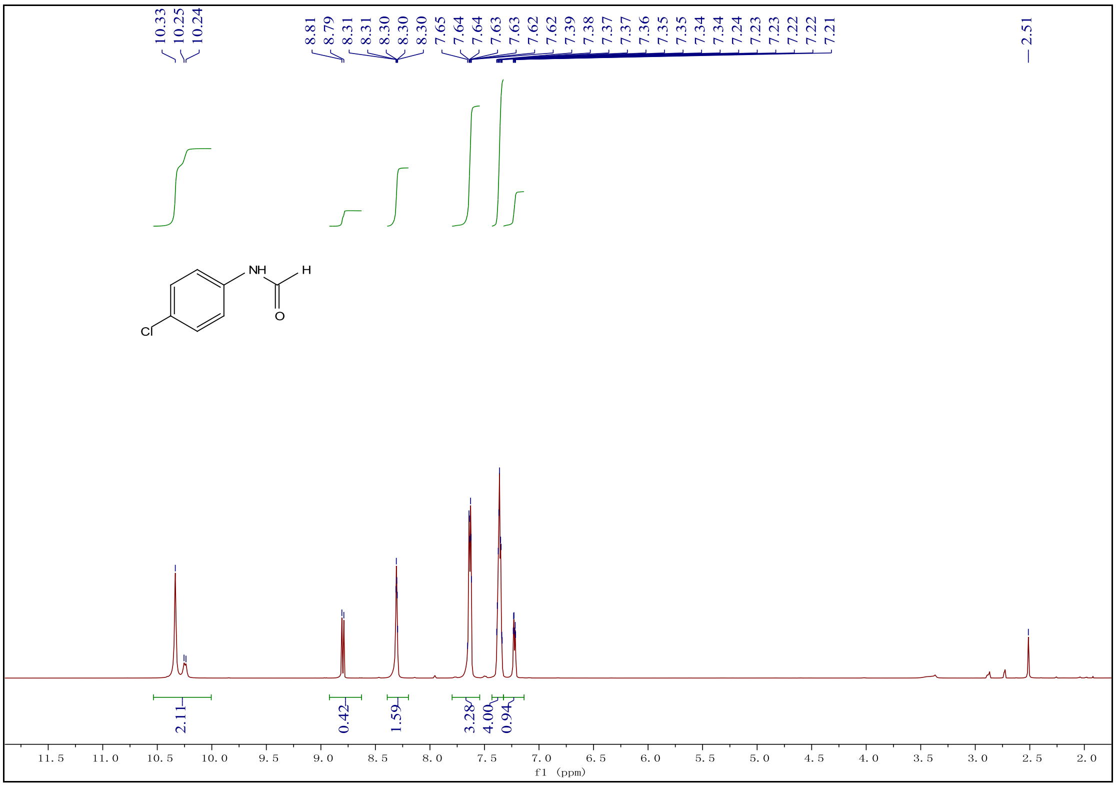

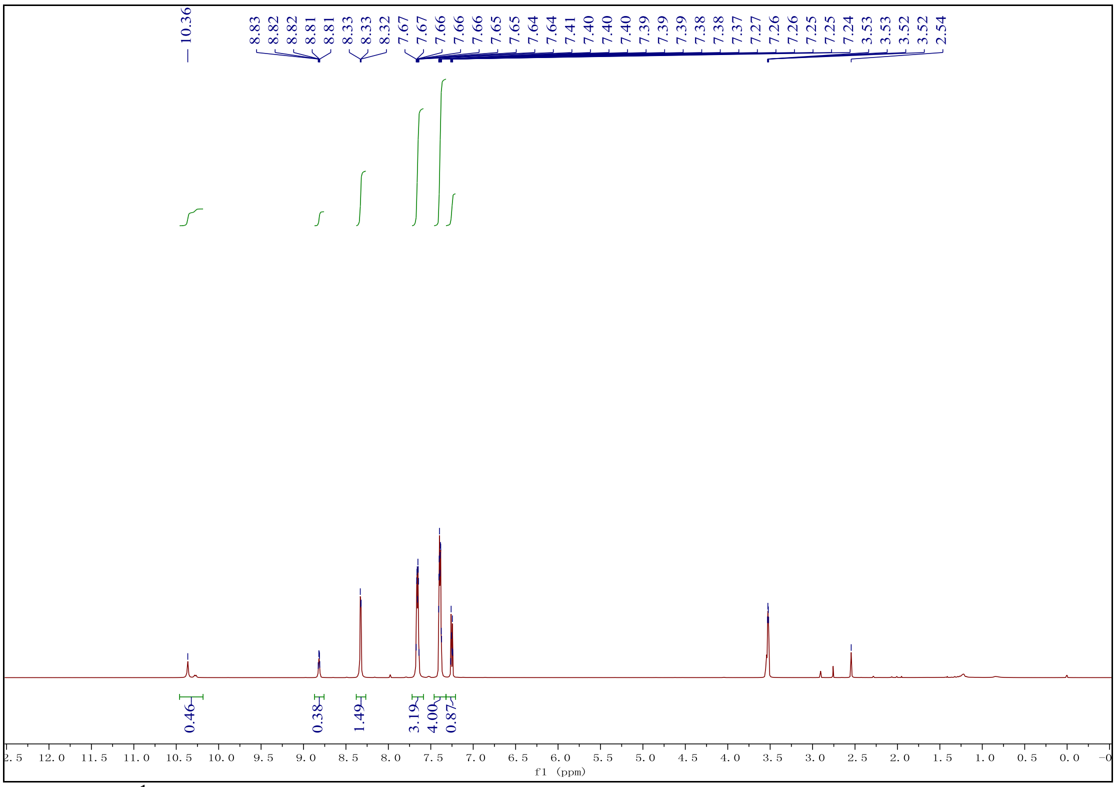

Figure 49. ^{1}H NMR spectrum of **2b** (Solvent: DMSO-d6)

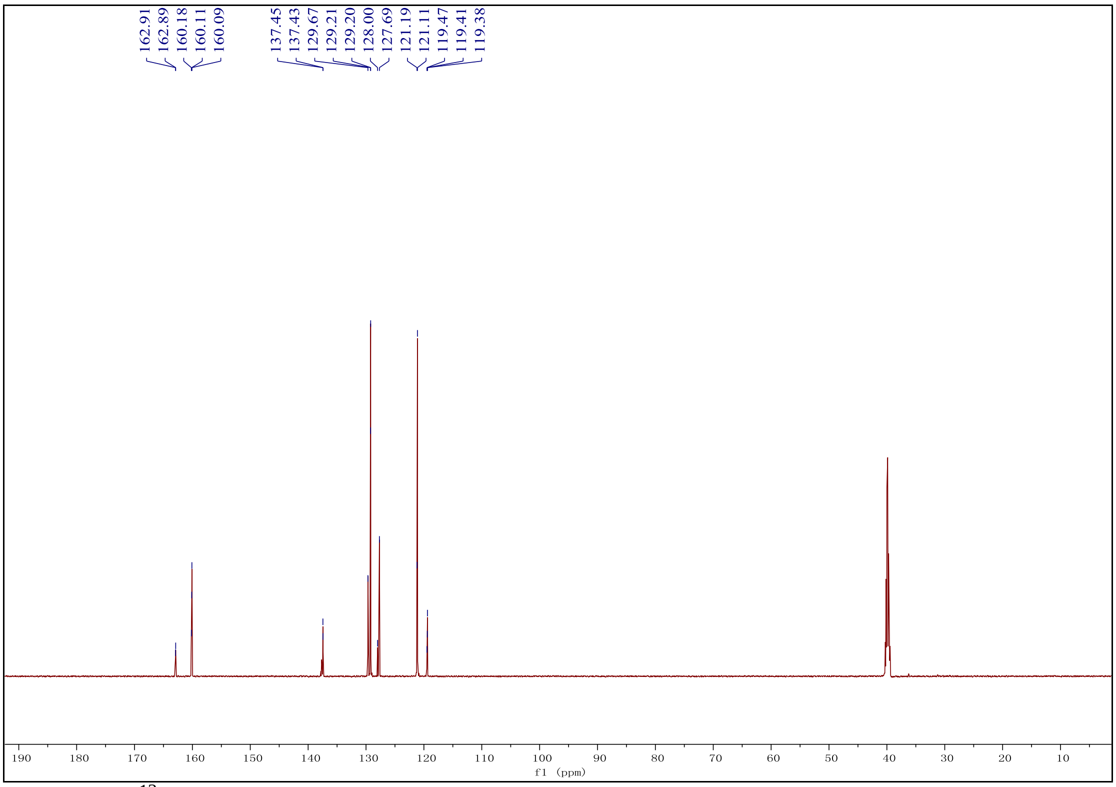

Figure 50. ^{13}C NMR spectrum of **2b** (Solvent: DMSO-d6)

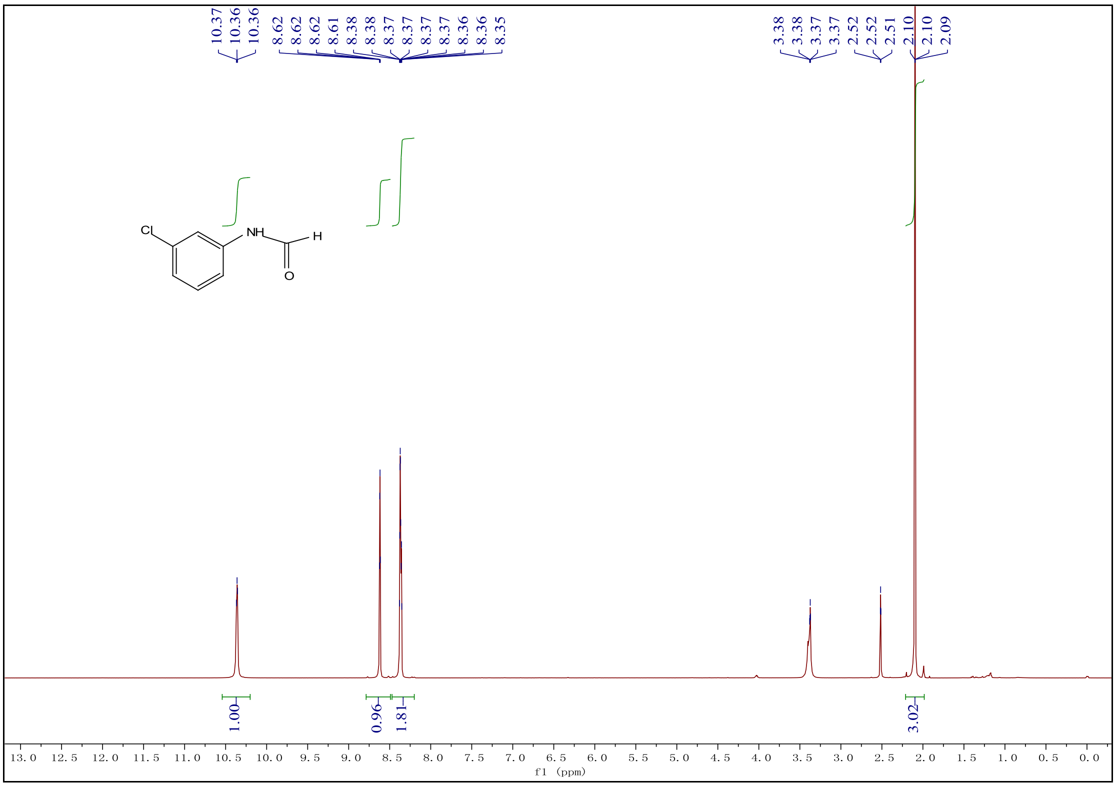

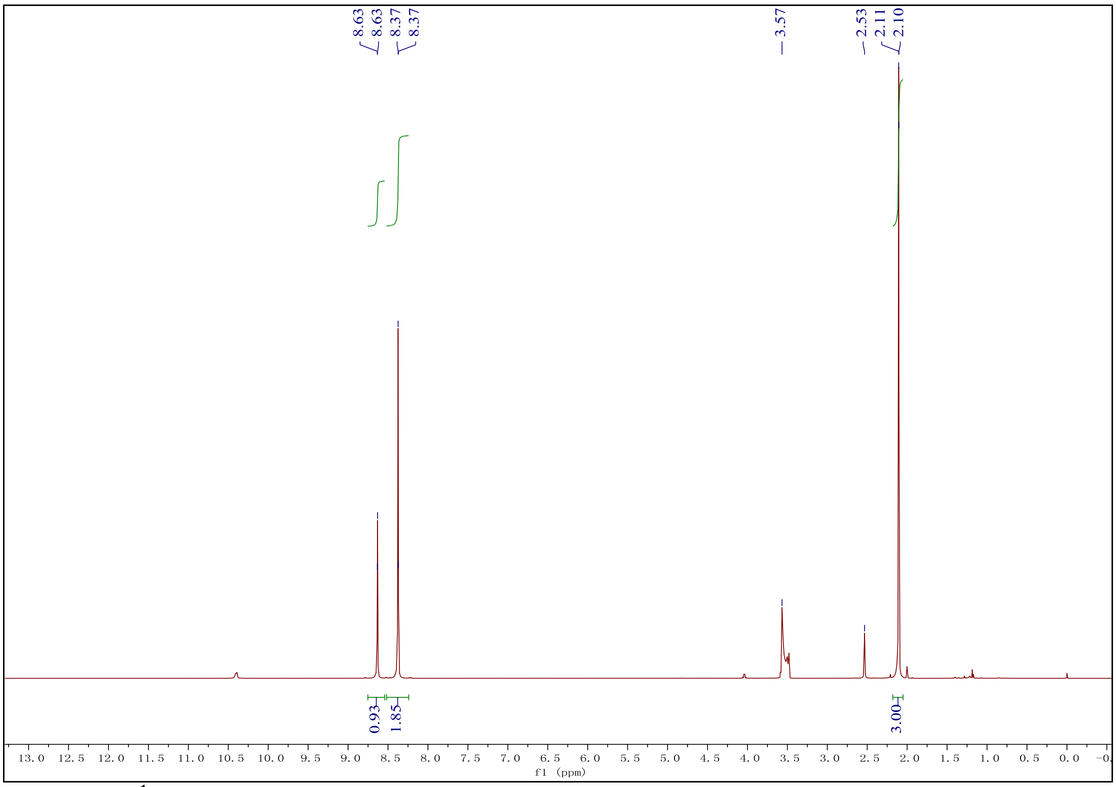

Figure 51. ^{1}H NMR spectrum of **2c** (Solvent: DMSO-d6)

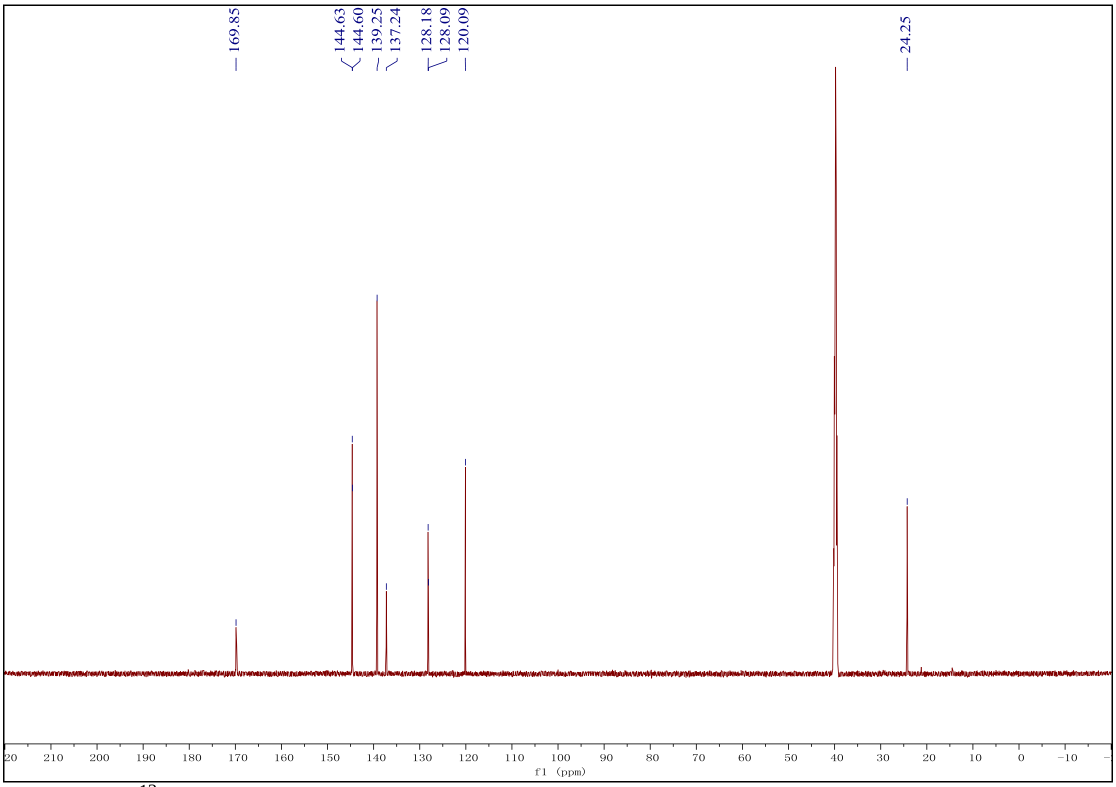

Figure 52. ^{13}C NMR spectrum of **2c** (Solvent: DMSO-d6)

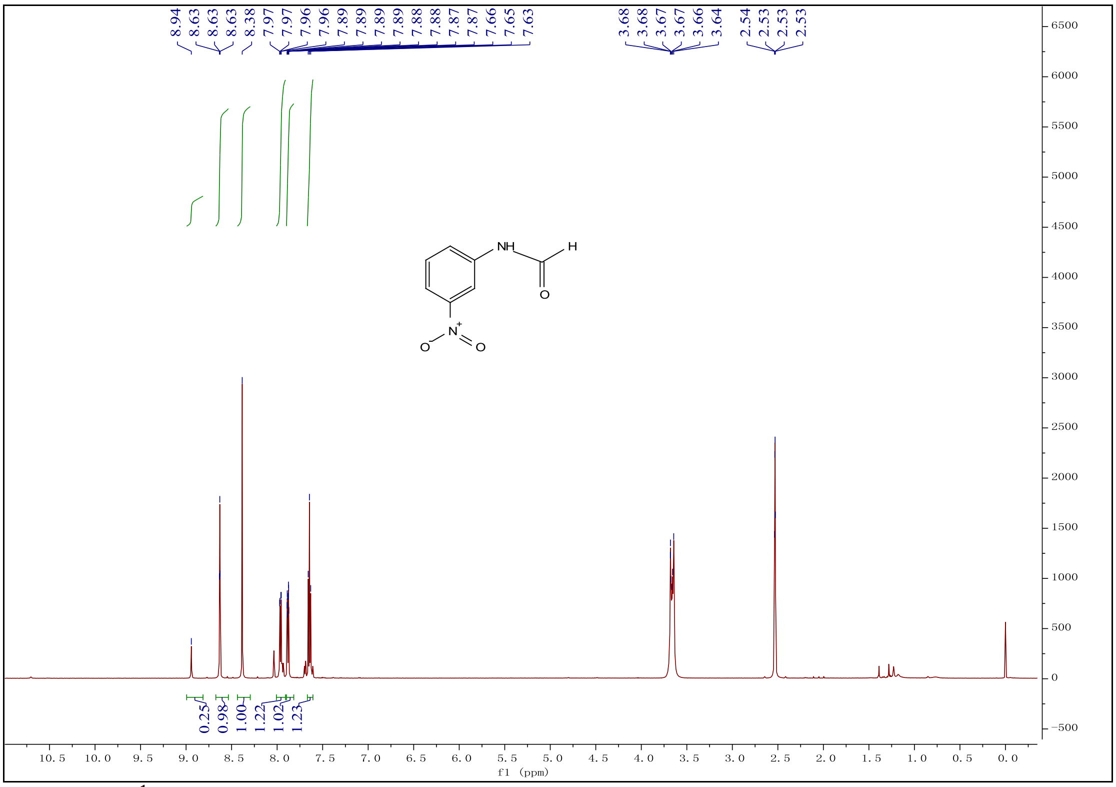

Figure 53. ^{1}H NMR spectrum of **2d** (Solvent: DMSO-d6)

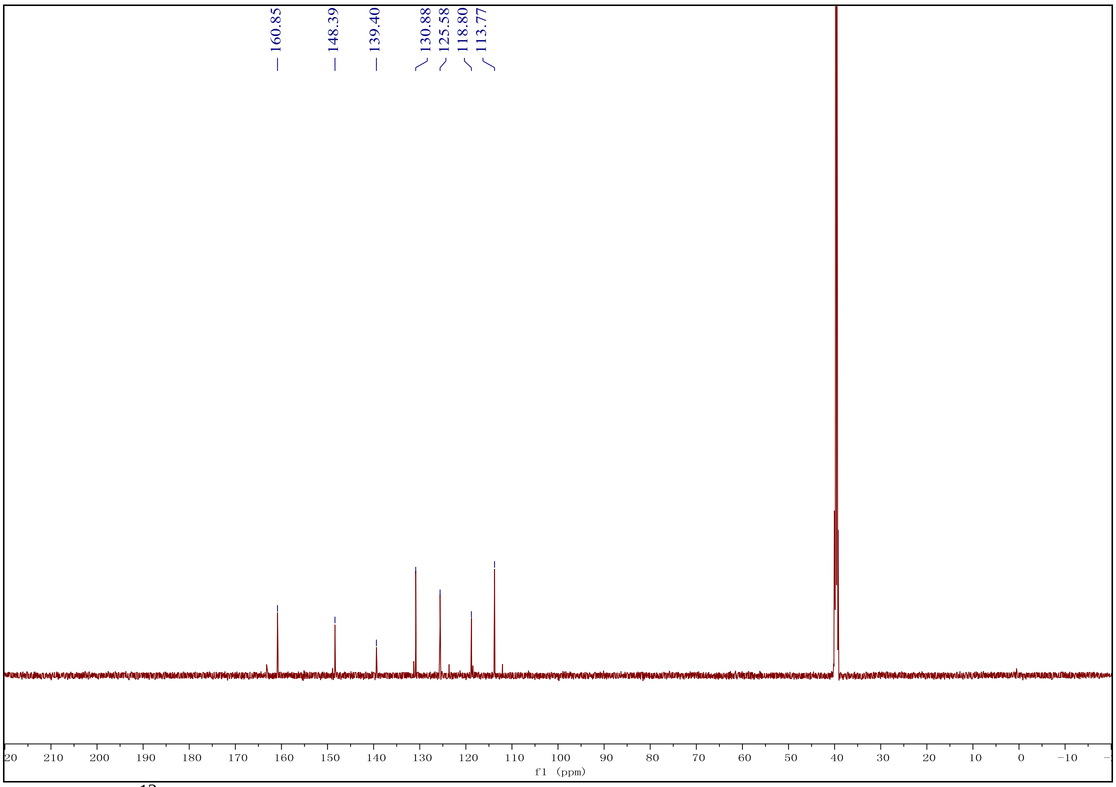

Figure 54. ^{13}C NMR spectrum of **2d** (Solvent: DMSO-d6)

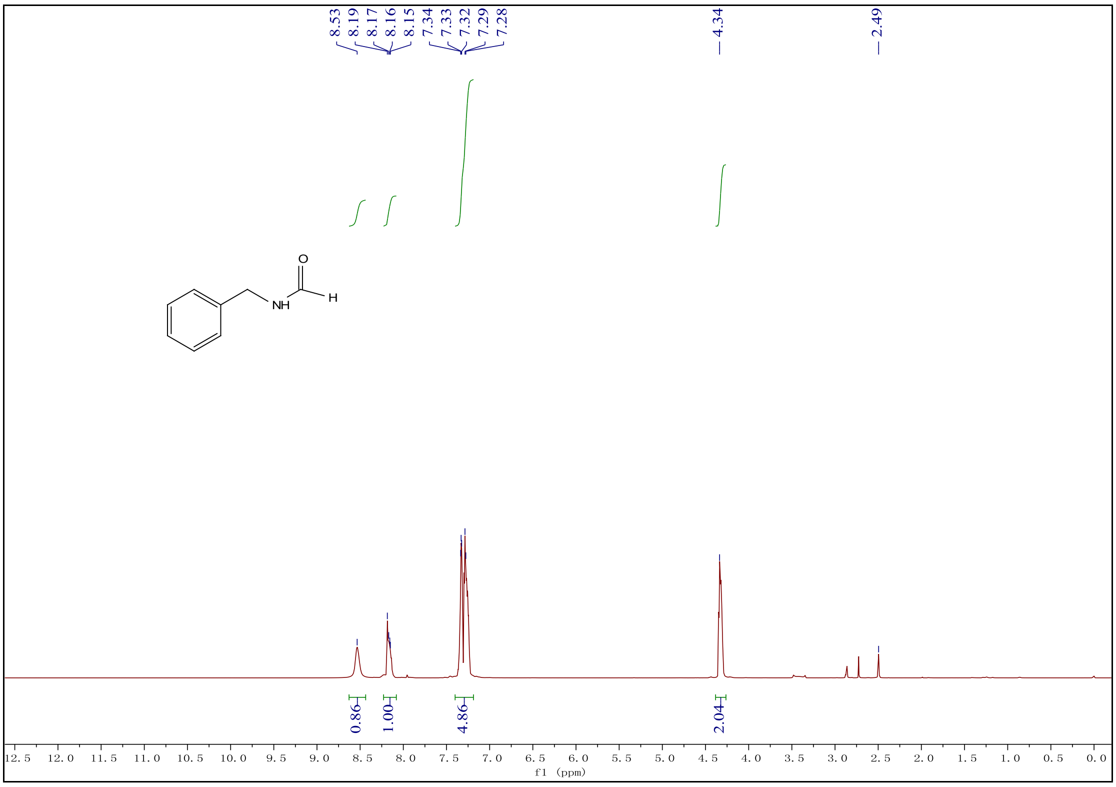

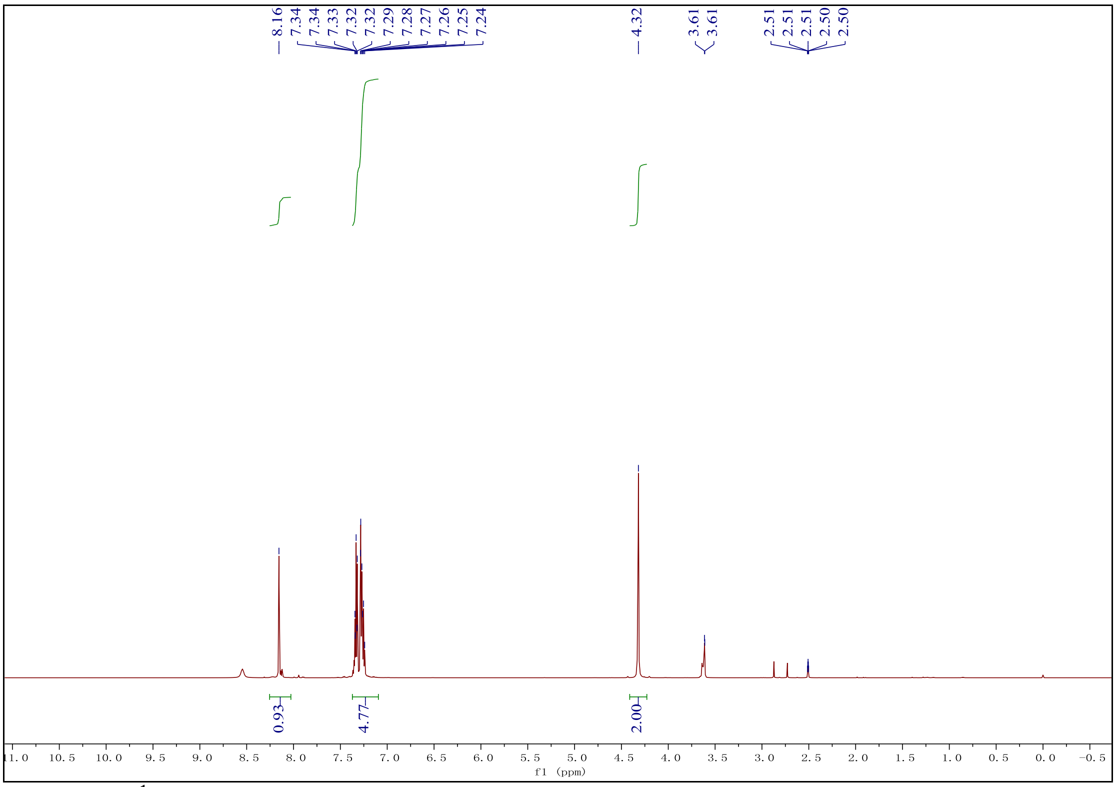

Figure 55. ^{1}H NMR spectrum of **2e** (Solvent: DMSO-d6)

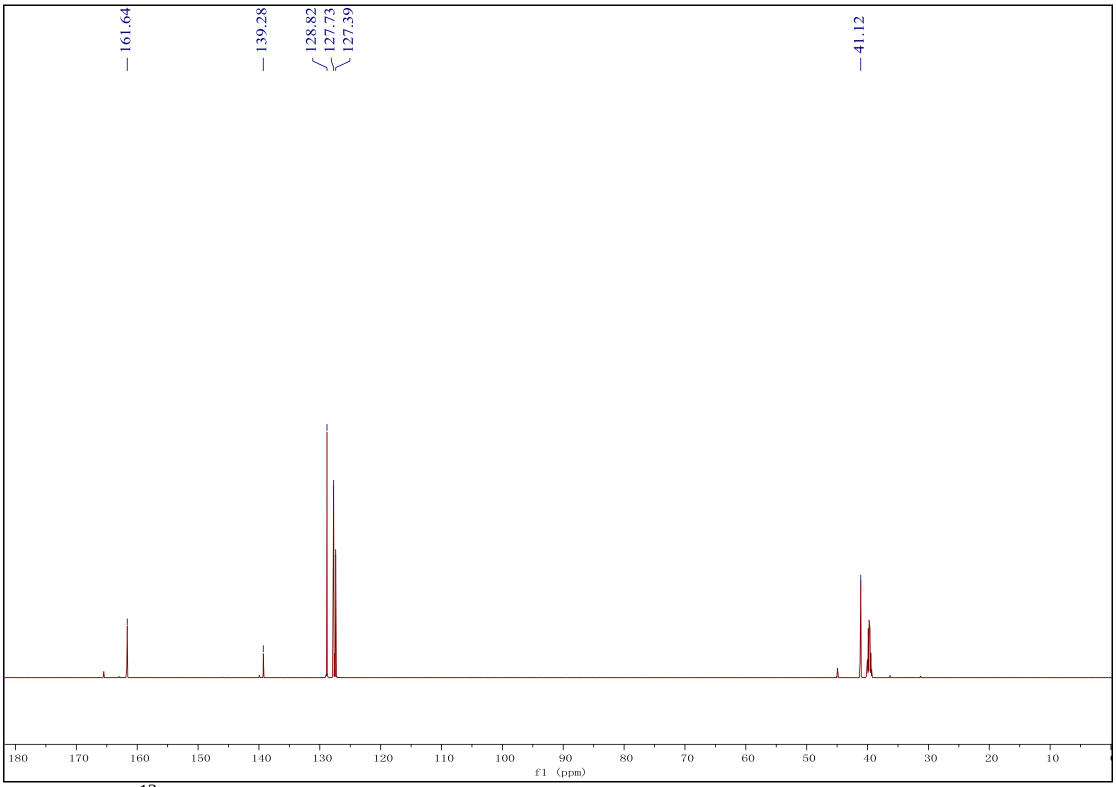

Figure 56. ^{13}C NMR spectrum of **2e** (Solvent: DMSO-d6)

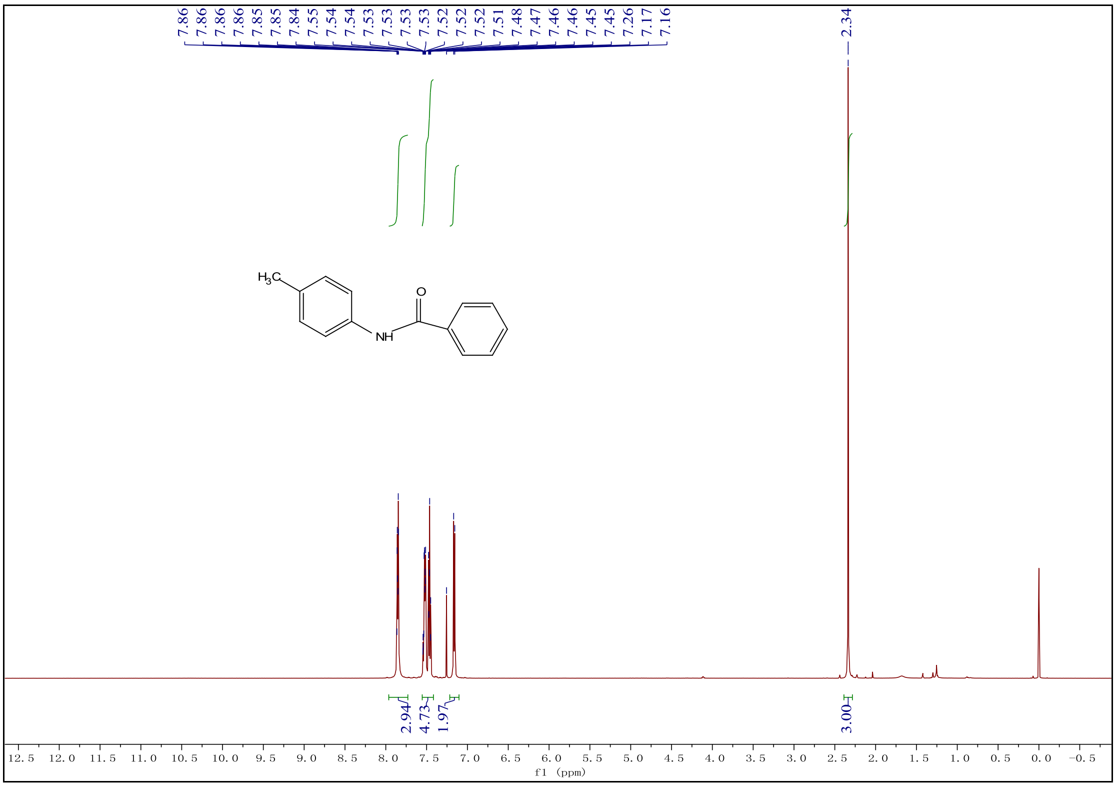

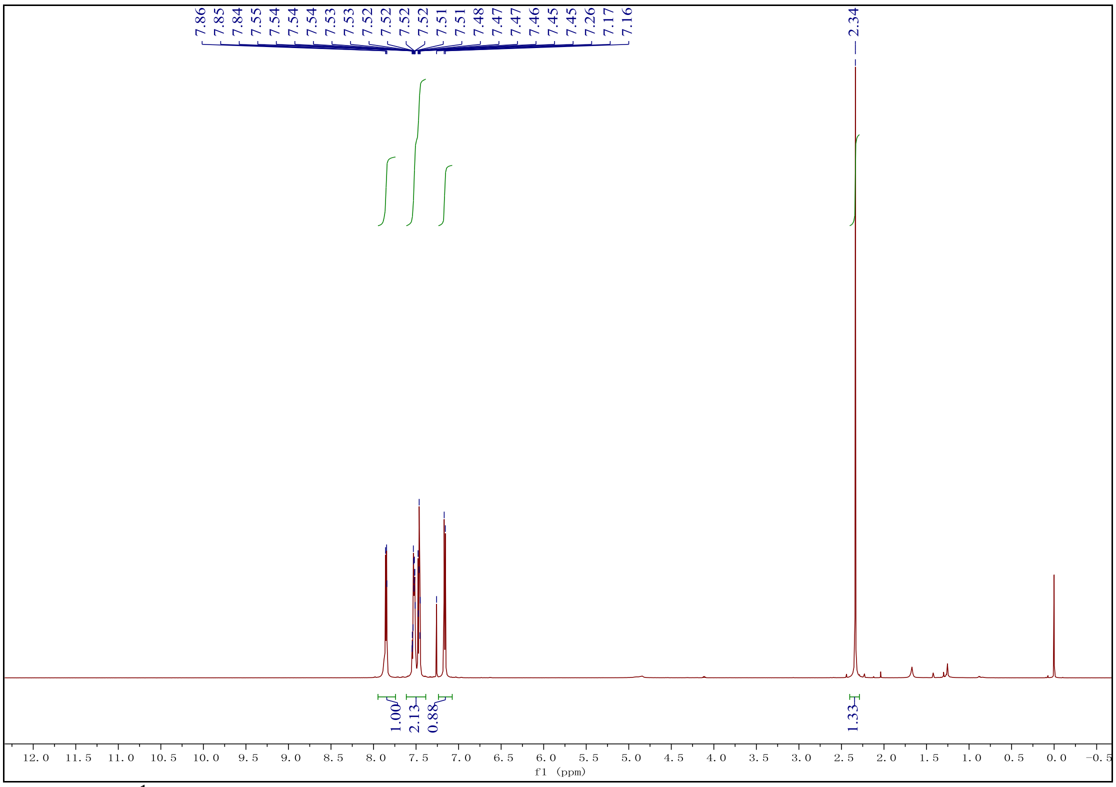

Figure 57. ^{1}H NMR spectrum of **3a** (Solvent: $CDCl_3$)

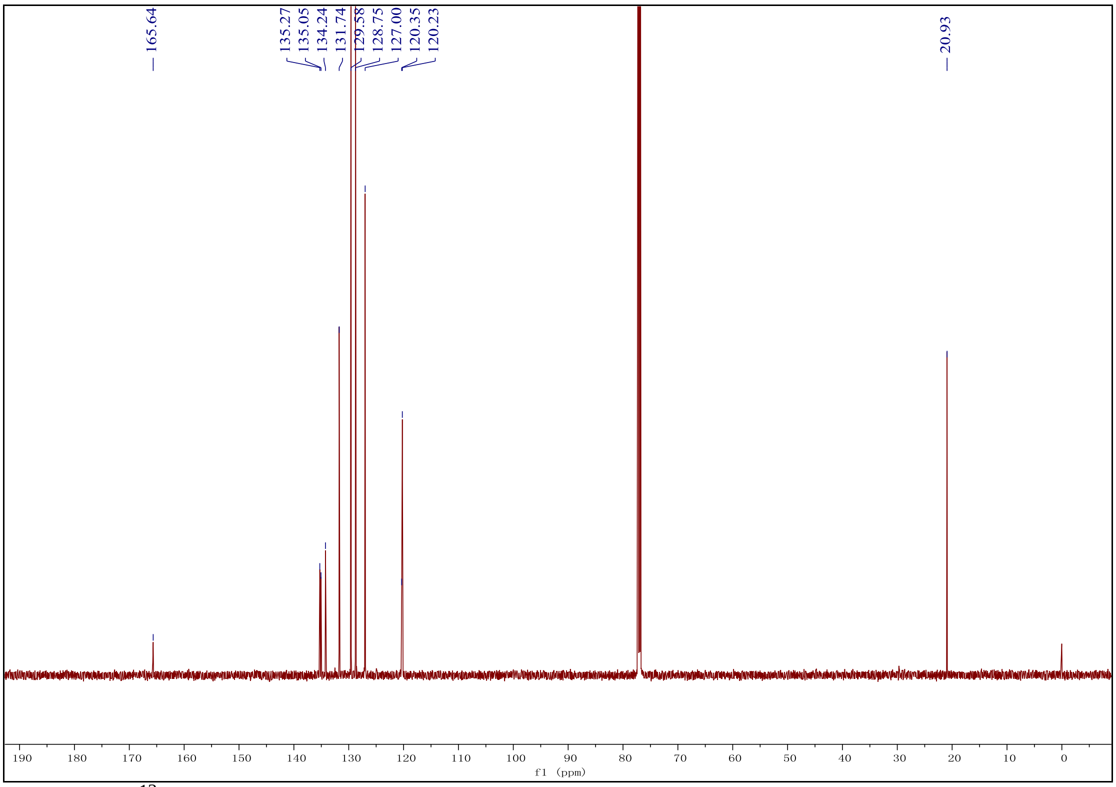

Figure 58. ^{13}C NMR spectrum of **3a** (Solvent: $CDCl_3$)

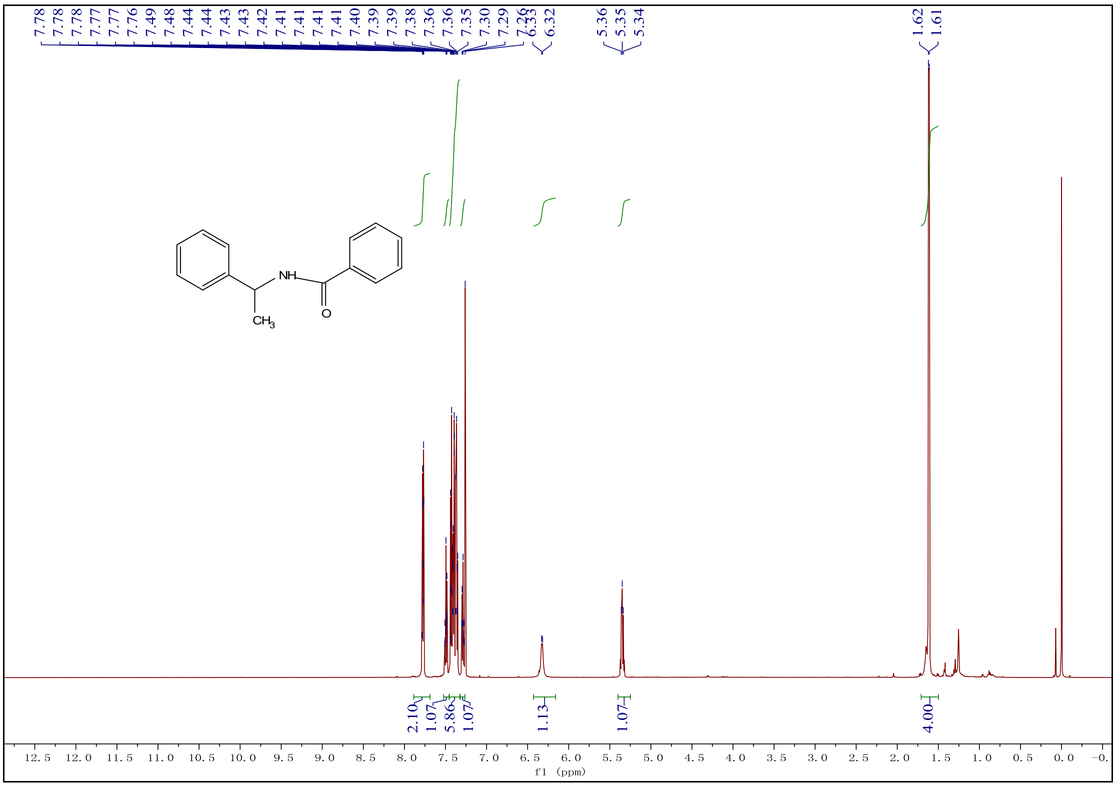

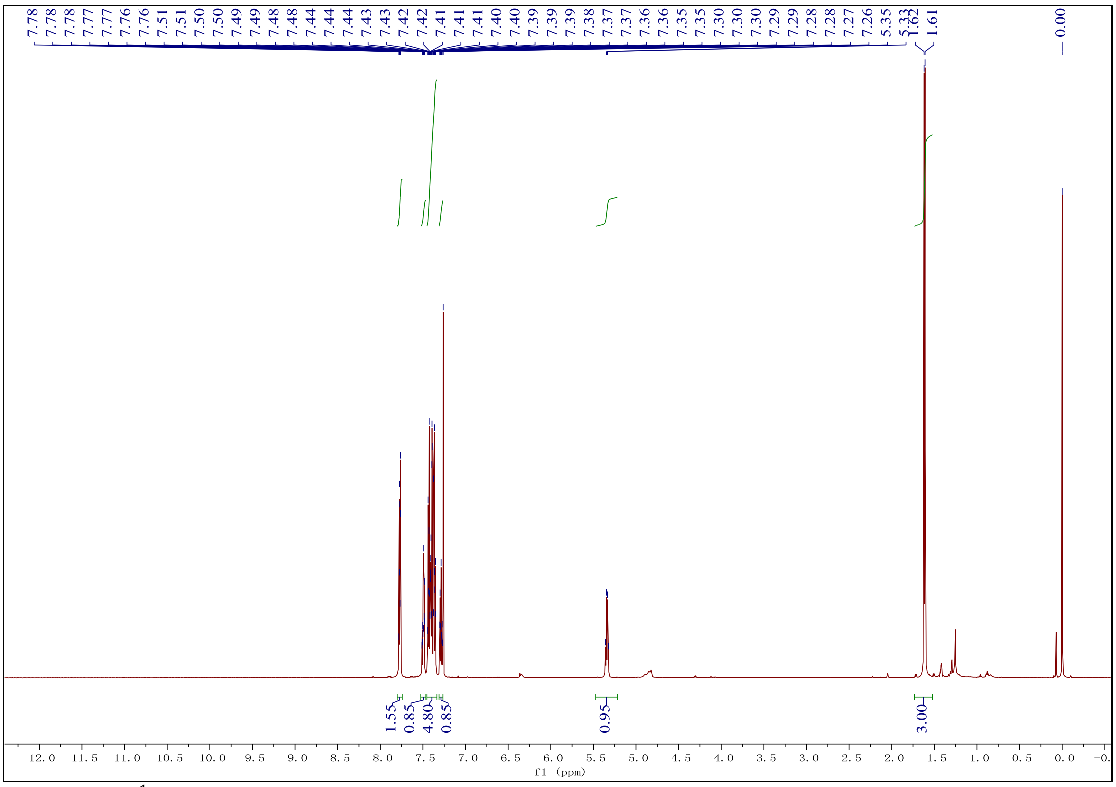

Figure 59. ^{1}H NMR spectrum of **3b** (Solvent: $CDCl_3$)

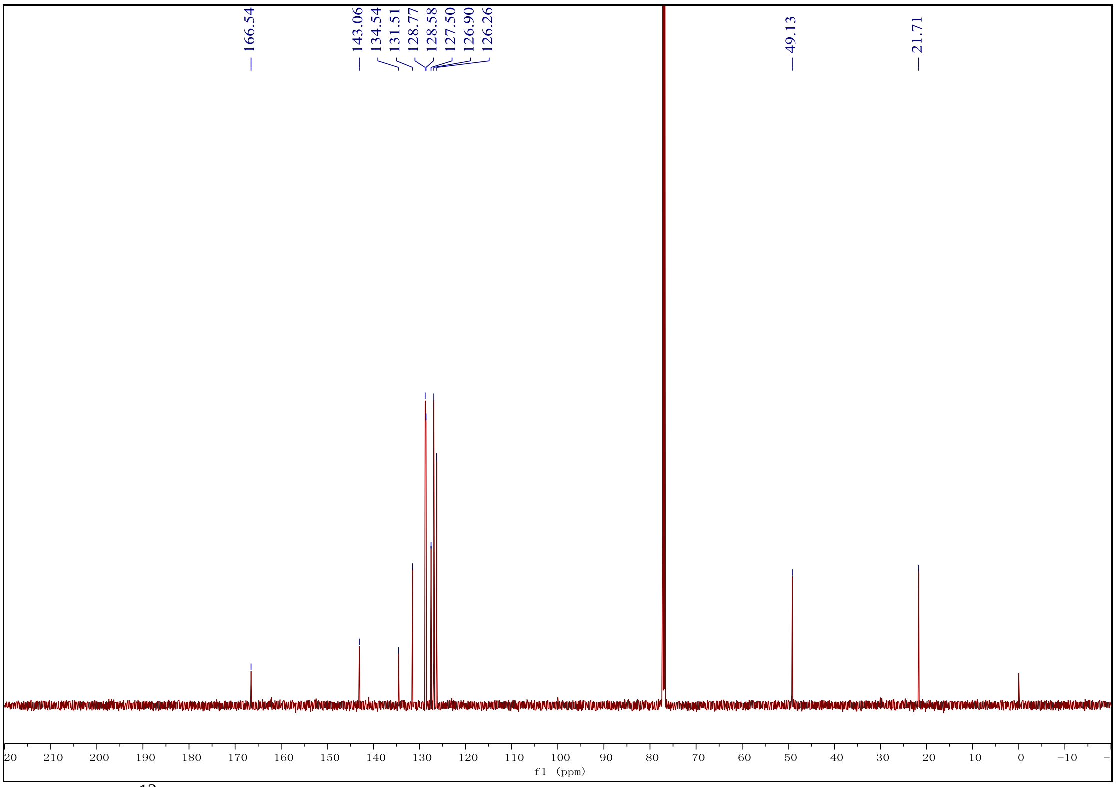

Figure 60. ^{13}C NMR spectrum of **3b** (Solvent: $CDCl_3$)

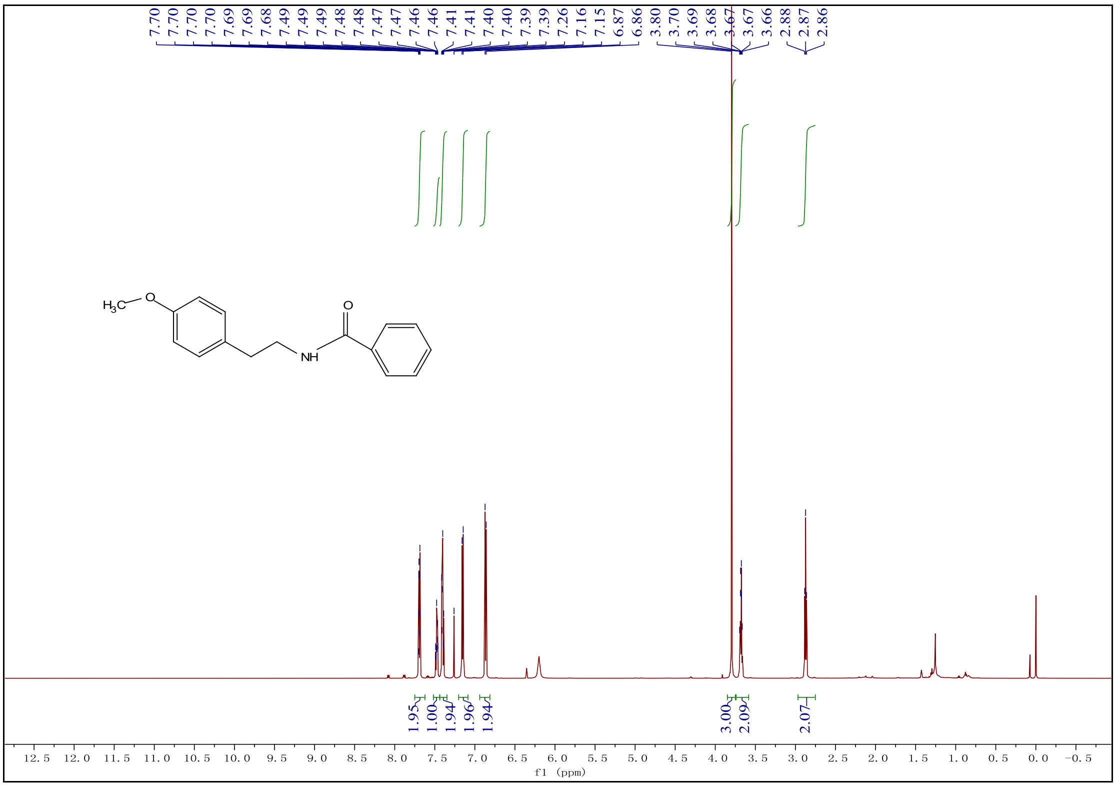

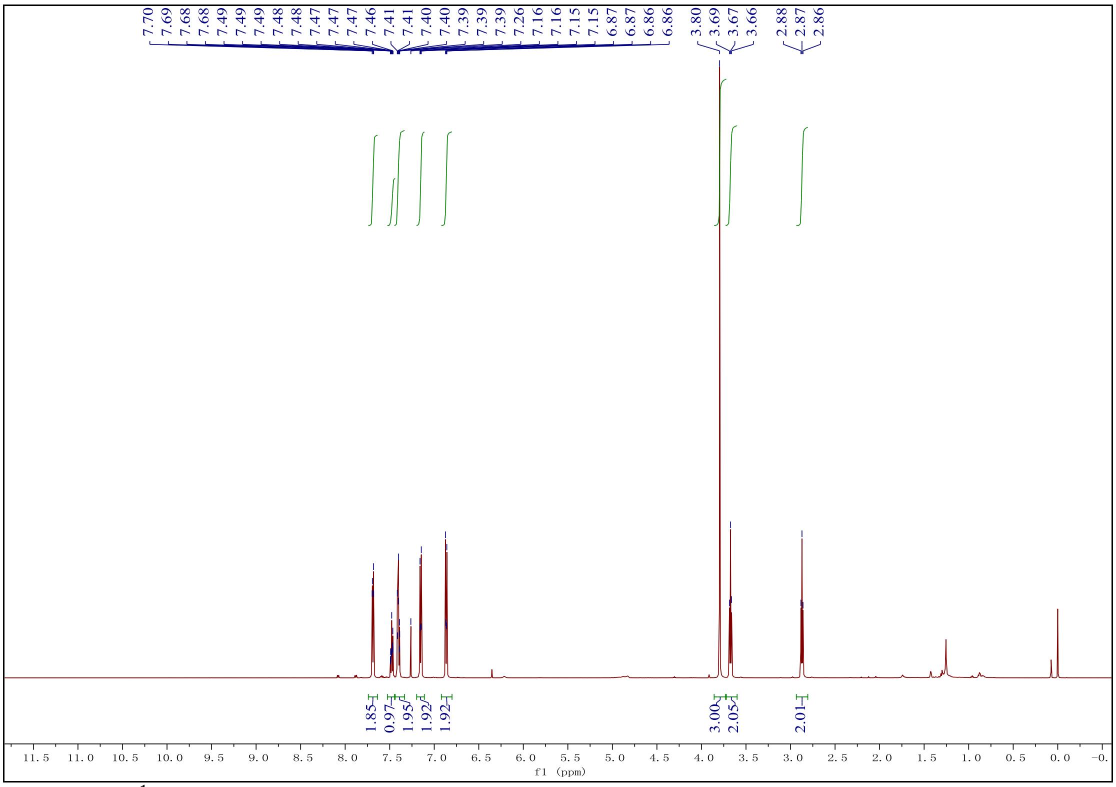

Figure 61. ^{1}H NMR spectrum of **3c** (Solvent: $CDCl_3$)

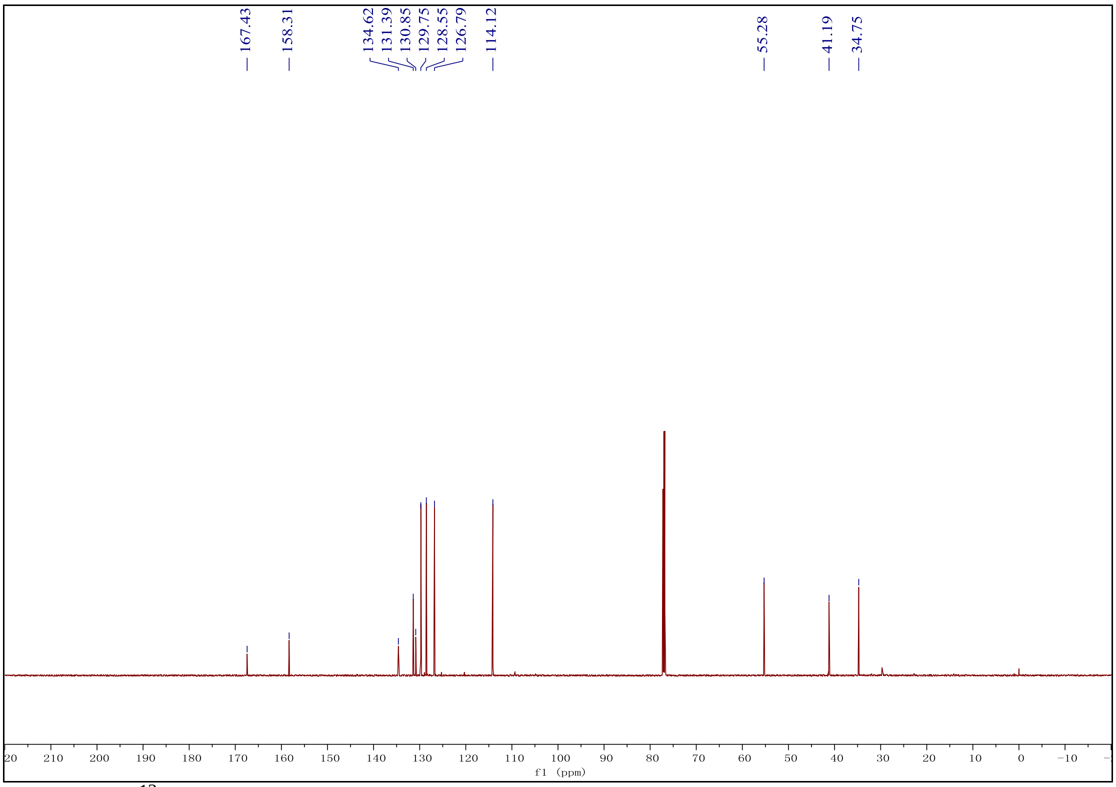

Figure 62. ^{13}C NMR spectrum of **3c** (Solvent: $CDCl_3$)

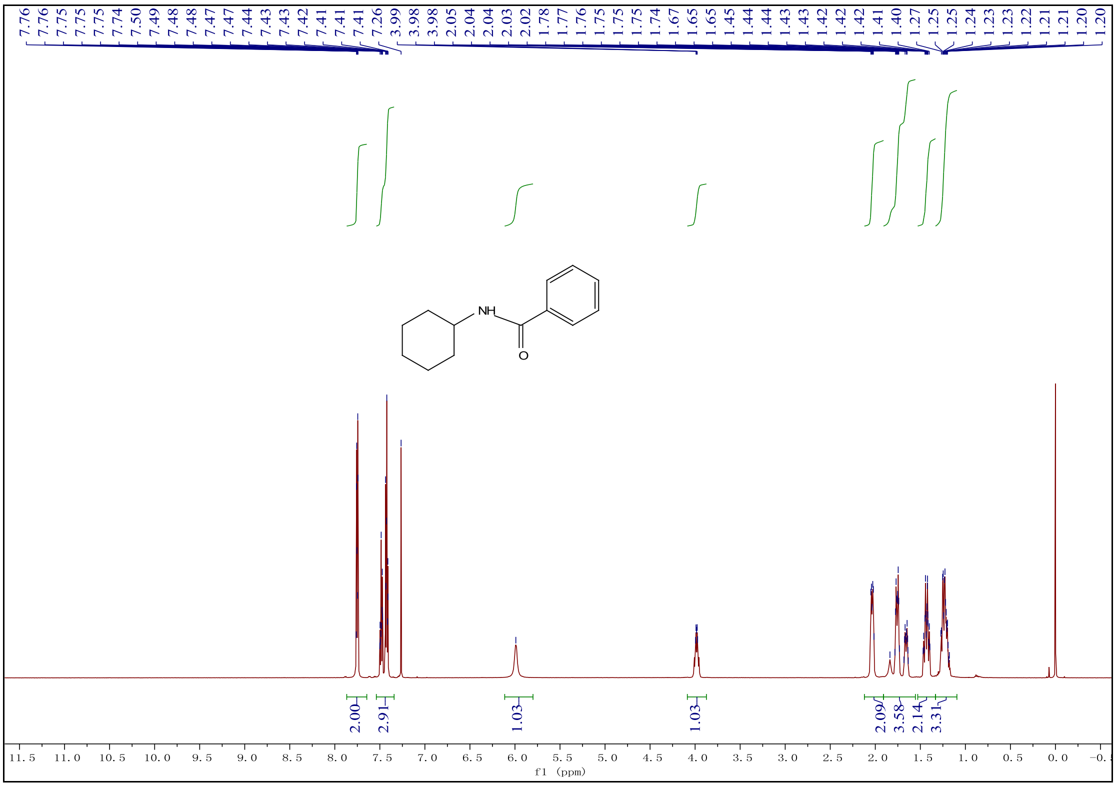

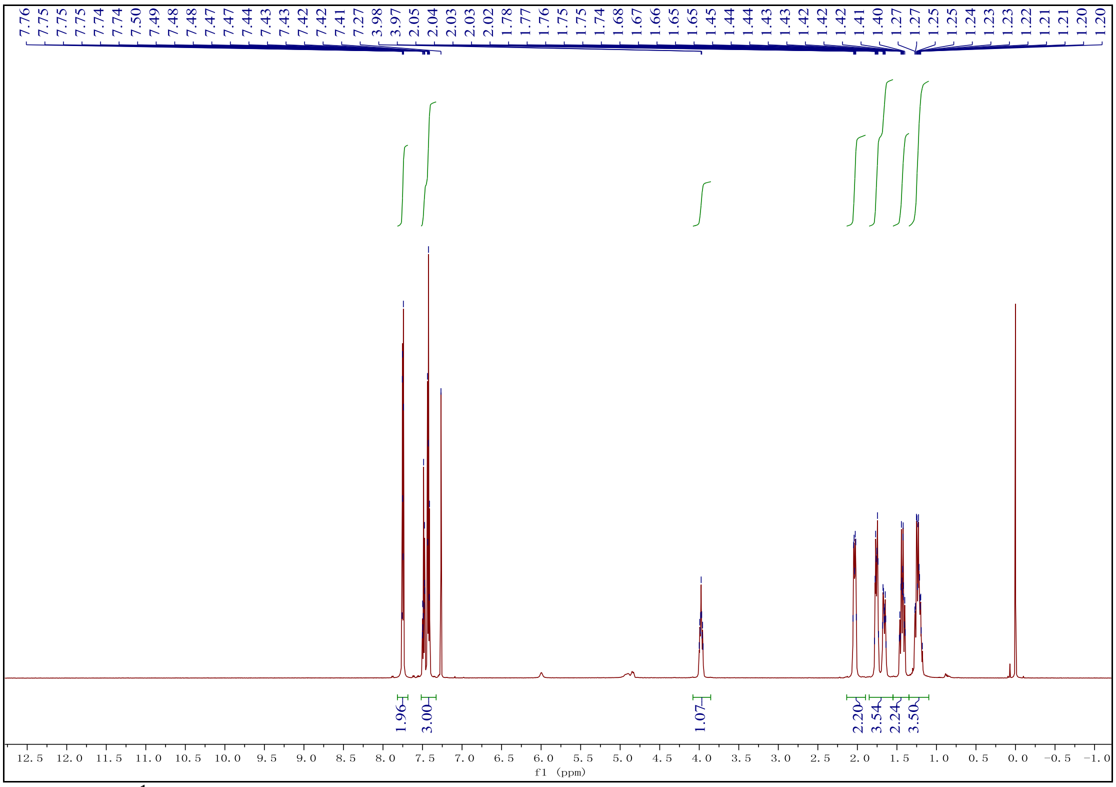

Figure 63. ^{1}H NMR spectrum of **3d** (Solvent: $CDCl_3$)

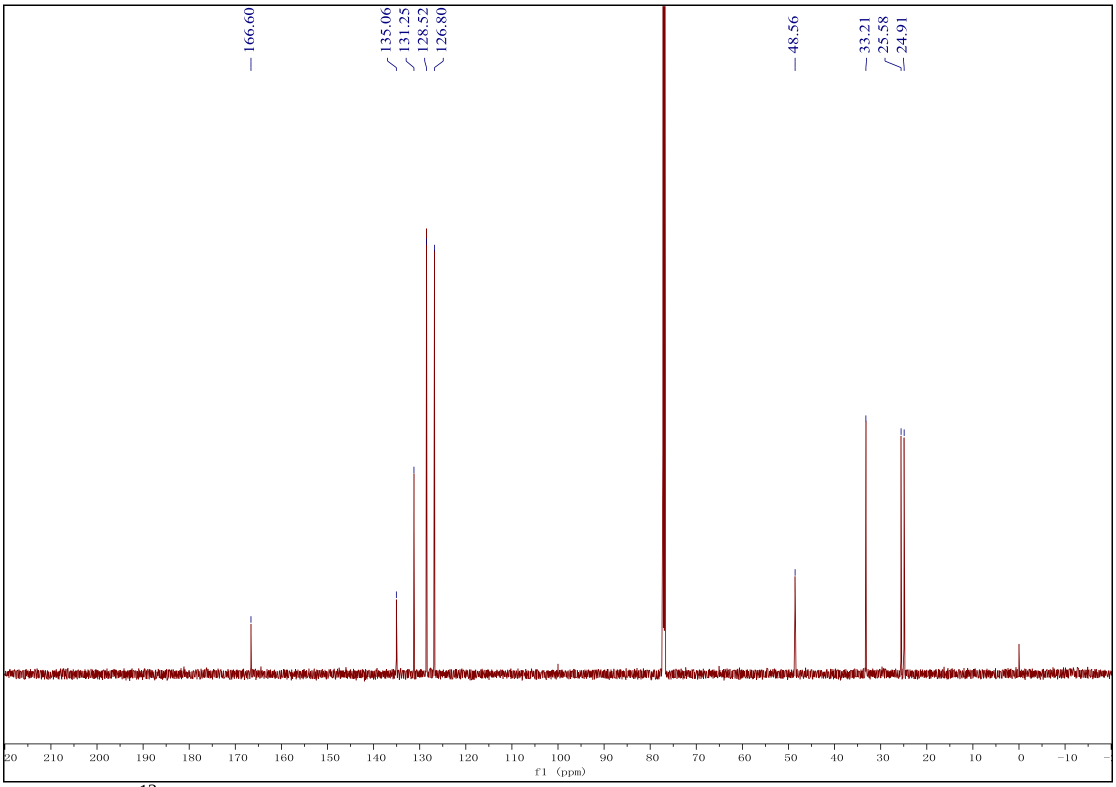

Figure 64. ^{13}C NMR spectrum of **3d** (Solvent: $CDCl_3$)
